# Data-driven multi-scale mathematical modeling of SARS-CoV-2 infection reveals heterogeneity among COVID-19 patients

**DOI:** 10.1371/journal.pcbi.1009587

**Published:** 2021-11-24

**Authors:** Shun Wang, Mengqian Hao, Zishu Pan, Jinzhi Lei, Xiufen Zou

**Affiliations:** 1 School of Mathematics and Statistics, Wuhan University, Wuhan, China; 2 Hubei Key Laboratory of Computational Science, Wuhan University, Wuhan, China; 3 State Key Laboratory of Virology, College of Life Sciences, Wuhan University, Wuhan, China; 4 School of Mathematical Sciences, Center for Applied Mathematics, Tiangong University, Tianjin, China; University of Tennessee Health Science Center College of Medicine Memphis, UNITED STATES

## Abstract

Patients with coronavirus disease 2019 (COVID-19) often exhibit diverse disease progressions associated with various infectious ability, symptoms, and clinical treatments. To systematically and thoroughly understand the heterogeneous progression of COVID-19, we developed a multi-scale computational model to quantitatively understand the heterogeneous progression of COVID-19 patients infected with severe acute respiratory syndrome (SARS)-like coronavirus (SARS-CoV-2). The model consists of intracellular viral dynamics, multicellular infection process, and immune responses, and was formulated using a combination of differential equations and stochastic modeling. By integrating multi-source clinical data with model analysis, we quantified individual heterogeneity using two indexes, i.e., the ratio of infected cells and incubation period. Specifically, our simulations revealed that increasing the host antiviral state or virus induced type I interferon (IFN) production rate can prolong the incubation period and postpone the transition from asymptomatic to symptomatic outcomes. We further identified the threshold dynamics of T cell exhaustion in the transition between mild-moderate and severe symptoms, and that patients with severe symptoms exhibited a lack of naïve T cells at a late stage. In addition, we quantified the efficacy of treating COVID-19 patients and investigated the effects of various therapeutic strategies. Simulations results suggested that single antiviral therapy is sufficient for moderate patients, while combination therapies and prevention of T cell exhaustion are needed for severe patients. These results highlight the critical roles of IFN and T cell responses in regulating the stage transition during COVID-19 progression. Our study reveals a quantitative relationship underpinning the heterogeneity of transition stage during COVID-19 progression and can provide a potential guidance for personalized therapy in COVID-19 patients.

## Author summary

Coronavirus disease 2019 (COVID-19) is currently destroying both lives and economies. However, patients infected with severe acute respiratory syndrome (SARS)-like coronavirus (SARS-CoV-2) usually present heterogeneous and complicated progressions, such as different incubation periods (short and long), symptoms (asymptomatic and symptomatic) and severity (mild-moderate and severe). Currently, various clinical data and experimental data are available from different countries, which has great significance for integrating different types of data to comprehensively understand the diverse disease progression in COVID-19 patients and guide individual treatment strategies. Here, we developed a multi-scale computational model to describe the dynamical process of patients infected with SARS-CoV-2, including intracellular viral dynamics, multicellular infection process, and immune responses. By combining data integration, stochastic simulation and quantitative analysis based on the multi-scale mathematical model, we addressed an important question regarding how IFN response and T cell exhaustion quantitatively affect heterogeneous progression in patients with respect to incubation periods, symptoms and severity. Furthermore, the efficacy of various therapeutic strategies for treating COVID-19 patients with different severity degrees was evaluated and validated. The computational framework in this study can also be extended to explore the dynamical process of other coronavirus infections.

## Introduction

Coronavirus disease 2019 (COVID-19), which is caused by the novel severe acute respiratory syndrome (SARS)-like coronavirus (SARS-CoV-2), is currently destroying global health and economies. Patients with COVID-19 exhibit different disease symptoms, including mild, moderate and severe cases [1, 2]. The severity of disease in infected individuals correlates with the numbers of immune cells (CD4^+^ and CD8^+^ T cells, B cells and natural killer cells [3], as well as serum levels of pro-inflammatory cytokines (IL-6, TNF, etc.) characterized as a cytokine storm [1, 2, 4, 5]. Importantly, the diverse incubation periods of SARS-CoV-2 infection in different patients make it incredibly difficult to predict the disease progression or to initiate clinical treatment on time [6, 7]. The mean incubation period of SARS-CoV-2 is estimated to be 3–7 days [8, 9], and asymptomatic COVID-19 patients effectively transmit SARS-CoV-2 during their incubation periods [10].

There are many cellular and molecular factors that influence COVID-19 severity. Angiotensin-converting enzyme-2 (ACE2), the functional receptor of SARS-CoV-2, plays a crucial role in the pathogenesis of COVID-19 by allowing viral entry into human cells [11]. ACE2 is highly expressed on target cells, including absorptive enterocytes and epithelial cells [11–13]. During viral infection, the spike (S) protein of CoV-2 interacts with ACE2, and the cellular transmembrane serine protease 2 (TMPRSS2) [14] mediates the viral envelope to host cell membrane fusion, leading to the release of viral nucleocapsid into the cytoplasm of host cells. After viral infection, cellular detection of viral replication is largely mediated by a family of intracellular pattern recognition receptors (PRRs) that sense aberrant RNA structures [15], resulting in the engagement of cellular antiviral defenses [16]. However, high IL-6 levels are associated with severe disease and death [17, 18], whereas the expression of interferon-*γ* (IFN-*γ*) tends to be slightly lower in severe cases than in moderate cases, primarily due to the decrease in CD4^+^, CD8^+^ T cells and NK cells [19]. Total T cells, and CD4^+^ and CD8^+^ T cell counts are negatively correlated with serum IL-6, IL-10, and TNF-*α* levels in COVID-19 patients, and patients in the disease resolution period exhibit decreased IL-6, IL-10, and TNF-*α* concentrations and restored T cell counts [20]. Therefore, IL-6 and IL-10 can be used as predictors for rapid prognosis of COVID-19 patients with higher risk of disease deterioration, and the neutrophil-to-lymphocyte ratio and neutrophil-to-CD8^+^ T cell ratio have been identified as powerful predictors of severe COVID-19 [21].

COVID-19 progression involves multiple complex steps of virus-host interactions. COVID-19 in different individuals exhibits diverse severity, including asymptomatic and symptomatic, mild, severe and critical, etc. [22], suggesting that individual heterogeneity is important for understanding the mechanism of COVID-19 and designing personalized treatment. Usually, clinical and biological experiments are not well poised to explore individual heterogeneity. To better understand the relationship between individual heterogeneity and disease severity in COVID-19 patients and to identify more effective treatments for different patients, in this study, we developed a data-driven multi-scale mathematical model to predict the clinical course of SARS-CoV-2 infection and quantitatively explored the factors underlying COVID-19 disease severity. By combining differential equations with stochastic modeling, as validated through different sources of experimental, epidemiological, and clinical data, we analyzed individual heterogeneity using stochastic simulation and quantitative analysis. Our results will contribute to understanding COVID-19 disease heterogeneity and to identifying novel clinical therapies.

## Results

### Host immune responses associated with COVID-19 severity based on multiple data analysis

**Fig 1:**
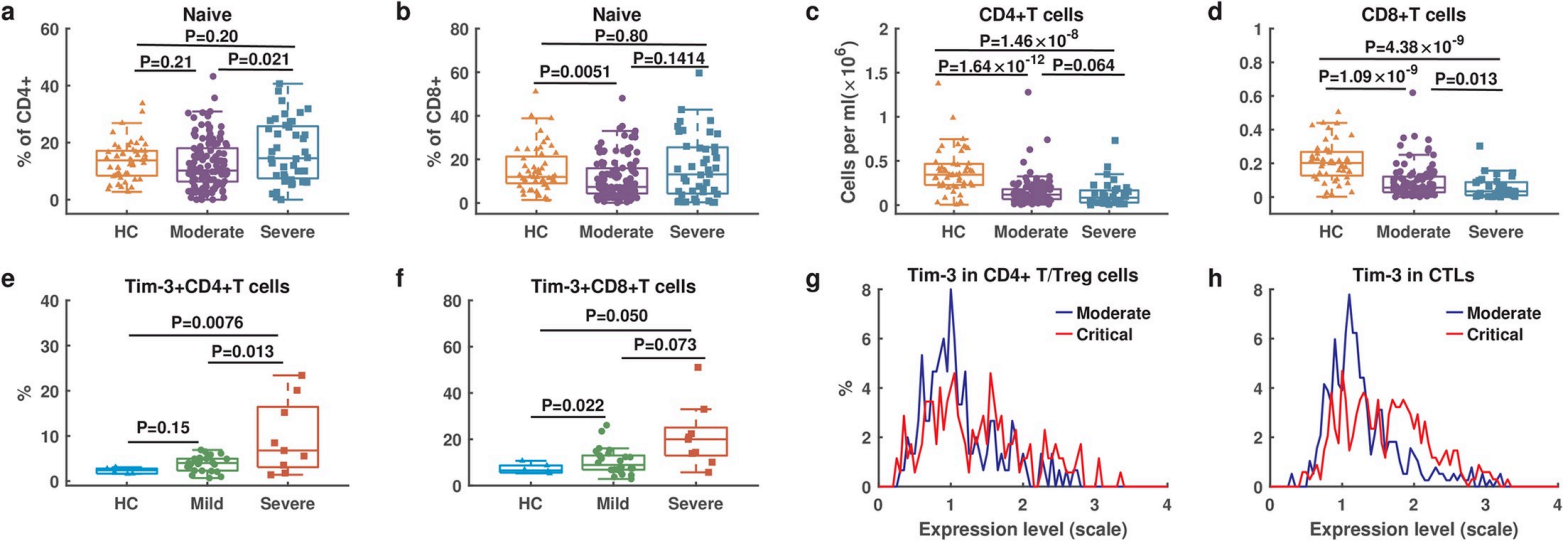
Multiple data on T cell response from COVID-19 patients with different symptoms. **a**. The percentage of naïve CD4^+^ T cells over CD4^+^ T cells. **b**. The percentage of naïve CD8^+^ T cells over CD8^+^ T cells. **c**. Counts of CD4^+^ T cells. **d**. Counts of CD8^+^ T cells. **a-d** include healthy control (HC: n = 50), moderate (n = 117) and severe (n = 40) patients from Yale New Haven Hospital (Dataset 4). **e**. The percentage of Tim-3 expression on CD4^+^ T cells. **f**. The percentage of Tim-3 on CD8^+^ T cells. **e-f** include healthy control (HC: n = 6), mild (n = 29) and severe (n = 12) patients from the Fifth Medical Center of PLA General Hospital of China (Dataset 2). **g**. The distribution of Tim-3 expression levels in CD4^+^ T/Treg cells. **h**. The distribution of Tim-3 expression levels in cytotoxic T lymphocytes (CTLs). **g-h** include moderate (n = 8) and critical (n = 13) patients from Charité-Universitätsmedizin Berlin and University Hospital Leipzig (Dataset 5). Significance was determined by two-sided, Wilcoxon rank-sum test.

To investigate host immune responses in COVID-19, we collected multiple datasets with adaptive immune cell counts, cytokines levels, proteomics, and single cell RNA-sequencing from COVID-19 patients with different symptoms (see Methods). First, we examined T cell response by comparing the counts of T cell subsets between 50 healthy donors and 157 COVID-19 patients (117 moderate and 40 severe symptoms) from Yale New Haven Hospital (Dataset 4) (Fig 1a–1d). The percentage of naïve CD4^+^ and CD8^+^ T cells were not significantly different between healthy donors and in severe patients (Fig 1a and 1b). Both CD4^+^ and CD8^+^ T cells exhibited significantly lower levels in patients than in healthy donors, and severe patients presented even lower level than that in moderate patients (Fig 1c and 1d).

Previous studies have shown that the reduction in T lymphocytes is linked to T cell exhaustion [19]. We examined the expression of the marker gene Tim-3 for T cell exhaustion in 41 COVID-19 patients from the Fifth Medical Center of PLA General Hospital of China (Dataset 2). The percentage of CD4^+^ and CD8^+^ T cells expressing Tim-3 on their surface was significantly higher in severe patients than in mild patients (Fig 1e and 1f). We further analyzed the distribution of Tim-3 transcription based on single cell RNA-sequencing data from 8 moderate patients and 13 critical patients from Charité-Universitätsmedizin Berlin and University Hospital Leipzig (Dataset 5). Critical patients exhibited significantly increased frequencies of cytotoxic T lymphocytes (CTLs) and regulatory T cells (CD4^+^T/Treg), and elevated Tim-3 transcription levels (Fig 1g and 1h), implying increased higher level T cell exhaustion in critical patients [23]. These data analyses suggest that exhaustion of T cells is associated with the reduction of T cells and accelerates the malignant development of COVID-19.

Previous studies have shown that a cytokine storm in COVID-19 may result in the emergence of severe patients and increase mortality [24–26]. We analyzed the published data of cytokine levels in COVID-19 patients from Datasets 3 and 4. The cytokines IL-6, IL-10, and IFN-*γ* were present at markedly higher levels in nonsurvivors patients than in survivors from the data of Renmin Hospital of Wuhan University (Fig Aa-c in S1 Text), and exhibited obvious increases from healthy donors and moderate patients, to severe patients from data of the Yale New Haven Hospital (Fig Ad-f in S1 Text). These results suggest that higher level cytokines such as IL-6, IL-10, and IFN-*γ* are associated with severe symptoms and death cases in COVID-19 patients [27]. Additional integrative data analysis is shown in Figs B and C in S1 Text (Section 1 in S1 Text for the detailed description).

Based on the above data analysis, we proposed three assumptions in model development: (1) the depletion of T cell counts is associated with T cell exhaustion; (2) T cell exhaustion is dependent on the density of cytokines because persistently high cytokine levels is known to induce T cell exhaustion [28]; (3) the comprehensive effect of IL-6, IL-10, and IFN-*γ* is represented by a single variable of cytokines, but the complex network that regulates cytokines activities is currently not well understood and is not included in this study.

### A multi-scale model of SARS-CoV-2 infection dynamics and host immune responses

**Fig 2:**
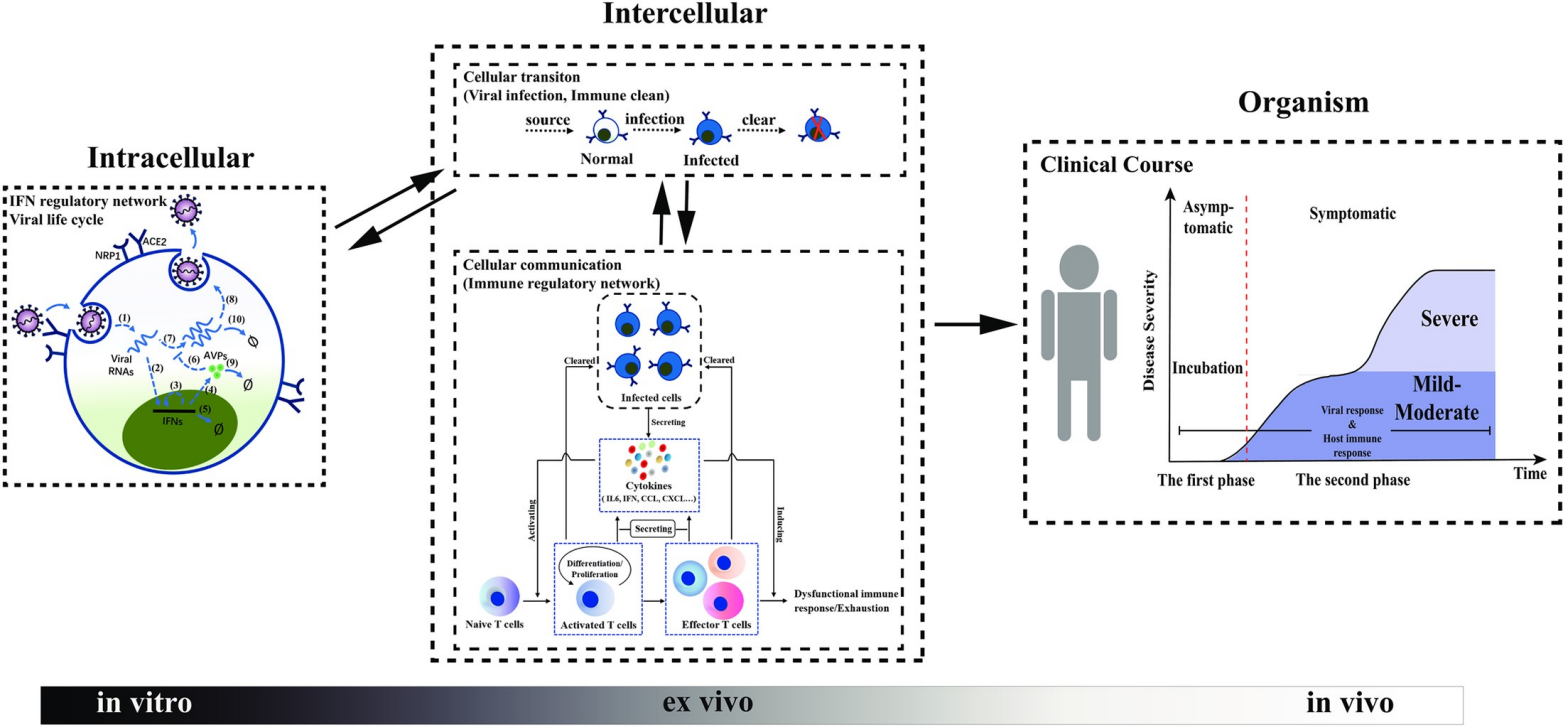
Framework of multiscale model of SARS-CoV-2 infection. **Intracellular**: The S protein of SARS-CoV-2 binds to receptor proteins (ACE2, NRP1) on the cell surface. Viral dynamics within a target cell are considered, which include (1) the release of RNA of SARS-CoV-2, (2) virus-activated IFN expression, (3) positive feedback of IFNs, (4) activation of AVPs by IFNs, (5) natural depletion of IFNs, (6) inhibition of the virus by AVPs, (7) viral RNA replication, (8) protein synthesis, assembly of novel SARS-CoV-2 and budding into the extracellular environment, (9) natural degradation of AVPs, and (10) degradation of viral RNA. The progeny viruses leave the target cell by budding and further infect additional susceptible cells. **Intercellular**: The status of target cells is divided into uninfected and infected. There is a supplied source of normal cells that will be transformed into infected cells if they are infected by the virus. The infected cell is identified and cleaned by effector T cells. With respect to cellular communication, T cells mediate the immune response to SARS-CoV-2. PRRs on the cell surface sense SARS-CoV-2 and activate the immune response. Immune cells secrete cytokines, such as IL-6, IL-10, IFN-*γ*, etc., and activate naïve T cells. The activated T cells undergo differentiation and proliferation, and emerge as effector T cells. Activated T cells and effector T cells clear the infected cells and secrete cytokines. Some of cytokines (pro-inflammatory cytokines) induce chronic inflammation, dysfunction of the immune response, and exhaustion of the effector T cells, which contribute to disease progression. **Organism**: The population size of infected cells dictates the progression and severity of COVID-19. The progression of COVID-19 is divided to two phases, symptomatic and asymptomatic. Furthermore, the severity of symptomatic patients is primarily divided into mild-moderate and severe.

To investigate disease progression in patients infected with SARS-CoV-2, we established a computational model that includes various scale dynamics. The model includes viral infection, viral spreading among multiple cells, and immune responses through IFN response, cytokines and effector T cells (Fig 2). The intracellular viral dynamics describes molecular-level events within individual cells, including the infection, binding, entry, replication of SARS-CoV-2, and IFN response signaling pathways (Fig 2 Intracellular), whereas the multicellular infection process describes the infectious cell-to-cell transmissions and the immune response that involves interactions between infected cells, cytokines, and T cell activities (Fig 2 Intercellular). The progression and severity of COVID-19 are dominated by the population size of infected cells (Fig 2 Organism).

SARS-CoV-2 primarily infects susceptible cells through receptor proteins (ACE2, NRP1). The CoV spike glycoprotein (S protein) of SARS-CoV-2 binds to receptors on the cell surface with high affinity, and the genomic RNA is released into the target cell. Inside the target cell, SARS-CoV-2 RNA employs organelles and synthases to complete viral replication and assembly, which results in a large number of newly synthesized viruses. Upon viral replication, the interferon signaling pathway, one of the virus-mediated innate immune signaling pathways, is activated and engages type I interferons (IFNs) and antiviral proteins (AVPs), and to restrict the process of viral replication [29]. The newly synthesized virus leaves the target cell by means of budding, and other susceptible cells are further infected, forming a cascade of cell infection [30] (Fig 2 Intracellular).

Intracellular SARS-CoV-2 RNAs are recognized by host pattern recognition receptors (PRRs), which triggers activation of the host immune response. The active immune cells secrete cytokines, such as IL-6, IFN, etc., and activate the naïve T cells. The active T cells undergo differentiation and proliferation to produce a large amount of activated T cells and effector T cells. These cells continuously clear the infected cells, and secrete inflammatory cytokines, which may lead to chronic inflammation [31]. The inflammatory microenvironment further induces dysfunctional immune responses and exhaustion of effector T cells [28] (Fig 2 Intercellular).

Herein, a mathematical model was established to describe the time evolution of the number of infected cells and the host immune response in accordance with the above process. In the model, we considered a system of multiple cells (here we assumed a constant cell number *N* for simplicity) that are potential target cells of SARS-CoV-2. The cells are heterogeneous with distinct levels of receptor proteins on their surface. Therefore, we have a set of (4*N* + 1) differential equations, which describe the dynamics of intracellular virus RNA concentrations $X_{\text{in}}^{i}\left(i=1,2,\cdots ,N\right)$, bounded cell surface receptor proteins *R*^*i*^(*i* = 1, 2, ⋯, *N*), IFN concentrations [IFNs]^*i*^(*i* = 1, 2, ⋯, *N*), AVP concentrations [AVPs]^*i*^(*i* = 1, 2, ⋯, *N*), and extracellular virus concentration *X*_ex_. Moreover, the infected cells (cell number *N*_infected_) promote the host immune response through the secretion of cytokines, which induces the production of effector T cells. The effector T cells clear the infected cells and secrete cytokines that induce further T cell exhaustion. The interactions among cytokine concentrations, effector T cells, and the infected cells are formulated as dynamic processes using two differential equations for among cytokine concentrations [Cytokines] and the effector T cells number [*T*_effector_]. Here, we assumed that the naïve T cells number remains constant throughout the process and that the total cell number is maintained at a constant level so that one uninfected cell is added to the system when an infected cell is cleaned by the effector T cells. The above assumptions lead to a system of (4*N* + 3) differential equations for the multiple scale process from viral dynamics to immune responses (see the Methods section for the detailed mathematical model and formulations).

Based on the dynamic model, we introduced a ratio of infected cells (*R*_IC_) to quantify disease progression after SARS-CoV-2 infection, which was defined as the ratio between the number of infected cells (*N*_infected_) to total cells (*N*) in the model, i.e.,

$$\begin{array}{c} R_{\text{IC}}=\frac{N_{\text{infected}}}{N} \end{array}$$

A summary of key parameters and their biological significance, effects, and clinical/experimental evidences are listed in Table 1.

**Table 1:** Summary of key parameters, biological significance, effects, and available clinical and/or experimental evidence.

Parameter	Description	Effect(s) on disease heterogeneity	Clinical and/or experimental evidence
*K*_1_	IC50 of AVPs on the viruses	With *K*_1_ increasing, *R*_IC_ develops potentially into asymptomatic state and *T*_IP_ could be prolonged	Asymptomatic infection is related to SARS-CoV-2 11083G>T mutation enhancing viral inhibitory effects on the antiviral state of the host [14, 45]
λ_2_	The activation rate of IFNs induced by viruses	Enhancement of λ_2_ prolongs *T*_IP_ and leads *R*_IC_ into asymptomatic state	Asymptomatic SARS-CoV-2 infected subjects display a very high serum type I IFN level [46]
*ρ*	The rate of T cell exhaustion	Increasing *ρ* leads to the switch of *R*_IC_ from mild-moderate into severe state; *T*_IP_ has no association with *ρ*	Elevated exhaustion level of T cells is present in severe patients [47]
*K*_4_	EC50 of cytokines inducing T cell exhaustion	Decreasing *K*_4_ promotes the switch of *R*_IC_ from mild-moderate into severe state	The cytokine storm may be responsible for increased PD-L1 expression (responding to decreased *K*_4_ in our work), leading to CD8^+^ T cell exhaustion [51]
[T_0_]	Density of naïve T cells	Decreased [T_0_] results in *R*_IC_ developing into severe state and could shortens *T*_IP_	Scarcity of naive T cells may be linked risk factors for severe COVID-19 [38]

**Note**: In our model, *R*_IC_ is the ratio of infected to total cells and *T*_IP_ is the simulated incubation period.

### Progression dynamics of SARS-CoV-2 infection

**Fig 3:**
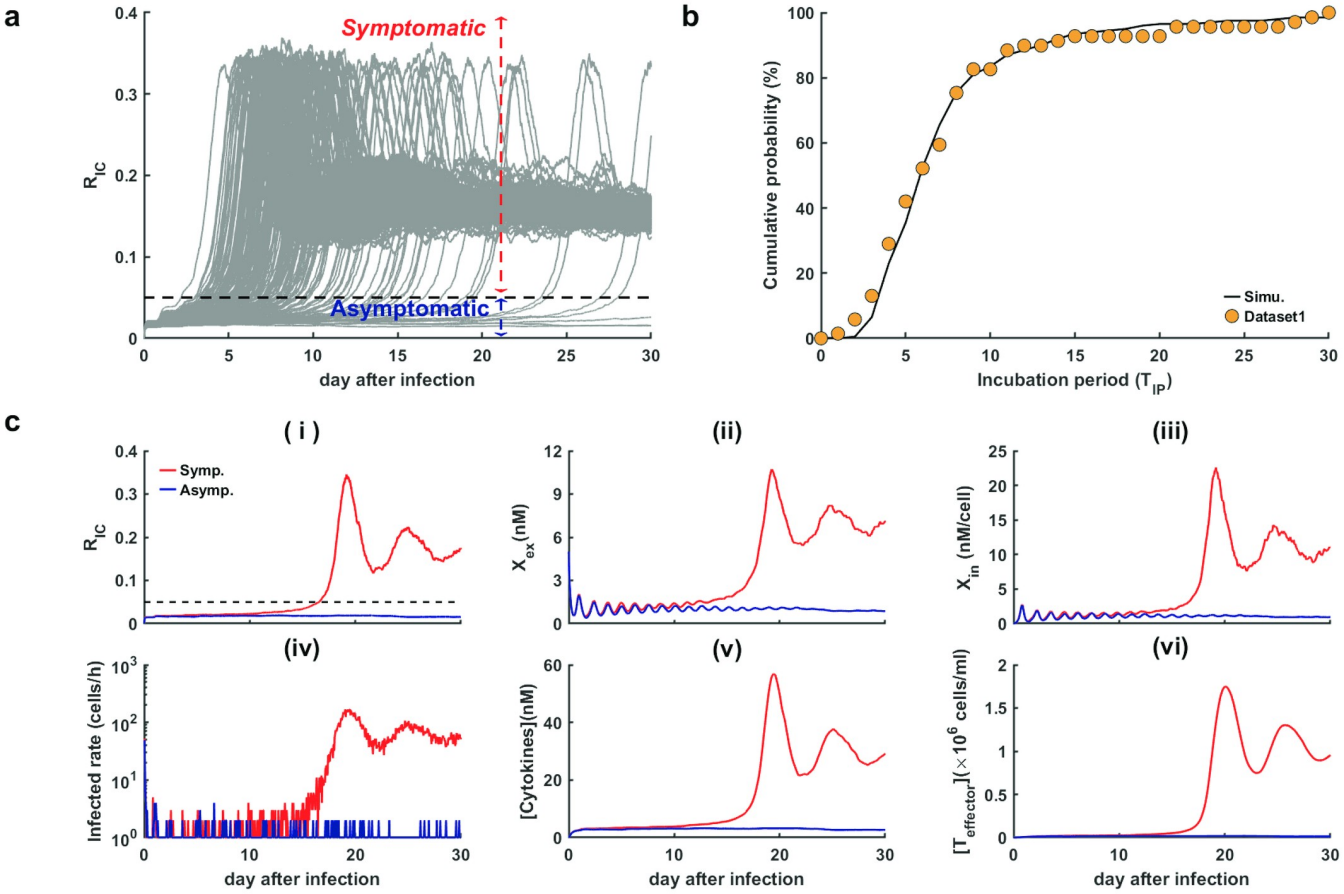
Progression dynamics in response to SARS-CoV-2 infection without considering T cell exhaustion (*ρ* = 0). **a**. Time course of the ratio of infected cells (*R*_IC_) (out of 200 independent runs). The black dashed line is the threshold between the asymptomatic and symptomatic state. **b**. Comparison of incubation periods between simulations and real data in COVID-19. The black line shows the cumulative probability obtained from default values in Table A in S1 Text (out of 200 independent runs). The orange dots represent real data of incubation periods from Dataset 1. **c**. Two simulated trajectories were developed for the symptomatic and asymptomatic states (red and blue lines, respectively). (i)-(vi) respectively for the ratio of infected cells (*R*_IC_), extracellular virus concentration (*X*_ex_), intracellular virus concentration per cell (*X*_in_), infected rate of susceptible cells, cytokine levels ([Cytokines]), and effector T cell counts ([*T*_effector_]). Other parameters were assigned default values shown in Table A in S1 Text.

To investigate the early stage dynamics of COVID-19 progression, we ran the model for 30 days after SARS-CoV-2 infection without considering of T cell exhaustion (*ρ* = 0) (Fig 3).

From numerical simulation, the ratio of infected cells (*R*_IC_) remained low for a few days after SARS-CoV-2 infection, and most cases switched to a higher ratio of about 0.1∼0.2 in 14 days. Nevertheless, there were also some cases that exhibited persistently low levels *R*_IC_ (*R*_IC_ < 0.05) even 30 days after infection (Fig 3a). These results suggest the existence of two subpopulations with markedly different dynamics of *R*_IC_, who are either symptomatic (*R*_IC_ ≥ 0.05) or asymptomatic (*R*_IC_ < 0.05). Hence, we introduced a threshold (*R*_IC_ = 0.05) to quantify the switch from symptomless to the state of presenting symptom after infection.

Clinically, COVID-19 patients exhibit incubation periods ranging from 2 to 14 days, with an average of 5–6 days, and rare patients present with longer periods of incubation greater than 14 days [31, 32]. Patients typically show no symptoms during the incubation period [30]. To verify the above threshold of *R*_IC_, we defined the simulated incubation period (*T*_IP_) of a patient as the time of *R*_IC_ increasing across the threshold value 0.05, i.e., $T_{\text{IP}}=\underset{t}{\text{arg}\;\text{max}}\left\{R_{\text{IC}}\left(t\right)\leq 0.05\right\}$. A simulation of 200 independent runs revealed good agreement between the cumulative probability of simulated incubation period and clinical data (Fig 3b). Hence, it is reasonable to distinguish between asymptomatic and symptomatic states using the threshold for the ratio of infected cells in the proposed model.

To further examine typical disease progression after SARS-CoV-2 infection, we selected two simulated trajectories developed for symptomatic and asymptomatic states, respectively, over 30 days (Fig 3c). During the early stage, both symptomatic and asymptomatic cases displayed similar viral dynamics, with low levels of *R*_IC_ and virus concentrations inside and outside the cells (Fig 3c(i)–3c(iii)). Next, the extracellular virus concentration began to increase in the symptomatic sample (Fig 3c(ii)), along with the increasing of cell infection rate (Fig 3c(iv)), and the increasing of *R*_IC_ (Fig 3c(i)). The infected cell numbers and virus concentrations spiked when *R*_IC_ increased across the threshold and developed to a stationary symptomatic state in later stages. In contrast, the asymptomatic sample presented persistent low levels of infected cells and virus concentrations the entire time (Fig 3c(i)–3c(iii)). The immune responses showed a similar process of viral dynamics in that both cytokines levels and effector T cell numbers were maintained at low levels during the early stage and spiked to high levels in the symptomatic sample when *R*_IC_ increased across the threshold (Fig 3c(v) and 3c(vi)). These results reveal different dynamics in patients with symptomatic and asymptomatic clinical manifestations. We note that the non-zero steady states of *R*_IC_ and *X*_ex_ are inconsistent with clinical observations that some patients can recovery without treatment (Fig 3c(i) and 3c(ii)). This is because there are some other immune mechanisms that lead to the viral clearance are not included in the model. Therefore, in our simulations, we can consider the patients as viral clearance when the values of *R*_IC_ and *X*_ex_ are low enough.

### Type I interferon modulates the transition from asymptomatic to symptomatic COVID-19

Type I interferon (IFN-I or IFN-*α*/*β*) is known to regulate patient response during the early stage after viral infection. Previous studies have shown that impaired interferon responses or inborn error type I IFN immunity may accelerate the clinical progression of patients infected with SARS-CoV-2 [33, 34]. To quantify the effects of type I IFN regulation, we varied the model parameters associated with viral replication (*K*_1_) and the interferon activation (λ_2_) to explore their effects on the switch from an asymptomatic to symptomatic state. The parameters *K*_1_ and λ_2_ associate with viral proteins, such as non-structure protein 6 (NSP6), which limits type I IFN synthesis and inhibits the establishment of an antiviral state based on the COVID-19 signaling pathways derived Kyoto Encyclopedia of Genes and Genomes (KEGG) [14]. In addition, sensitivity analysis showed that changes in *K*_1_ and λ_2_ were sensitive for the ratio of infected cells on day 30 after infection (Fig D and Section 3 in S1 Text for the detailed description).

**Fig 4:**
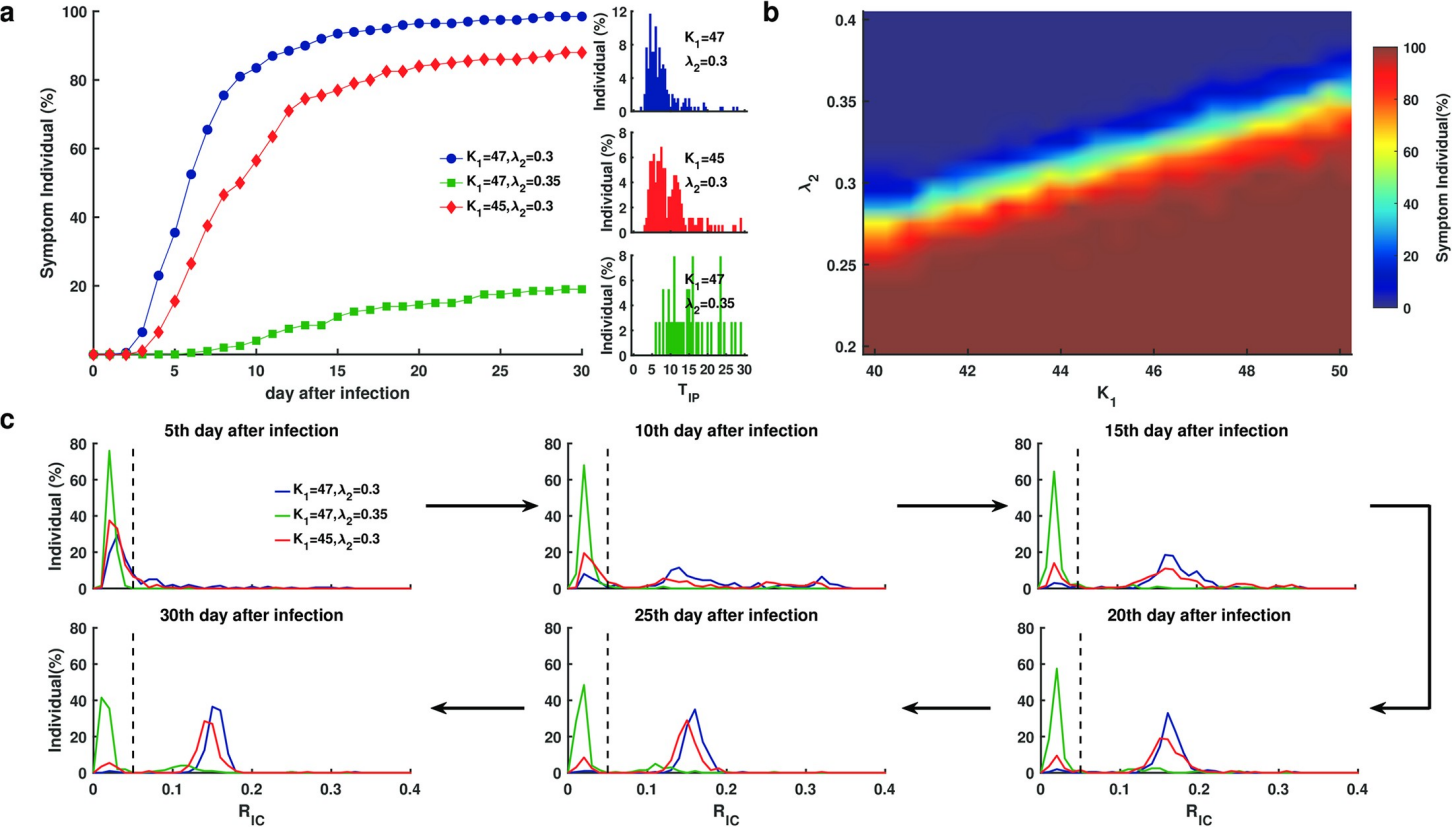
Disease evolution dynamics from asymptomatic and symptomatic states in response to SARS-CoV-2 infection without considering T cell exhaustion (*ρ* = 0). **a**. The percentage of cases (out of 200 independent runs) that developed into a state and the distribution of incubation period (*T*_IP_) for various IC50 values of viral replication (*K*_1_) and IFN response rate (λ_2_) after infection. **b**. The distribution of symptomatic frequency. The color column indicates the percentage of symptomatic cases (out of 100 individual runs) when *K*_1_ varies 40–50 and λ_2_ varies 0.2–0.4. **c**. The distribution (fraction of individual) of *R*_IC_ at six different time points. The black dashed lines show the threshold separating asymptomatic and symptomatic states. Different color lines correspond to the IC50 of viral replication (*K*_1_) and interferon response rate (λ_2_). Other parameters were given default values as shown in Table A in S1 Text.

To determine the influence of the IFN response preceding T cell exhaustion, we set *ρ* = 0, altered the parameters *K*_1_ and λ_2_, and performed 200 independent runs for each set of parameter values. First, we decreased *K*_1_ or increased λ_2_, respectively, according to the default values in Table A in S1 Text. For each simulated case, we examined the symptoms on day 30 and the incubation period (*T*_IP_) (Fig 4a). When *K*_1_ and λ_2_ took default values (*K*_1_ = 47, λ_2_ = 0.3), more than 50% of simulated cases were symptomatic on day 5 after infection, and most cases developed into a symptomatic state on day 15 (Fig 4a, blue). When *K*_1_ decreased (*K*_1_ = 45), some simulated cases remained asymptomatic on day 30 after infection, and most of incubation periods for the symptomatic cases were prolonged in the range from 10 to 15 days, while a few were even longer than 25 days (Fig 4a, red). When λ_2_ increased (λ_2_ = 0.35), less than 20% of simulated cases were symptomatic at day 30, and the majority of incubation periods for the symptomatic cases were greater than over 15 days (Fig 4a, green). We further examined the simulated results with *K*_1_ and λ_2_ varied over a parameter range 40 ≤ *K*_1_ ≤ 50, 0.2 ≤ λ_2_ ≤ 0.4. The parameter range revealed three well separated domains according to symptoms on day 30, a symptomatic domain in which most patients developed into symptomatic (Fig 4b, red), an asymptomatic domain in which most patients remained asymptomatic on day 30 after infection (Fig 4b, blue), and a bimodal region in which patients may show diverse transition dynamics from asymptomatic to symptomatic (Fig 4b). These results suggest that the IFN response is significant for the symptoms and incubation period of COVID-19 patients at early stages preceding T cell exhaustion.

To further explore the transition dynamics from asymptomatic to symptomatic states, we calculated the distribution of *R*_IC_ for all individuals on different days after infection. When the parameters were taken from the symptomatic region (*K*_1_ = 47, λ_2_ = 0.3) or the asymptomatic region (*K*_1_ = 47, λ_2_ = 0.35), most cases switched from *R*_IC_ < 0.05 to *R*_IC_ ≥ 0.05 within 20 days (Fig 4c, blue) or remained *R*_IC_ < 0.05 on day 30 (Fig 4c, green). Nevertheless, when the parameters were taken from the bimodal region, the transition dynamics could be diverse, and the ratio *R*_IC_ exhibited an obvious bimodal distribution from days 10 to 30 after infection (Fig 4c, red). These results indicate different transition dynamics in patients with various IFN response. Except intracellular IFN-I response, we further explored the influence of intercellular T cell response on asymptomatic patient and incubation period. The result exhibited that increased number of naive T cells significantly prolonged the incubation period and raised the proportion of asymptomatic cases, while the incubation period and the proportion of asymptomatic cases were insensitive with T cell exhaustion (Fig E and Section 8 in S1 Text for the detailed description).

### Characteristics of mild-moderate to severe symptoms in COVID-19 patients

COVID-19 patients exhibited distinct clinical manifestations in that 80% patients had only slight or mild symptoms, and some of them recovered by themselves, while 20% patients may further develop into severe situations where ventilators are required for survival [31, 35]. To explore the transition dynamics from mild-moderate to severe symptoms, we introduced the effect of T cells by setting *ρ* > 0, and varied the parameter *K*_4_ that quantifies the exhaustion level of T cells.

**Fig 5:**
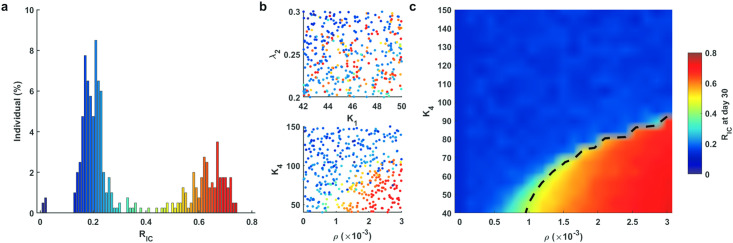
Bimorphism of patient symptoms. **a**. Distribution of *R*_IC_ on day 30 after SARS-CoV-2 infection. **b**. Scatterplot of varied parameters and *R*_IC_ on day 30. **c**. Distribution of the severity of COVID-19 patients. The color column shows *R*_IC_ at day 30 when *K*_4_ varies between 40 and 150 and *ρ* varies within [0, 3 × 10^−3^]. The black dashed line shows the threshold between mild-moderate and severe cases. Other parameters assigned the default values shown in Table A in S1 Text.

We performed numerical simulation for 400 independent runs using the randomly selected parameters 42 ≤ *K*_1_ ≤ 50, 0.2 ≤ λ_2_ ≤ 0.3, 0 ≤ *ρ* ≤ 3 × 10^−3^, and 40 ≤ *K*_4_ ≤ 150. The ratio of infected cells *R*_IC_ on day 30 for all runs exhibited an obvious bimodal distribution, corresponding to a high level ratio (0.4 < *R*_IC_ < 0.8) and a low level ratio (0.05 < *R*_IC_ ≤ 0.4), respectively (we also noted a few cases with *R*_IC_ ≤ 0.05, which corresponds to asymptomatic cases) (Fig 5a). These results suggest well defined mild-moderate and severe symptoms characterized by the ratio of infected cells, i.e., we refer to mild-moderate cases as those with 0.05 < *R*_IC_ ≤ 0.4, and severe cases as those with 0.4 < *R*_IC_ < 0.8.

To investigate the key parameters that are significant for patient symptoms, we showed scatter plots for each case according to the parameter values and *R*_IC_ on day 30 (Fig 5b). Parameters values for both mild-moderate and severe cases were evenly distributed evenly in the (*K*_1_, λ_2_) plane, while they were well separated in the (*ρ*, *K*_4_) plane, and severe cases primarily showed larger exhaustion rates *ρ* and smaller coefficients *K*_4_. In the model, the parameters *ρ* and *K*_4_ represented the effects of T cell exhaustion in later stage after SARS-CoV-2 infection. These results suggested that T cell exhaustion plays essential roles in the transition between mild-moderate and severe cases, which is consistent with clinical studies showing that impaired exhaustion features in SARS-CoV-2-reactive CD8^+^ T cells exist in on severe COVID-19 patients [36]. Some studies have shown that an impaired IFN response characterizes in severe or life-threatening patients [9, 33], and deficient IFN production can lead to the exhaustion of T cells, as T cell proliferation or T cell egress from lymphoid organs can be inhibited by delayed IFN response [37].

**Fig 6:**
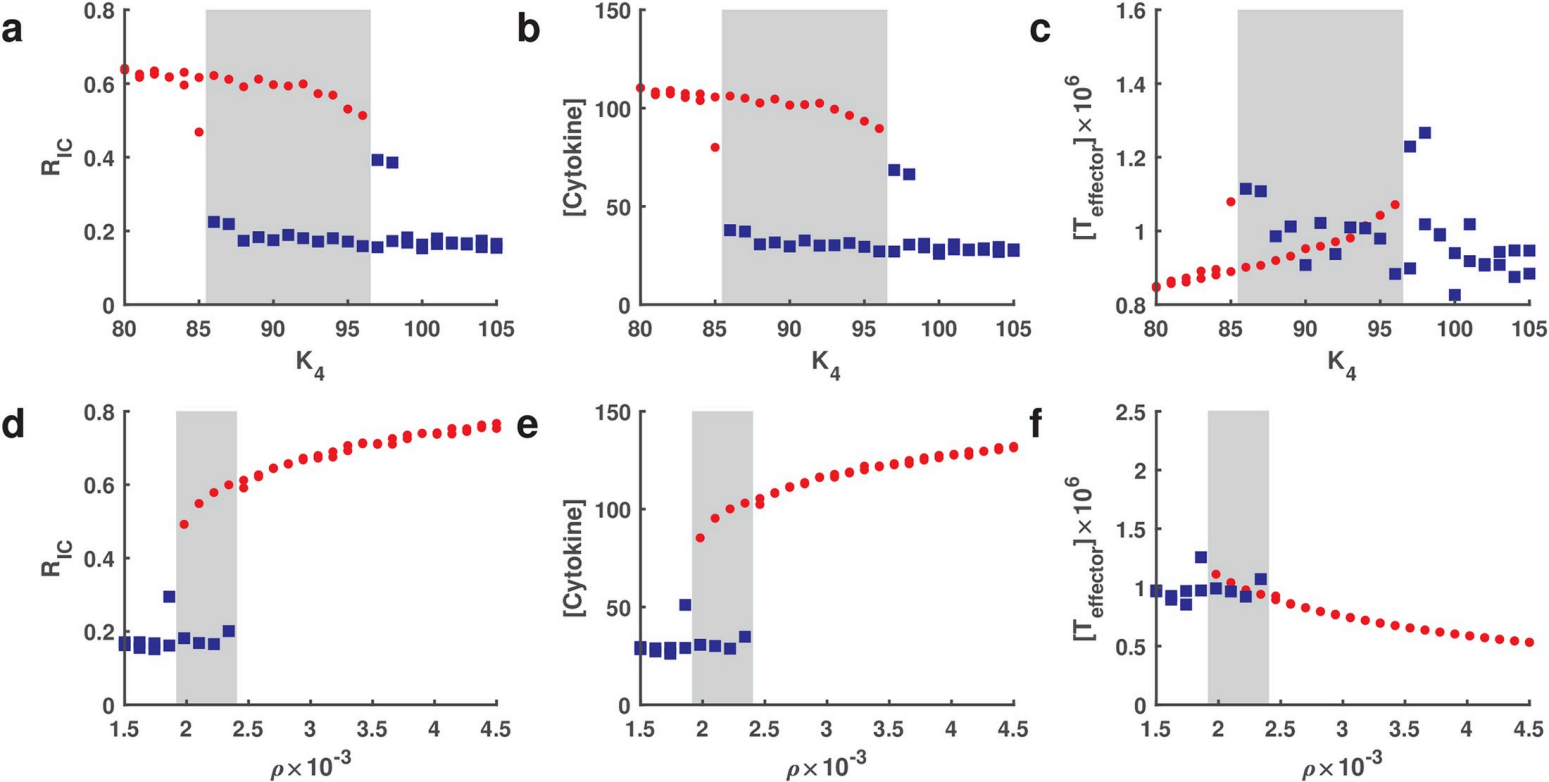
Bifurcation of parameter *K*_4_ and *ρ* for *R*_IC_, [Cytokines] and [*T*_effector_] on day 30 after infection. **a-c** for parameter *K*_4_ when the value of *ρ* is taken as 0.0025. **d-f** for parameter *ρ* when the value of *K*_4_ is taken as 84. Red solid circles and blue squares represent severe and mild-moderate cases, respectively. The other parameters are default and are shown in Table A in S1 Text. The gray region indicates a bistable status for mild-moderate and severe cases.

To further identify the parameter values that characterize mild-moderate and severe cases, we took parameters *ρ* and *K*_4_ over a range 0 ≤ *ρ* ≤ 3 × 10^−3^, 40 ≤ *K*_4_ ≤ 150, and fixed other parameters to their default values shown in Table A in S1 Text. The dependence of *R*_IC_ on the parameters *ρ* and *K*_4_ is shown in Fig 5c, illustrating good separation between mild-moderate and severe cases. We noted that the value of *R*_IC_ increased rapidly with increasing of *ρ* or decreasing of *K*_4_. We further performed a bifurcation analysis of parameters *ρ* and *K*_4_ related to T cell exhaustion (details in Section 6 in S1 Text). The result in Fig 6 demonstrated a bistable status for cytokines and *R*_IC_ in the range of 85 < *K*_4_ < 98 when *ρ* was taken as 0.0025 (Fig 6a and 6b). Similarly, a bistable region of *ρ* for cytokines and *R*_IC_ was in the range of 0.0018 < *ρ* < 0.0024 when *K*_4_ was taken as 84 (Fig 6d and 6e). These results imply that there is a risk of transition from mild-moderate to severe cases. Interestingly, the distribution of effector T cells was not similar to cytokines in the gray region (Fig 6c and 6f). In addition, we defined the first passage time from onset to severe cases and simulated the progression (details in Section 6 in S1 Text). The result indicated that the transition time decreases with increasing level of T cell exhaustion (*ρ*), while the transition time lengthens linearly when the level of anti-exhaustion of T cells (*K*_4_) increases (Fig F in S1 Text). These results could be of great assistance for clinical prognosis in predicting subsequent clinical course in COVID-19 patients.

### Activation and exhaustion of naïve T cells is related to the severity of COVID-19

Clinical studies have shown that age is associated with the development of severe COVID-19 [31], and further analysis of SARS-CoV-2-specific adaptive immune response during acute COVID-19 revealed that aging and scarcity of naïve T cells may be linked risk factors in severe patients [38]. To quantify the effect of naïve T cell scarcity, we varied the naïve T cells number [*T*_0_] in the model to explore the dynamic of COVID-19 progression.

**Fig 7:**
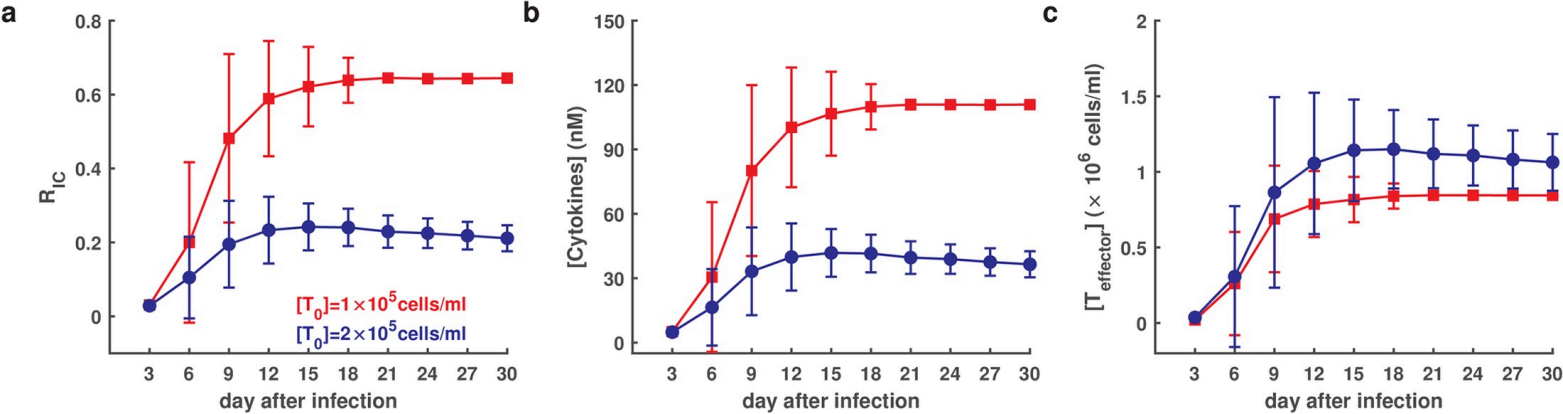
Dynamics of COVID-19 in response to a scarcity of naïve T cells and a mild exhaustion of T cells (*ρ* = 0.0005) (out of 100 individual runs). **a**. Time course of the average ratio of infected cells. **b**. Time course of average cytokines. **c**. Time course of average effector T cells. Red and blue lines indicate different levels of naïve T cells with ([*T*_0_] = 10^5^ cells/ml) and ([*T*_0_] = 2 × 10^5^ cells/ml), respectively. The error bar indicates standard deviation. Other parameters were assigned default values as shown in Table A in S1 Text.

In simulations, we set [*T*_0_] = 2 × 10^5^ cells/ml for a normal person, and [*T*_0_] = 1 × 10^5^ cells/ml for a scarcity of naïve T cells, and the mild T cell exhaustion rate *ρ* = 0.0005, and performed 100 independent runs. The normal cases developed mild-moderate symptoms mild-moderate symptoms (0.05 < *R*_IC_ < 0.4), while naïve T cell scarcity cases developed into severe symptoms (*R*_IC_ > 0.4) (Fig 7a). Moreover, naïve T cell scarcity cases exhibited magnification of cytokine levels (Fig 7b) and reduced effector T cells (Fig 7c) compared to normal cases. These results indicate that a scarcity of naïve T cells may potentially lead to cytokine ectopic secretion and T cell reduction, which is supported by observations that decreased naïve T cell production with aging may result in an inappropriate inflammatory response, increasing the likelihood of a cytokine storm [39].

### Treatment efficacy and prognosis of COVID-19 patients

The above numerical simulations reproduced the disease progression in patients infected with SARS-CoV-2. We further applied the model to evaluate the efficacy of different treatment methods, acceleration of IFN response (*ε*_1_), restriction of viral replication (*ε*_2_), promotion of extracellular virus clearance (*ε*_3_), and inhibition of T cell exhaustion (*ε*_4_) (details in Methods). We let a quadruple (*ε*_1_, *ε*_2_, *ε*_3_, *ε*_4_) represents a combination of the four types of treatments, and the quadruple (0, 0, 0, 0) indicates the control cases with no treatment. The efficacy of a treatment strategy was quantified by the relative reduction of the ratio of infected cells on day 30 after infection (clinical therapy starts on day 15), which is formulated as

$$\begin{array}{c} E\left(\varepsilon_{1},\varepsilon_{2},\varepsilon_{3},\varepsilon_{4}\right)=\frac{R_{\text{IC}}^{30}\left(0,0,0,0\right)-R_{\text{IC}}^{30}\left(\varepsilon_{1},\varepsilon_{2},\varepsilon_{3},\varepsilon_{4}\right)}{R_{\text{IC}}^{30}\left(0,0,0,0\right)} \end{array}$$

where $R_{\text{IC}}^{30}\left(\varepsilon_{1},\varepsilon_{2},\varepsilon_{3},\varepsilon_{4}\right)$ represents the ratio of infected cells on day 30 after infection when treated with strategy (*ε*_1_, *ε*_2_, *ε*_3_, *ε*_4_). The efficacy takes a value from 0 to 1, and larger values indicate increased efficiency of the treatment strategy.

**Fig 8:**
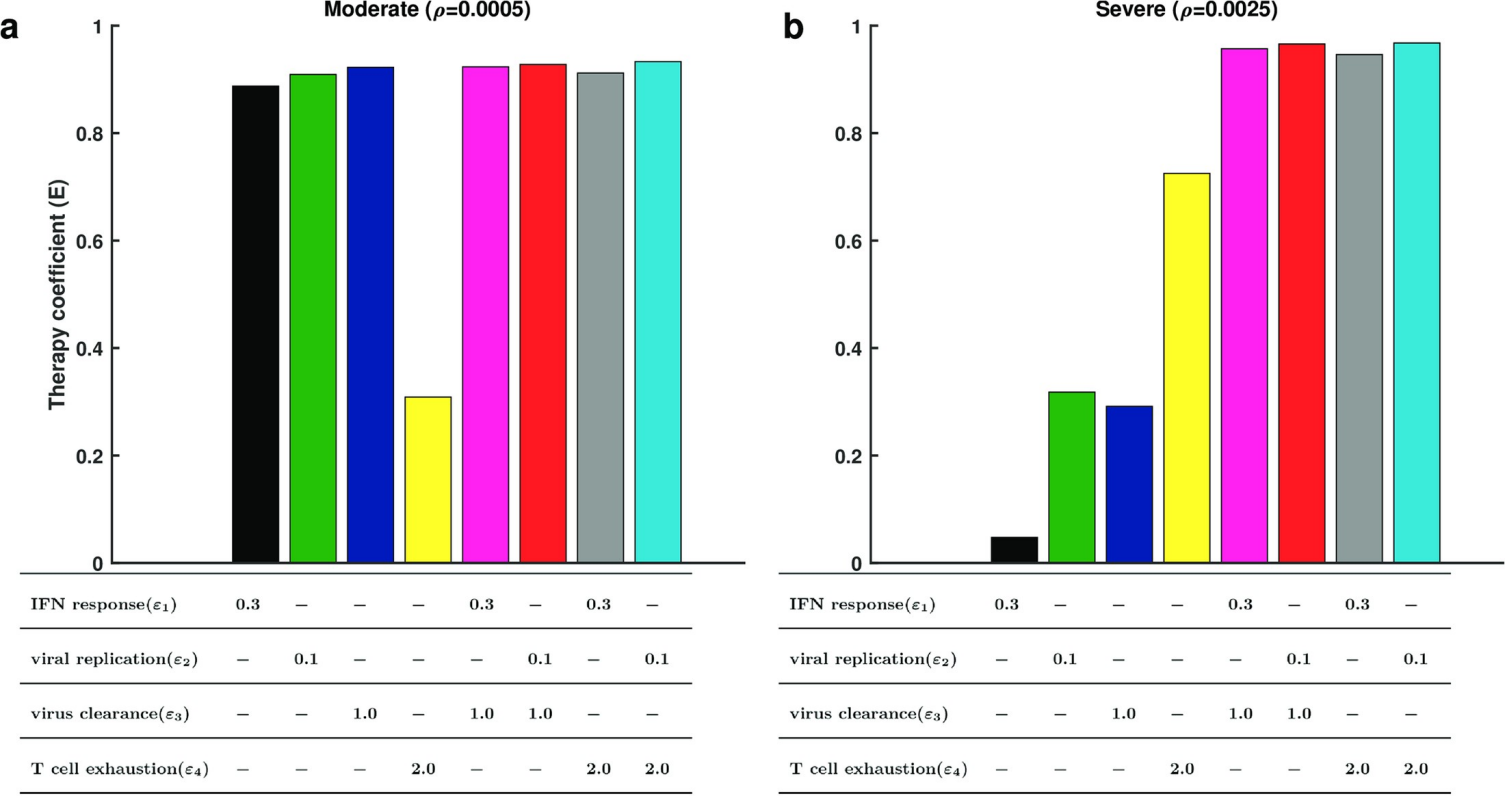
Comparison of the simulated efficacy for different treatment strategies in moderate and severe cases. **a** and **b** are the simulated efficacy of eight treatment strategies for moderate patients (*ρ* = 0.0005) and severe patients (*ρ* = 0.0025), respectively. Different colors correspond to treatment strategies. The table below the histogram shows the detailed values of the quadruple (*ε*_1_, *ε*_2_, *ε*_3_, *ε*_4_). “-” indicates that corresponding values are 0. Other parameters were given default values as shown in Table A in S1 Text.

To compare the efficacy in patients with moderate or severe symptoms, we solved the model using different levels of T cell exhaustion in moderate (*ρ* = 0.0005) and severe (*ρ* = 0.0025) patients. For moderate patients, a single treatment of accelerating IFN response, restricting viral replication, or promoting viral clearance was highly efficient (*E* > 0.8), but inhibition of T cell exhaustion alone exerted only marginal efficacy (*E* ≈ 0.3) (Fig 8a), and the density of both cytokines and effector T cells were low (Fig G and Section 7.1 in S1 Text for the detailed description). For severe patients, however, inhibition of T cell exhaustion alone resulted in an efficacy of (*E* > 0.6) and a decreased concentration of cytokines (Fig Ga in S1 Text), while the other three methods alone yielded low efficacy (*E* < 0.4) (Fig 8b) and the density of neither cytokines nor effectors was changed (Fig Gb). In addition, we ran further simulations for drug efficacy using dose response curves (Fig H and Section 7.2 in S1 Text for the detailed description). The results indicated that the maximum efficacy of targeting IFN response (*ε*_1_), viral replication (*ε*_2_), and virus clearance (*ε*_3_) was close to 1 (Fig Ha-c, He-g and Table B in S1 Text), but the maximum efficacy of targeting T cell exhaustion (*ε*_4_) only reached 0.8 (Fig Hh and Table B in S1 Text) for severe case and was even less at 0.3 in moderate cases (Fig Hd and Table B in S1 Text), suggesting that antiviral treatment is more effective than immunological treatment for both moderate and severe cases. We fitted the dose response curves with Hill funcitons, and compared the Hill coefficient n and EC50 of the functions for moderate and severe cases in response to the same treatment strategy (Table B and Section 7.2 in S1 Text for the detailed description). The coefficient n in moderate cases was generally smaller than in severe cases, and the value of EC50 in severe cases was generally larger than in moderate cases, implying that moderate cases are more sensitive to treatment than severe cases, and higher doses are required for severe cases. These results suggest that antiviral treatment should be recommended for moderate patients, while for severe patients, inhibition of T cell exhaustion should be considered. Our results are in agreement with opinions that immune checkpoint inhibitors (ICIs) should be applied to treat severe COVID-19 patients [40, 41].

**Fig 9:**
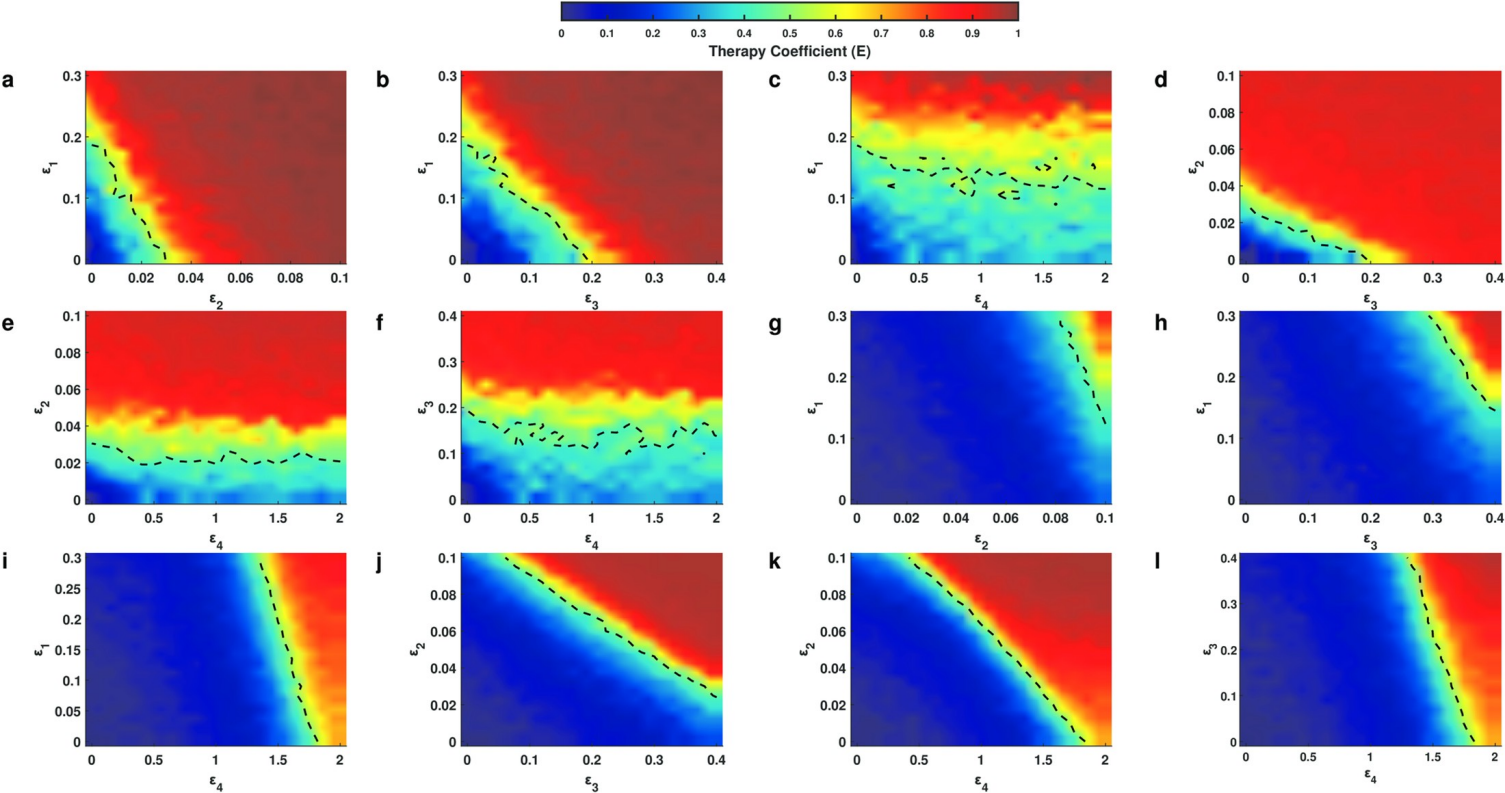
Therapeutic efficacy of united treatments for moderate and severe cases. **a-f** are combination of treatments for moderate cases. **g-l** are combination of treatments for severe cases. The dotted lines indicate half maximal efficacy. The colored bar is the therapeutic coefficient.

Moreover, for both moderate and severe patients, the combination of antiviral treatments (acceleration of IFN response, restriction of viral replication or promotion of virus clearance) and immune therapy (inhibition of T cell exhaustion) yielded highly efficiency for improving patient symptoms (*E* > 0.8) (Fig 8), and the density of both cytokines and effector T cells were low (Fig G in S1 Text). To quantify the relationship between therapeutic efficacy and combination therapy, we simulated different combinations in heterogeneous patients (Fig 9). On the one hand, the result of combination antiviral treatments (Fig 9a, 9b and 9d) demonstrated that the antiviral drugs could take less dose than EC50 (Table B in S1 Text) while maintaining high efficacy for moderate cases. The results from treatments for severe cases (Fig 9g, 9h and 9j) were similar to moderate cases, and the dose of antiviral treatment referred to the EC50 of a single antiviral treatment for moderate patients. These results suggest that a combination of treatments decreases drug dose while maintaining high efficacy for the treatment of COVID-19. On the other hand, the immunological treatment combined with any antiviral treatment did not result obvious improvements compared to the single antiviral treatment in moderate cases (Fig 9c, 9e and 9f), but combination of the EC50 of *ε*_4_ in severe cases and low doses of other antiviral drugs maintained high levels of treatment efficacy in severe cases(Fig 9h, 9k and 9l), suggesting that the combination of immunological and antiviral treatments should be applied to treat severe cases.

Clinically, type I interferon treatment is a method of antiviral treatment for pathogenic human coronavirus infections [42], and medical use of Arbidol, an antiviral treatment, improves viral clearance and clinical outcomes in COVID-19 patients [43, 44]. To investigate the dynamics of antiviral treatment, we simulated treatment dynamics by accelerating of IFN response (*ε*_1_ = 0.1) and promoting of extracellular virus clearance (*ε*_3_ = 1.0) for both moderate (*ρ* = 0.0005) and severe (*ρ* = 0.0025) cases. For single drug treatment, the ratio of infected cells for moderate cases rapidly decreased at the beginning of treatment and reached less than 0.05 (the threshold of the asymptomatic and symptomatic states) within 5 days after treatment (Fig Ia in S1 Text), however, for severe cases, the ratio of infected cells reduced more slowly and maintained the severe case (0.4 < *R*_IC_ < 0.8) on day 15 after treatment (Fig Ib in S1 Text). When combination of the two treatment strategies was applied, the ratio of infected cells in both moderate and severe cases reached less than 0.05 within 10 days after treatment (Fig I in S1 Text). These results illustrate the different dynamics of drug treatment for moderate and severe patients.

## Discussion

Patients with COVID-19 exhibit marked individual heterogeneity in their disease progression. Quantitatively understanding how interactions between viral dynamics and host immune responses affect disease progression is important for clinical diagnosis and treatment. In this study, we developed a multi-scale mathematical model of the dynamics of SARS-CoV-2 infection. The model was established to describe the major biological processes associated with viral dynamics and host immune response, as well as the dynamics of infected cell populations in a viral microenvironment with respect to viral infection, viral replication, IFN response, viral budding, and immune clearance that appear in single cells (Fig 2 Intracellular). This model incorporates cross-talk among viral dynamics, cytokines and T cell responses with respect to the progression of COVID-19 (Fig 2 Intercellular). Multiscale simulations allowed us to quantify the heterogeneity of IFN responses (Fig 4) and T cell responses (Figs 5–7) that may result in the different severities of COVID-19. Heterogeneity plays an important role in the evolution of COVID-19 and lead to diverse disease progression in patients. The proposed model provides a method to quantify the therapeutic effects of potential treatment strategies in COVID-19 patients with different disease severities.

Numerical simulations demonstrate that IFN response is essential to modulate the transition from asymptomatic to symptomatic presentation and prolongs the incubation period (Fig 4a). We observed a diverse distribution of symptom presentation for heterogeneous IFN response from patients with COVID-19 (Fig 4b). Based on statistical analysis of genomic data from COVID-19 patients, asymptomatic infection is related to SARS-CoV-2 11083G>T mutation at residue 37 of non-structure protein 6 (NSP6) [45]. This mutation enhances viral stimulation of interferon and the expansion of viral inhibitory effects on the antiviral state of the host [14]. Quantitative study confirmed our simulation that patients with high levels of IFN response usually have asymptomatic manifestations [46]. These results can guide the disease management of patients in accordance with their responses in early stages after SARS-CoV-2 infections.

Many patients with COVID-19 appear to transition from mild-moderate to severe symptom in a short time, similar to a dynamic process of toggle switches between the two states. Clinical data have indicated significant differences in serum cytokines and active T cells in patients with mild-moderate and severe symptoms. Numerical simulations based on the proposed model revealed a bimorphism of symptoms that correspond to distinguished symptom manifestations of either a mild-moderate or severe state (Fig 5a). The transitions between mild-moderate and severe manifestation were closely associated with the model parameters quantifying the exhaustion of T cells (Figs 5 and 6 and Fig F in S1 Text). Clinically, elevated exhaustion level of T cells was present in severe patients [47], which is consistent with our numerical results. In addition, we simulated the infection dynamics under a scarcity of naïve T cells, and found that a scarcity of naïve T cells leads to the severe state with high levels of cytokines and a reduction in effector T cells (Fig 7). The simulation results indicate a disruption in the balance between the exhaustion of T cells and cytokine production in restricting virus spreading. Under normal conditions, cytokines produced from both infected cells and T cells promote the generation of effector T cells from naïve cells. Moreover, increased of cytokines induces the exhaustion of T cells [48] to maintain a balance between cytokine secrete and T cell activation. Nevertheless, this balance can be broken due to a scarcity of naïve T cells, which leads to severe and even life-threatening cases.

Based on the disease progression obtained from our model, we suggest potential methods for treating patients with different symptoms. Single antiviral treatment is effective for patients with moderate symptoms, while immunotherapy and combination treatment should be considered for severe patients (Fig 8 and Figs G and H in S1 Text). The results of quantitative treatment for COVID-19 (Fig 9) suggest that a combined immunotherapy with antiviral drug could be a potential strategy for COVID-19 severe patients. The timing of drug administration is certainly important for the overall effects of combination therapy, and the problem of optimal drug administration protocol is beyond the current study. Clinically, most moderate patients are treated with antivirals, while many patients with severe symptom presentation are treated with combined treatment [49, 50], which in agreement with our treatment strategies for COVID-19 patients. We further modeled trajectories of two antiviral treatments, type I interferon and Arbidol [44], for moderate and severe patients (Fig I in S1 Text). Although it is difficult to make precise predictions in the absence of clinical trial data, our results highlight the efficacy of single antiviral treatment for moderate patients and the necessity of combination treatments for severe patients.

There have been many published and preprint reports of predictive mathematical models for the COVID-19 pandemic. These epidemiological models can be valuable for the prediction and controlling of disease spreading [52, 53]. Pharmacokinetic model is also applied to quantitatively predict treatment of drugs for COVID-19 [54]. Less attention has been paid to the predictive models of disease progression in heterogeneity outcome. Recently, a mechanistic, within-host ODE model was established to study the immune response to SARS-CoV-2 and the impact of delayed IFN on infection dynamics [55]. Virtual patient cohorts were generated based on an algorithm of random parameter sampling, and dynamics of how immune mechanisms drive disease outcomes was discussed. In our study, the stochastic and multiscale model was developed to consider the inherent heterogeneity of the infection process and the related clinical therapy dynamics. The multi-scale mathematical model proposed in this study was intended to establish a predictive model for disease progression from viral infection to patient symptoms and to provide quantitative understanding of the heterogeneous clinical courses in patients with COVID-19. For example, the process of IFN response and T cell response modulate the evolution of COVID-19 stage, from which potential clinical methods are suggested based on model simulations. The proposed model primarily incorporates viral dynamics with host immune response. Further interactions between cytokines and immune cells were omitted in the current model, which are important for the understanding of the molecular details of T cell exhaustion and the cytokine storm that are crucial for severe patients and the cause of death from COVID-19 [24, 25]. Extensions of the current model to include these details are certainly required, and challenging, for a better understanding of the disease progression, especially the prediction of clinical course and early warning of a COVID-19-induced cytokine storm. In addition, the proposed model framework can also be applied to study other coronaviruses as long as there is available data.

## Methods

### Collected data from the published literatures

**Dataset 1: Clinical data on the incubation period of 69 COVID-19 patients from China**. The data were collected from the literature [56, 57].

**Dataset 2: Clinical data of 41 COVID-19 patients from the Fifth Medical Center of PLA General Hospital in Beijing, China**. The data were retrieved from the reference, which includes expression of exhaustion biomarkers, cytokines and T cell counts in 6 healthy donors, 29 mild and 12 severe patients [58].

**Dataset 3: Routine blood data from 107 severe patients including 58 survivors and 49 deaths after clinical treatment at the Renmin Hospital of Wuhan University, China**. The data includes cytokines levels from 58 survivors and 49 deaths. All confirmed COVID-19 patients were severe before treatment [59].

**Dataset 4: Clinical data from 50 healthy donors and 157 COVID-19 patients including 117 moderate and 40 severe symptoms, were collected from Yale New Haven Hospital, United States**. The data from 117 moderate cases and 40 severe cases includes cytokines, CD4^+^ T cell and CD8^+^ T cell count, and the percentage of naïve CD4^+^ and CD8^+^ T cells [60].

**Dataset 5: Single cell sequence data from 8 patients as moderate and 13 patients as critical from Charité-Universitätsmedizin Berlin and University Hospital Leipzig**. Genomic data of immune cells or epithelial cells from 8 patients as moderate and 13 patients as critical are included in the dataset [61].

**Dataset 6: Proteomic data from 46 COVID-19 and 53 control individuals from Taizhou Hospital, China**. Proteomic data of receptor proteins for SARS-CoV-2 are incorporated into the dataset [62]. We only found the data for neuropilin-1 (NRP1) that is one of the receptors for SARS-CoV-2 [63] so that receptor protein was approximately equivalent to NRP1.

**Dataset 7: Kinetic data of effector T cells from 707 COVID-19 patients from Tongji Hospital, Wuhan, China**. This dataset involves T cells dynamics from patients with different symptom presentations such as moderate (410 cases), severe (206 cases) and critical (91 cases) in hospitalized patients [64].

Dataset 1 was used to validate the definition of incubation period in our model. The model assumptions stemmed from data analysis to datasets 2, 3, 4 and 5. We estimated the distribution of receptor protein from dataset 6 and the part of the parameters in our model from dataset 7 (Details in S1 Text).

### Mathematical formulation of the multi-scale model

The multi-scale model describes the evolution of viral dynamics and host immune response in respone to SARS-CoV-2 infection. The model was formulated using a set of differential equations for the intracellular virus RNA concentration $X_{\text{in}}^{i}$, interferons concentration [IFNs]^*i*^, antiviral protein [AVPs]^*i*^(*i* = 1, 2, ⋯, *N*), extracellular virus RNA concentration *X*_ex_, cell surface free receptor protein number *R*^*i*^(*i* = 1, 2, ⋯, *N*), cytokine concentration [Cytokines], and the effector T cell density [*T*_effector_]. The model equations are detailed below.

#### Viral dynamics

We assumed that each receptor protein on target cell surface can only bind to one spike protein of SARS-CoV-2, and the receptor protein is freed when SARS-CoV-2 releases its RNA into the host cell and the spike protein dissociates from the receptor protein. These processes give a flux $v_{1}^{i}$ as

$$\begin{array}{c} v_{1}^{i}=k_{\text{on}}\left(R_{0}^{i}-R^{i}\right)X_{\text{ex}}-k_{\text{off}}R^{i} \end{array}$$ (1)

Here, *k*_on_ is the binding rate between SARS-CoV-2 and receptor, *k*_off_ is the dissociation rate between SARS-CoV-2 and receptor, $R_{0}^{i}$ represents the total number of receptor protein on the ith cell, and *R*^*i*^ represents the receptor proteins binding to SARS-CoV-2. We assumed that the total number of receptor proteins on each cell is a constant over time, however the number is variable for different cells, and obeys gamma distribution with parameters *α*_1_ and *α*_2_, i.e., $R_{0}^{i}∼\Gamma \left(\alpha_{1},\alpha_{2}\right)$ (details shown in Table A in S1 Text).

SARS-CoV-2 release its RNA to the host cell after the spike protein is bound to the receptor on the target cell. The influx ($v_{2}^{i}$) of RNA to the host cell is proportional to the bound receptor protein, and hence

$$\begin{array}{c} v_{2}^{i}=k_{\text{in}}R^{i} \end{array}$$ (2)

The receptor becomes free when the genomic RNA is released.

Inside infected cells, the RNA of SARS-CoV-2 directs RNA replication and viral assembly using organelles and synthases from the host cell. During the process of viral replication, interferon signaling pathway is activated to produce interferons (IFNs) and antiviral proteins (AVPs), resulting in limited viral replication. In our model, we assumed that there is a time delay in the processes of virus replication (*τ*_1_) because the process includes multistep reactions, and the inhibition of viral replication is described by a Hill type function. Therefore, the influx of viral replication ($v_{3}^{i}$) is described as below:

$$\begin{array}{c} v_{3}^{i}=\lambda_{1}X_{\text{in}}^{i}\left(t-\tau_{1}\right)\frac{b_{1}K_{1}^{m_{1}}}{K_{1}^{m_{1}}+{\left({\left[\text{AVPs}\right]}^{i}\right)}^{m_{1}}}-\delta_{1}X_{\text{in}}^{i} \end{array}$$ (3)

Here λ_1_, *τ*_1_, *b*_1_, *m*_1_, *δ*_1_ are constants (details shown in Table A in S1 Text).

With respect to interferons (IFNs), they are activated by viral RNA and exhibit positive autoregulation [29]. So, the influx of IFNs pure synthesis is

$$\begin{array}{c} v_{4}^{i}=\lambda_{2}X_{\text{in}}^{i}+b_{2}\frac{{\left({\left[\text{IFNs}\right]}^{i}\right)}^{m_{2}}}{{\left({\left[\text{IFNs}\right]}^{i}\right)}^{m_{2}}+K_{2}^{m_{2}}}-\delta_{2}{\left[\text{IFNs}\right]}^{i} \end{array}$$ (4)

here *m*_2_, *K*_2_, *δ*_2_ are constants (details shown in Table A in S1 Text).

Antiviral proteins (AVPs) are synthesized by stimulated IFN downstream signal pathways and are degraded naturally in the host cell. Hence, the influx of AVPs pure synthesis is

$$\begin{array}{c} v_{5}^{i}=\lambda_{3}{\left[\text{IFNs}\right]}^{i}-\delta_{3}{\left[\text{AVPs}\right]}^{i} \end{array}$$ (5)

here λ_3_, *δ*_3_ are constants (details in Table A in S1 Text).

Finally, progeny virus is assembled by organelles and synthases from the host cell after viral replication. Since this process involves multistep reactions, we assumed a lag time *τ*_2_ for the process of viral budding, such that

$$\begin{array}{c} v_{6}^{i}=q_{0}X_{\text{in}}^{i}\left(t-\tau_{2}\right) \end{array}$$ (6)

here *q*_0_, *τ*_2_ are constants (details shown in Table A in S1 Text).

#### Multi-cellular responses and cell infection

All SARS-CoV-2 particles released from host cells enter the extracellular environment. The extracellular virus can either bind to receptor proteins in target cells and infect the host cells, or are cleared from the body. There are many different types of cells in the tissue environment, and these cells may affect the identification and binding of SARS-CoV-2 to target cells that express the receptor. Biologically, it is not a trivial process to initiate an invading process of a cell by viruses. Cellular self-defense in target cells protects the cells against pathogens [65], and viruses in a nearby cell can induce signals to promote the binding between viral spike proteins and cell membrane proteins. Here, we ignored the detail process and assumed a random process that extracellular viruses identify a target cell with a probability $\beta^{i}=\beta \left(X_{\text{ex}},R_{0}^{i},A\right)$ that depends on virus concentration outside the cell, cell membrane receptor protein number, and the binding affinity *A* = *k*_off_/*k*_on_. Hence, we write

$$\begin{array}{c} \beta \left(X_{\text{ex}},R_{0}^{i},A\right)=\beta_{0}\frac{{\left(X_{\text{ex}}R_{0}^{i}\right)}^{m_{0}}}{{\left(K_{0}\left(A+X_{\text{ex}}\right)\right)}^{m_{0}}+{\left(X_{\text{ex}}R_{0}^{i}\right)}^{m_{0}}} \end{array}$$ (7)

here, *K*_0_ is constant (details shown at Section 2.2 in S1 Text).

The binding affinity measures the equilibrium dissociation constant between the S protein on SARS-CoV-2 and the receptor protein on target cells. In our model, all cells are initially normal and uninfected, and once a cell is recognized by SARS-CoV-2 and the infection is initiated, the status of the cell becomes infected. Let *I*(*t*) ⊆ {1, 2, ⋯, *N*} the index of infected cells at time t. Let *v*_cell_ denote the volume of a single cell, and *V*_ex_ denote the volume of extracellular environment, the above biological processes lead to the following differential equations.

$$\begin{array}{c} \frac{dX_{\text{ex}}}{dt}=\frac{v_{\text{cell}}}{V_{\text{ex}}}\sum_{i\in I(t)}q_{0}X_{\text{in}}^{i}(t-\tau_{2})+\frac{1}{V_{\text{ex}}}\sum_{i\in I(t)}\left[k_{\text{off}}R^{i}-k_{\text{on}}\left(R_{0}^{i}-R^{i}\right)X_{\text{ex}}\right]-\delta_{4}X_{\text{ex}} \end{array}$$ (8)

$$\begin{array}{c} \frac{dR^{i}}{dt}=k_{\text{on}}\left(R_{0}^{i}-R^{i}\right)X_{\text{ex}}-k_{\text{off}}R^{i}-k_{\text{in}}R^{i} \end{array}$$ (9)

$$\begin{array}{c} \frac{dX_{\text{in}}^{i}}{dt}=\frac{k_{\text{in}}R^{i}}{v_{\text{cell}}}+\lambda_{1}X_{\text{in}}^{i}(t-\tau_{1})\frac{b_{1}K_{1}^{m_{1}}}{K_{1}^{m_{1}}+{\left({\left[\text{AVPs}\right]}^{i}\right)}^{m_{1}}}-\delta_{1}X_{\text{in}}^{i}-q_{0}X_{\text{in}}^{i}(t-\tau_{2}) \end{array}$$ (10)

$$\begin{array}{c} \frac{d{\left[\text{IFNs}\right]}^{i}}{dt}=\lambda_{2}X_{\text{in}}^{i}+b_{2}\frac{{\left({\left[\text{IFNs}\right]}^{i}\right)}^{m_{2}}}{{\left({\left[\text{IFNs}\right]}^{i}\right)}^{m_{2}}+K_{2}^{m_{2}}}-\delta_{2}{\left[\text{IFNs}\right]}^{i} \end{array}$$ (11)

$$\begin{array}{c} \frac{d{\left[\text{AVPs}\right]}^{i}}{dt}=\lambda_{3}{\left[\text{IFNs}\right]}^{i}-\delta_{3}{\left[\text{AVPs}\right]}^{i} \end{array}$$ (12)

Here *v*_ex_ = *V*_ex_/*N* denotes the mean volume that a single cell occupies the extracellular environment. From the above equations, the viral dynamics and cellular responses are coupled using the indexes *I*(*t*) of infected cells.

#### T cell response for clearing virus

Next, to model the T cell response to virus, we considered the cytokine concentration [Cytokines] and the effector cell number [*T*_effector_], and assumed a constant naïve T number [*T*_0_] over time. The infected cells secrete cytokines and activate the naïve T cells to produce effector T cells. The effector T cells continuously clear the infected cells and secrete cytokines, which may lead to chronic inflammation and further induce the exhaustion of effector cells. These processes are described by integrodifferential equations given below

$$\begin{array}{c} \frac{d[T_{\text{effector}}]}{dt}=\mu_{1}\left[T_{0}\right]\frac{{\left[\text{Cytokines}\right]}^{m_{3}}}{{\left[\text{Cytokines}\right]}^{m_{3}}+K_{3}^{m_{3}}}-\delta_{\text{effector}}\left(\left[\text{Cytokines}\right]\right)\left[T_{\text{effector}}\right] \end{array}$$ (13)

$$\begin{array}{c} \frac{d[\text{Cytokines}]}{dt}=\mu_{2}R_{\text{IC}}+\mu_{3}[T_{\text{effector}}]-\delta_{6}[\text{Cytokines}] \end{array}$$ (14)

Here, *R*_IC_ denotes the ratio of infected cells to total cells.

The coefficient *δ*_effector_ ([Cytokines]) represents the rate of effector T cell exhaustion/cleaning, which is dependent on the chronic inflammatory environment due to the accumulation of cytokines. Hence, we assumed that *δ*_effector_ ([Cytokines]) depends on the accumulation of a Hill type function of [Cytokines] over a period of effective time [66], which is formulated as

$$\begin{array}{c} \delta_{\text{effector}}\;([\text{Cytokines}])=\delta_{5}+\rho H(t-t_{2})\int_{t-\tau_{3}}^{t}\frac{{\left[\text{Cytokines}](s\right)}^{m_{4}}}{{\left[\text{Cytokines}](s\right)}^{m_{4}}+K_{4}^{m_{4}}}ds \end{array}$$ (15)

here *δ*_5_ is the basal cleaning rate, *ρ* is the maximum rate of T cell exhaustion, and *τ*_3_ is the effective period of cytokines. The Heaviside function *H*(*t* − *t*_2_) is introduced to represent the starting of T cell exhaustion, where

$$\begin{array}{c} H(t)=\{\begin{array}{ccc} 1 & & t>0\\ 0 & & t\leq 0 \end{array} \end{array}$$ (16)

Here *t*_2_ = *T*_IP_ to indicates the starting time of T cell exhaustion from a symptomatic state. Clinically, T cell exhaustion is associated with the expression of specific immune-inhibitory factors including PD-1 and Tim-3 on the cell surface [20, 58], but the origin of T cell exhaustion remains unclear [23, 28]. Here, we simply introduced variables *ρ* and *K*_4_ to model the effects of T cell exhaustion.

Finally, we assumed that infected cells are cleared by effector T cells and that the clearing rate is proportional to the effector T cell number, i.e., the clearing rate

$$\begin{array}{c} \eta (t)=\eta_{0}[T_{\text{effector}}] \end{array}$$ (17)

where *η*_0_ is a constant rate. When an infected cell is cleared, a normal cell is generated to maintain the total cell number *N*.

### Numerical scheme

**Fig 10:**
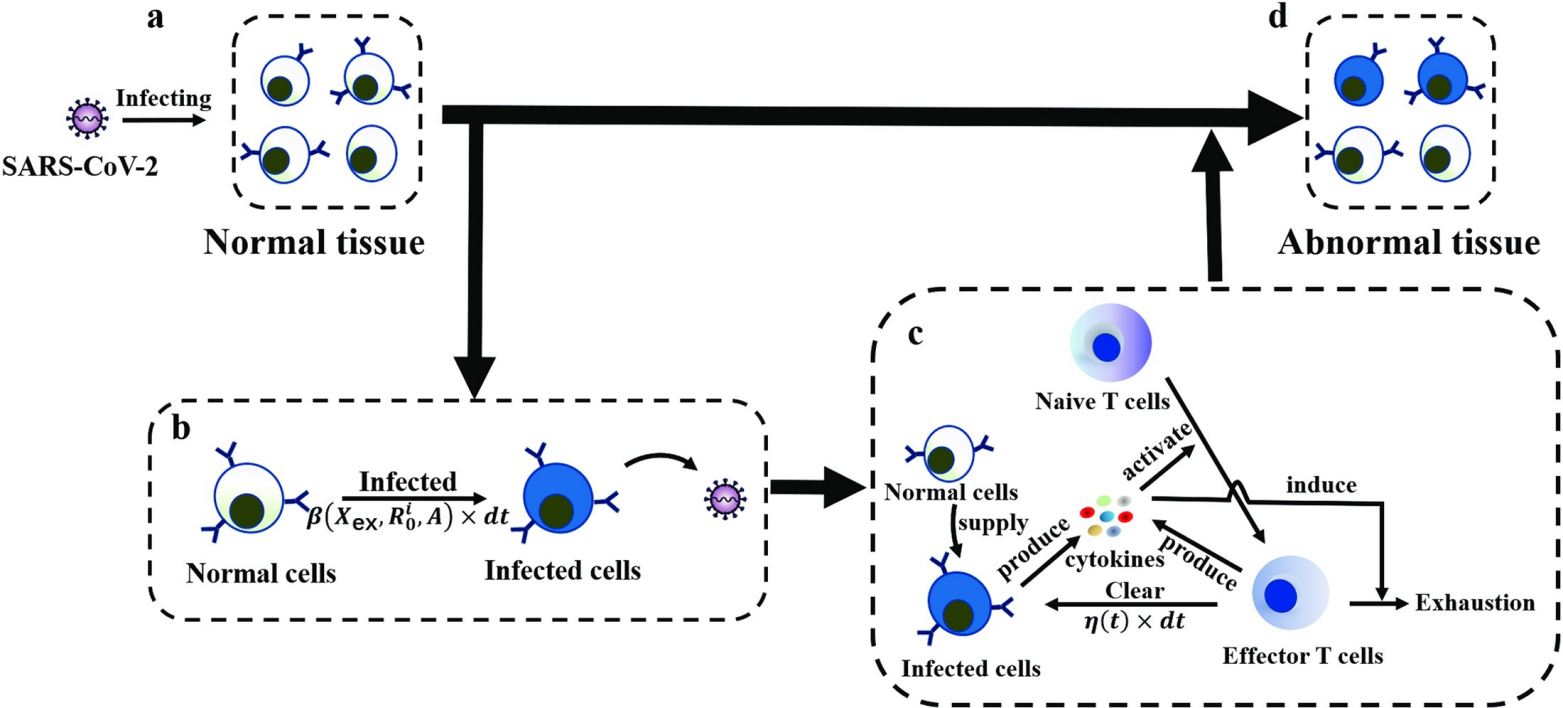
Schematic diagram for the multi-scale modeling of SARS-CoV-2 infection. a. Susceptible cells in normal tissue are infected by SARS-CoV-2. b. Cell states vary from normal to infected. Each normal cell becomes an infected cell with a probability of $\beta (X_{\text{ex}},R_{0}^{i},A)\times dt$, and the infected cell spreads virus to the microenvironment and further infect other susceptible cells. c. The T cell response is triggered by infected cells that secrete cytokines (such as ILs, TNFs, IFNs, etc.). Naïve T cells are activated by cytokines and produce effector T cells to clear infected cells. Meanwhile, the above Eqs (1)–(17) represent the multi-scale model in the current study. This model includes viral dynamics, IFN response, and T cell response after SARS-CoV-2 infection. The viral dynamics and IFN response are coupled through the indexes of infected cells, and the T cell response and cytokines are connected by the number of infected cells. Cytokines are produced by both infected cells and effector T cells, promoting T cell exhaustion. Infected cells are removed with a probability of *η*(*t*) × *dt*. If an infected cell is cleared, a normal cell is generated to keep the total number of target cells unchanged. d. Normal tissue develops into abnormal tissue, and the severity is measured by the ratio of infected cells in the tissue.

The proposed multi-scale model was established using a combination of deterministic and random simulations. The schematic framework is summarized in (Fig 10).

The scheme starts with a tissue system of N normal cells and an initial extracellular SARS-CoV-2 concentration *X*_ex_(0) = *X*_ex,0_. After viral infection, each cell undergoes state change from normal to infected, and is cleaned by effector T cells. When an infected cell is cleaned, a new normal cell is generated to replace the lost cell so that the total cell number *N* remains unchanged. All new normal cells exhibit heterogeneous cell surface receptor protein numbers that obey a gamma distribution $R_{0}^{i}∼\Gamma (\alpha_{1},\alpha_{2})$.

In numerical simulations, we started from t = 0 and simulated the infection progression using a time step dt = 0.01h. At each time interval [*t*, *t* + *dt*], the extracellular virus binds to the receptor proteins on target cells, releases the mRNA into the target cells, replicates the mRNAs and synthesizes new virus, and the intracellular viruses are released into the extracellular environment. Meanwhile, interferons (IFNs) and antiviral proteins (AVPs) are produced and inhibit viral replication in the host cell. These processes are simulated following Eqs (8)–(12). Each cell dynamically changes from normal to infected states following the infections process (Fig 10). At each time step, a normal cell has a probability $\beta (X_{\text{ex}},R_{0}^{i},A)\times dt$ of switching to an infected state and the index set *I*(*t*) of infected cells changes over time. The T cell response is triggered by the infected cells (Fig 10). Infected cells secrete cytokines, such as IL-2, IL-6, TNF-*α*, etc., to induce the activation of naïve T cells. Effector T cells promote the cleaning of infected cells, and secrete cytokines, including IL-2, IL-6, TNF-*α*, etc. Eqs (13) and (14). The cytokines lead to the exhaustion of effector T cells following the exhaustion rate defined by Eq (15). The infected cells are cleaned by effector T cells so that each infected cell is removed with a probability *η*(*t*) × *dt* during an interval [*t*, *t* + *dt*]. When an infected cell is cleaned, a new normal cell is generated to replace the lost cell.

The above processes suggest a numerical scheme shown below (Fig J in S1 Text):

1. **System initialization**: Set the time t = 0 and the step size (dt = 0.01h). Initialize the system states, including the total cell number (*N* = 5000), cell surface receptor protein numbers $R_{0}^{i}$ (following the gamma distribution Γ(*α*_1_, *α*_2_)) in each cell, initial concentration of extracellular SARS-CoV-2 (*X*_ex,0_), naïve T cell number ([*T*_0_]), and the initial conditions $[X_{\text{ex}}](0)=X_{\text{ex},0},R^{i}(0)=0,\;{\;X}_{\text{in}}^{i}(0)=0,{\left[\text{IFNs}\right]}^{i}(0)=0,{\left[\text{AVPs}\right]}^{i}(0)=0,\;[\text{Cytokines}](0)=0,\;[T_{\text{effector}}](0)=0$. Set the states of all cells to be normal (*S*_*i*_ = 0), *N*_infected_ = 0 and the infected cells index *I* to be an empty set.
2. **Update cell states**: For i from 1 to N:
  a) If *S*_*i*_ = 0, changes the cell state to *S*_*i*_ = 1 with a probability $p_{1}=\beta (X_{\text{ex}},R_{0}^{i},A)\times dt$. If the cell state is changed, the index i is added to the index set *I*.
  b) If *S*_*i*_ = 1, cleans the cell with a probability *p*_2_ = *η*(*t*) × *dt*. If the cell is cleaned, the cell state is reset with a newly generated receptor number $R_{0}^{i}∼\Gamma (\alpha_{1},\alpha_{2})$ and the initial conditions *S*_*i*_ = 0, $R^{i}=0,X_{\text{in}}^{i}=0$, [IFNs]^*i*^ = 0, [AVPs]^*i*^ = 0.
  c) If *S*_*i*_ = 1 and is not cleaned, solve the differential equation for *R*^*i*^, [IFNs]^*i*^, [AVPs]^*i*^ and $X_{\text{in}}^{i}$ Eqs (9)–(12) for one step (*t* → *t* + *dt*) using a difference method (e.g., DDE23 in MATLAB), and update the intracellular SARS-CoV-2 concentration $X_{\text{in}}^{i}$, IFNs concentration [IFNs]^*i*^, and AVPs concentration [AVPs]^*i*^.
3. **Update extracellular environment and the host immune response**: Update the infected cells number *N*_infected_, and solve the Eq (8) and Eqs (13)–(15) for one step (*t* → *t* + *dt*) with updated index I and the number *N*_infected_, and update the variables *X*_ex_,[Cytokines], [*T*_effector_].
4. **Update the time**: Let t = t+dt, and either go to step 2 or terminate the simulation process.

### Parameter estimations and sensitivity analysis

Some of the model parameters were obtained directly from published literature, i.e., the association (*k*_on_) and dissociation (*k*_off_) between S protein of SARS-CoV-2, receptor protein were taken as *k*_on_ = 0.6759nM ⋅ h^−1^ and *k*_off_ = 9.9365h^−1^ [67].

Other parameters were estimated by related studies. The mammalian cell volume (*v*_cell_) is 100 ∼ 10000*μ*m^3^ [68] and the density of naïve T cell is approximately 4 × 10^−3^ *g*/*c*m^3^ [69] so that the counts of naïve T cell ([*T*_0_]) are about 0.4 ∼ 4.0 × 10^5^cells/ml. The half-life of IFNs, AVPs and SARS-CoV-2 were determined from published studies; the half-life of IFNs ranged from 1.3 to 4.7 hours [70], AVPs is 2∼24 hours [29], and the half-life of SARS-CoV-2 is about 6.8 hours [71]. By the natural depletion rate *δ* = ln2/*t*_1/2_ (*t*_1/2_ is the half-life), the degradation of IFNs, AVPs and SARS-CoV-2 were estimated as *δ*_1_ = 0.1h^−1^, *δ*_2_ = 0.4h^−1^, *δ*_3_ = 0.12h^−1^, respectively. Extracellular viruses are easier to be clean by the humoral and cell-mediated immune response so that we set *δ*_4_ = 2.5*δ*_1_ = 0.25h^−1^.

In Eq (7), parameters were set to *m*_0_ = 5, *K*_0_ = 48 × 10^−11^, *β*_0_ = 0.15 to satisfy the effect of threshold between the virus and receptor protein (Fig Ka in S1 Text), *R*_0_ in the Eq (1) was assumed to obey a gamma distribution Γ(*α*_1_, *α*_2_) and the parameters of *α*_1_, *α*_2_ were estimated using the max likelihood estimation (MLE) (*α*_1_ = 12.11, *α*_2_ = 9.50) (Fig Kb in S1 Text).

We estimated other parameters by fitting simulation results with clinical data. We fitted clinical data of T cell dynamics from dataset 7 to estimate the remaining parameters and patients with different symptom presentation by varying the exhaustion rate (*ρ*) (Moderate: *ρ* = 0.0005; Severe: *ρ* = 0.0025; Critical: *ρ* = 0.005) (Fig L in S1 Text). Before fitting the data, we assumed that the initial time of patients in the hospital (*t* = *T*_IP_ + *t*_0_) and *t*_0_ represents a time interval from showing symptom to being hospitalized. The parameter *t*_0_ was taken as 1 day based on the information of dataset 7. The parameters were estimated within relevant biological ranges such that the number of effector T cells was well fitted to patients with different symptoms. A detailed description and default values of the model parameters are shown in Table A in S1 Text. In addition, we examined our model using two indexes: Q and L (the definitions of these indexes is in Section 3 in S1 Text). Details of model validation are provided in the Section 9 in S1 Text. The results displayed in Table C and Figs L and M in S1 Text suggest that the accumulation effect (15) is appropriate for true dynamics of effector T cells in COVID-19 patients.

We applied the method of partial rank correlation coefficient (PRCC) [72] to perform sensitivity analysis for estimated parameters related to viral dynamics. Sensitivity analysis was performed using 200 sample runs and a perturbation magnitude of 0.1. The sensitivities of input parameters to the ratio of infected cells (*R*_IC_) on day 30 after infection were calculated (Fig D in S1 Text). The most sensitive parameters λ_1_ and *b*_1_ correspond to the rate of viral RNA replication and are attributed to the characteristics of the virus itself. The parameters λ_2_ and *K*_1_ are also significant, which correspond to IFN response and the coefficient of inhibiting viral replication, respectively. We also note the two parameters *v*_ex_ and *δ*_4_ that were associated with the process of susceptible cells infected by SARS-CoV-2.

### Treatment model

The strategies of treatment for COVID-19 are primarily classified as antiviral treatment and immune modulation. Antiviral treatment methods include type I interferons and Arbidol [44], and immune modulations include immunoglobulins and hormone treatment [73]. Potential immune therapy, such as blocking the inhibitory immune checkpoint molecules, has been applied in severe cases [40]. To model the therapeutic effects, we considered four treatment strategies, including acceleration of IFN response, restriction of viral replication, promotion of extracellular virus clearance, and inhibition of T cell exhaustion. We introduced a quadruple (*ε*_1_, *ε*_2_, *ε*_3_, *ε*_4_) to represent the effects of the above four strategies. Thus, the equations of the preceding model were modified as follows:

$$\begin{array}{c} \frac{d{\left[\text{IFNs}\right]}^{i}}{dt}=(1+\varepsilon_{1})\lambda_{2}X_{\text{in}}^{i}+b_{2}\frac{{\left({\left[\text{IFNs}\right]}^{i}\right)}^{m_{2}}}{{\left({\left[\text{IFNs}\right]}^{i}\right)}^{m_{2}}+K_{2}^{m_{2}}}-\delta_{2}{\left[\text{IFNs}\right]}^{i} \end{array}$$ (18)

$$\begin{array}{ccc} \frac{dX_{\text{in}}^{i}}{dt} & = & \frac{k_{\text{in}}R^{i}}{v_{\text{cell}}}+\lambda_{1}X_{\text{in}}^{i}(t-\tau_{1})\frac{b_{1}{\left((1-\varepsilon_{2})K_{1}\right)}^{m_{1}}}{{\left((1-\varepsilon_{2})K_{1}\right)}^{m_{1}}+{\left({\left[\text{AVPs}\right]}^{i}\right)}^{m_{1}}}\\ & & -\delta_{1}X_{\text{in}}^{i}-q_{0}X_{\text{in}}^{i}(t-\tau_{2}) \end{array}$$ (19)

$$\begin{array}{ccc} \frac{dX_{\text{ex}}}{dt} & = & \frac{v_{\text{cell}}}{V_{\text{ex}}}\sum_{i\in I(t)}q_{0}X_{\text{in}}^{i}(t-\tau_{2})+\frac{1}{V_{\text{ex}}}\sum_{i\in I(t)}\left[k_{\text{off}}R^{i}-k_{\text{on}}\left(R_{0}^{i}-R^{i}\right)X_{\text{ex}}\right]\\ & & -\delta_{4}\left(1+\varepsilon_{3}\right)X_{\text{ex}} \end{array}$$ (20)

$$\begin{array}{c} \delta_{\text{effector}}([\text{Cytokines}])=\delta_{5}+\rho H(t-t_{2})\int_{t-\tau_{3}}^{t}\frac{{\left[\text{Cytokines}](s\right)}^{m_{4}}}{{\left[\text{Cytokines}](s\right)}^{m_{4}}+{\left(\left(1+\varepsilon_{4}\right)K_{4}\right)}^{m_{4}}}ds \end{array}$$ (21)

Here, *ε*_1_(*ε*_1_ > 0) represents acceleration of the IFN response, *ε*_2_(0 < *ε*_2_ ≤ 1) represents the restriction of viral replication, *ε*_3_ (*ε*_3_ > 0) represents the promotion of extracellular virus clearance, and *ε*_4_ (*ε*_4_ > 0) represents inhibition of T cell exhaustion. Moreover, we assumed that a patient starts the treatment on day *t*_3_ = 15 after infection, so that the above equations were applied when *t* ≥ *t*_3_. The Eqs (18)–(21) were used to explore the treatment dynamics for COVID-19 patients.

## Supporting information

S1 Text Supplementary materials. (PDF)

S2 Text Source codes of the numerical scheme. (PDF)
