# Supplementary material for: Data-driven multi-scale mathematical modeling of SARS-CoV-2 infection reveals heterogeneity among COVID-19 patients
Source: PLoS Comput Biol. 2021 Nov 24;17(11):e1009587. doi: 10.1371/journal.pcbi.1009587 (PMC8654229; doi:10.1371/journal.pcbi.1009587)
Supplement: S1 Text — (PDF) [file pcbi.1009587.s001.pdf]

Supplementary Materials

Data-driven multi-scale mathematical modeling of SARS-CoV-2 infection reveals heterogeneity of COVID-19 patients

Shun Wang[1, 2], Mengqian Hao[1, 2], Zishu Pan[3], Jinzhi Lei[4*] & Xiufen Zou[1, 2*]

Content

Section 1 Integration analysis of experimental and clinical data 1
Section 2 Model 2
Section 2.1 Model description 3
Section 2.2 Deviation of infected rate 5
Section 2.3 Dynamics of recovery 6
Section 3 Parameter estimation 6
Section 4 Source codes 8
Section 5 Cell heterogeneity 8
Section 6 Quantitative T cell exhaustion 9
Section 7 Treatment for COVID-19 10
Section 7.1 Metric for treatment efficacy 10
Section 7.2 Quantitative treatments 10
Section 8 Other factors' influence on asymptomatic and Incubation period 11
Section 9 Model Validation 12
Section 10 Supplementary Tables 13
Section 11 Supporting Figures 16

Section 1 Integration analysis of experimental and clinical data

In this study, multi-source experimental and clinical datasets of patients with COVID-19 were available from published studies (Details in Method). Some datasets were used to develop the multiscale model of SARS-CoV-2 infection, and other datasets were used to validate the model. Here, we introduced how these datasets are linked with our model.

We obtained epidemiological information when patients were exposure and presented symptom from dataset 1. For simplicity, incubation period is approximately considered as the time period from starting exposure to showing symptom. According to the data of incubation period, we validated that the threshold of asymptomatic and symptomatic state is well defined in our model.

As for datasets 2, 3, 4 and 5, they mainly include clinical information of immune microenvironment from different types of patients. According to the analysis of these datasets, we proposed three assumptions to establish our model.

- **The depletion of T cell counts is associated with T cell exhaustion**. Based on the analysis of dataset 4, effector T cells including $CD4^{+}$ and $CD8^{+}$ T cells in severe cases are significantly lower than those in moderate cases. Previous studies have showed that exhaustion of T cells is linked with the severity of COVID-19 [1,2], we gained directly the information of biomarkers or T cell exhaustion, PD-1 and Tim-3, from datasets 2 and 5. The expression of Tim-3 in severe or critical patients shows obviously higher level than that in mild or moderate patients (**Figs 1g-h** in main text). We noted that the expression level of PD-1 in severe or critical patients is insignificantly difference from dataset 5 compared with PD-1 expression from dataset 2 (**Fig B**). Despite of the differences, our assumption is still acceptable.
- **T cell exhaustion is dependent on the density of cytokines.** Before the analysis of datasets 3 and 4, we picked cytokines included in both datasets 3 and 4. Notably, cytokines measured in dataset 3 are showed in **Fig A**. Based on significant examination of these cytokines, IL-6, IL-10, and IFN-γ presented obvious higher level in death or severe patients than in survival or moderate patients (**Fig A**). We also noted that IL-2, IL-4 and TNF-α are uncertainty for the development of COVID-19 (**Fig C**). These results demonstrate that high level of cytokines IL-6, IL-10, and IFN-γ may lead to the malignant development of COVID-19. Persistent high level cytokines is known to induce the exhaustion of T cells [3].
- **The comprehensive effect of IL-6, IL-10, and IFN-γ is represented by a variable of cytokines.** Because the relationship between IL-6, IL-10, and IFN-γ is unidentified by current datasets, we assumed a variable to replace the overall effect of IL-6, IL-10, and IFN-γ for simplicity.

Datasets 6 and 7 are used to estimate some parameters in our model. In our model, we assumed that cell heterogeneity is represented by various receptor proteins on the surface of cells. More information of receptor proteins is required to be determined. ACE2 and NRP1 are receptors for S protein of SARS-CoV-2 [4,5]. Dataset 6 includes proteomic information from patients with COVID-19. We assumed that the amount of receptor protein is approximately equivalent to the abundant of NRP1 in our model. Dataset 7 obtained by software GetData (http://www.getdata-graph-digitizer.com/) includes dynamic of T cells in patients with moderate, severe and critical, and were applied to determine other parameters in our model. Details of parameter estimation are in Section 3.

Section 2 Model

We combined the major processes with a model of host immune dynamics to develop a hybrid multi-scale model of SARS-CoV-2 infection. The model describes the immune

dynamics through two parts: T cell immunity and type I interferon innate immunity, which is based on virus-triggered immune dynamics [6,7]. We integrate these dynamics with individual heterogeneity due to IFN-I response [8] and T cell response [2]. Besides, we considered cell heterogeneity due to receptor expression on the surface of target cells. With time course after infection, the population size of infected cells changes according to viral infection and cell removal (due to T cell immunity) events. In addition, we performed stochastic modeling for the effects of viral infection and cell clearance. The model includes three components: type I interferon regulatory network, T cell response, and cell status transition. Main process of the three components are listed below:

Cell status transition

We divided all cells into normal and infected cells in the model. Viral infection and cell clearance are assumed to be stochastic [9]. Whether a cell is infected by virus is dependent on the viral density in the extracellular environment and the receptor expression level on the cell surface. The clearance of infected cells is dominated by the concentration of effector T cells.

Type I interferon regulatory network

When viral RNA is released into the cytoplasm, type I interferon is activated and stimulates downstream signaling pathway to produce antiviral protein. Anti-viral protein inhibits viral replication to protect organism.

T cell response

The infected cells secrete cytokines and activate the naïve T cells to produce effector T cells. The effector T cells continuously clear the infected cells and secrete cytokines, which may lead to chronic inflammation and further induce the exhaustion of effector cells [10].

Section 2.1 Model description

The formulations of partial biological processes in our model are described as integrodifferential equations as follows:

$$\frac{dR^i}{dt} = \underbrace{k_{\text{on}}\left(R_0^i - R^i\right)X_{\text{ex}}}_{\text{associated with free receptor}} - \underbrace{k_{\text{off}}R^i}_{\text{dissociated with receptor}} - \underbrace{k_{\text{in}}R^i}_{\text{RNA release}} \quad (1)$$

$$\frac{dX_{\text{in}}^i}{dt} = \underbrace{\frac{k_{\text{in}}R^i}{v_{\text{cell}}}}_{\text{RNA released into cell}} + \underbrace{\lambda_1 X_{\text{in}}^i(t-\tau_1)\frac{b_1 K_1^{m_1}}{K_1^{m_1} + ([\text{AVPs}]^i)^{m_1}}}_{\text{antiviral protein inhibition to viral replication}} - \underbrace{\delta_1 X_{\text{in}}^i}_{\text{RNA degradation}} - \underbrace{q_0 X_{\text{in}}^i(t-\tau_2)}_{\text{viral budding}} \quad (2)$$

$$\frac{d[\text{IFNs}]^i}{dt} = \underbrace{\lambda_2 X_{\text{in}}^i}_{\text{activated by viral RNA}} + \underbrace{\frac{b_2\left([\text{IFNs}]^i\right)^{m_2}}{K_2^{m_2} + ([\text{IFNs}]^i)^{m_2}}}_{\text{positive autoregulation of IFN}} - \underbrace{\delta_2 [\text{IFNs}]^i}_{\text{IFN degradation}} \quad (3)$$

$$\frac{d[\text{AVPs}]^i}{dt} = \underbrace{\lambda_3 [\text{IFNs}]^i}_{\text{synthesis by IFN}} - \underbrace{\delta_3 [\text{AVPs}]^i}_{\text{degradation of AVP}} \qquad \left(i \in I(t)\right) \quad (4)$$

$$\frac{dX_{\text{ex}}}{dt} = \underbrace{\frac{v_{\text{cell}}}{V_{\text{ex}}}\sum_{i\in I(t)} q_0 X_{\text{in}}^i(t-\tau_2)}_{\text{progeny virus budding to extracell}} + \underbrace{\frac{1}{V_{\text{ex}}}\sum_{i\in I(t)}\left[k_{\text{off}}R^i - k_{\text{on}}\left(R_0^i - R^i\right)X_{\text{ex}}\right]}_{\text{association and dissociation of virus and receptors}} - \underbrace{\delta_4 X_{\text{ex}}}_{\text{virus cleaning}} \tag{5}$$

$$\frac{\mathrm{d}[T_{\text{effector}}]}{\mathrm{d}t} = \underbrace{\mu_1\frac{[\text{Cytokines}]^{m_3}}{[\text{Cytokines}]^{m_3}+K_3^{m_3}}[T_0]}_{\text{the differential of naive T cells acitved by cytokines}} - \underbrace{\delta_{\text{effector}}([\text{Cytokines}])[T_{\text{effector}}]}_{\text{T cell death and exhaustion}} \tag{6}$$

$$\frac{\mathrm{d}[\text{Cytokines}]}{\mathrm{dt}} = \underbrace{\mu_2 R_{\text{IC}}}_{\text{produced by infected cells}} + \underbrace{\mu_3[T_{\text{effector}}]}_{\text{produced by effector T cells}} - \underbrace{\delta_6[\text{Cytokines}]}_{\text{degradation of cytokines}} \tag{7}$$

$$\delta_{\text{effector}}([\text{Cytokines}]) = \underbrace{\delta_5}_{\text{basic death rate}} + \underbrace{\rho H(t-t_2)\int_{t-\tau_3}^{t}\frac{[\text{Cytokines}](s)^{m_4}}{[\text{Cytokines}](s)^{m_4}+K_4^{m_4}}\mathrm{d}s}_{\text{T cell exhaustion rate}} \tag{8}$$

$$H(t-t_2) = \begin{cases}1 & t > t_2\\ 0 & t \le t_2\end{cases} \tag{9}$$

The equations **(1)-(4)** describes intracellular virus-trigged type I interferon pathways and we denoted $I(t) \subseteq \{1,2,...,N\}$ as the set of indexes of infected cells at time *t*. With respect to the *i*th infected target cell $\left(i \in I(t)\right)$, we denoted intracellular virus RNA concentration X_{in}^i, interferons concentration $[\text{IFNs}]^i$, antiviral protein concentration $[\text{AVPs}]^i$, and cell surface free receptor protein number R^i. For simplicity, we assumed that each receptor protein on target cell surface can only bind to one spike protein of SARS-CoV-2, and the receptor protein is freed when SARS-CoV-2 releases its RNA into the host cell and the spike protein dissociates from the receptor protein (**Eq. (1)**). SARS-CoV-2 releases its RNA to the host cell after the spike protein is bound to receptor of the target cell, then the RNA of SARS-CoV-2 directs RNA replication and virus assembly using organelles and synthase from the host cell. We assumed that there is a time delay (τ_1) in the processes of virus replication because of involved multi-step reactions. The inhibition of viral replication is described by a Hill type function. Progeny virus is assembled by organelles, and synthase from the host cell after viral replication. Since the process involves multistep reactions, we assumed a lag time (τ_2) for the process of virus budding. These processes of viral RNA inside a host cell are described by **Eq. (2)**. Within the process of virus replication, interferon signaling pathway is activated to produce interferons (IFNs) and antiviral proteins (AVPs), resulting in limited virus replication. The interferons (IFNs) are activated by viral nuclear and has positive autoregulation (**Eq. (3)**), and the antiviral proteins (AVPs) are synthesized in the IFNs downstream signaling pathways and are degraded naturally in the host cell (**Eq. (4)**).

The equations **(5)-(9)** describe intracellular biological processes, including mainly the extracellular viral concentration and T cell response. We denoted the extracellular virus RNA concentration X_{ex}, cytokines concentration [Cytokines] and density of effector T cells $[T_{\text{effector}}]$. The extracellular virus involves the processes of association and dissociation with receptor proteins on the infected cells, and progeny virus produced by infected cells (**Eq. (5)**). The infected cells secrete cytokines and activate

the naïve T cells to produce effector T cells. The effector T cells continuously clear the infected cells and secrete cytokines, which may lead to chronic inflammation and further induce the exhaustion of effector T cells [2]. We assumed a constant naïve T cells number $[T_0]$ over time for simplicity[11]. The biological processes are described by **Eqs. (6)-(7).** In the equations, R_{IC} denotes the ratio of infected cells over total cells, $\delta_{\mathrm{effector}}([\mathrm{Cytokines}])$ represents the rate of effector T cell exhaustion/cleaning (**Eq. (8)**). The Heaviside function $H(t-t_2)$ (**Eq. (9))** is introduced to represent the starting of T cell exhaustion, where $t_2 = T_{IP}$ indicates the starting time of T cell exhaustion from a symptomatic status. Clinically, T cell exhaustion is associated with the expression of specific immune-inhibitory factors, including PD-1 and Tim-3 on cell surface [1,12]. The origin of T cell exhaustion remains unclear and hence details are omitted here [13,14].

The intracellular and intercellular models are coupled by the extracellular virus (X_{ex}) and the ratio of infected cell (R_{IC}). The concentration of extracellular virus (X_{ex}) is described by Eq. **(5)**, while the biological processes of infected cells are stochastic events include cell infection in a rate $\beta(X_{\mathrm{ex}}, R_0^i, A)$ and the clearance of infected cells in a rate $\eta([T_{\mathrm{effector}}])$ in the intercellular model.

Section 2.2 Deviation of the infected rate

First, we assumed that $R^i, X_{\mathrm{ex}}, R_0^i$ and $A = k_{\mathrm{off}}/k_{\mathrm{on}}$ respectively represent the receptor proteins binding to SARS-CoV-2 for the ith target cell, viral density in the extracellular environment, total number of receptors on the surface of the ith target cell and the binding affinity. The relationship among them is simplified as

$$X_{\mathrm{ex}} + R_0^i \underset{k_{\mathrm{on}}}{\overset{k_{\mathrm{off}}}{\rightleftarrows}} R^i$$

At the equilibrium, we have

$$R^i = \frac{k_{\mathrm{on}} X_{\mathrm{ex}} R_0^i}{k_{\mathrm{off}} + k_{\mathrm{on}} X_{\mathrm{ex}}} \tag{10}$$

Further, we considered a threshold effect between receptor protein and virus, and proposed a Hill type function for the infected rate of the ith target cell (β^i) as follows:

$$\beta^i = \beta_0 \frac{(R^i)^{m_0}}{(R^i)^{m_0} + K_0^{m_0}} \tag{11}$$

Substituting (10) into (11), we write

$$\beta(X_{\mathrm{ex}}, R_0^i, A) = \beta_0 \frac{(X_{\mathrm{ex}} R_0^i)^{m_0}}{(X_{\mathrm{ex}} R_0^i)^{m_0} + \left(K_0 (X_{\mathrm{ex}} + A)\right)^{m_0}} \tag{12}$$

here, K_0, m_0 are constants. The binding affinity measures the equilibrium dissociation constant between S protein on SARS-CoV-2 and the receptor proteins on target cells.

In our model, all cells are initially normal and un-infected, and once a cell is recognized by SARS-CoV-2 and the infection is initiated, the status of the cell becomes infected.

Section 2.3 Dynamics of recovery

In the current medication for treatment of COVID-19, there is an antiviral medication that stimulates the phagocytic function of macrophages to clear infected cells, such as Umifenovir [15]. In our model, we set the parameter ε_5 in the cleared rate of T cells $\eta([T_{\text{effector}}])$ to quantify this antiviral treatment. The formulation was written as

$$\eta([T_{\text{effector}}]) = \eta_0[T_{\text{effector}}] + \varepsilon_5$$

We simulated the dynamics of the antiviral treatment for moderate patient $(\rho = 0.0005)$ under the deterministic value $(\varepsilon_5 = 0.025)$, and this result exhibited that both $\mathrm{X_{ex}}$ and $\mathrm{R_{IC}}$ decreased to zero on day 15 after treatment (**Fig P**).

Section 3 Parameter estimation

Some parameters were estimated by published relevant studies, which are listed the source in Table A. The association (k_{on}) and dissociation (k_{off}) between S protein of SARS-CoV-2, receptor protein were taken as $k_{\text{on}} = 0.6759\ \text{nM} \cdot h^{-1}$ and $k_{\text{off}} = 9.9365\ h^{-1}$ [5]. The mammalian cell volume (v_{cell}) is 100~10000 μm^3 [16] and the density of naïve T cell is about $4 \times 10^{-3}\ \text{g/cm}^3$ [11], the counts of naïve T cell $([T_0])$ is about $0.4{\sim}4.0 \times 10^5$ cells/ml. The half-life of IFNs, AVPs and SARS-CoV-2 were determined from published studies; the half-life of IFNs is ranged from 1.3 to 4.7 hour [17], that of AVPs is about 2~24 hour [7], and the half-life of SARS-CoV-2 is about 6.8 hour [18]. From the natural depletion rate $\delta = \ln 2/t_{1/2}$ ($t_{1/2}$ is the half-life), the degradation rates of IFNs, AVPs and SARS-CoV-2 were estimated as $\delta_1 = 0.1\ h^{-1}, \delta_2 = 0.4\ h^{-1}, \delta_3 = 0.12\ h^{-1}$, respectively. Extracellular viruses are easier to be cleaned by humoral and cell-mediated immune response, and hence we set $\delta_4 = 2.5\delta_1$. In Eq.(1), parameters were set to $m_0 = 5, K_0 = 48 \times 10^{-11}, \beta_0 = 0.15$ to satisfy the effect of threshold between virus and receptor protein **(Fig Ka)**, R_0 in the Eq.(1) was assumed to obey a gamma distribution $\Gamma(\alpha_1, \alpha_2)$, the parameters of α_1, α_2 were estimated by the means of max likelihood estimation (MLE) $(\alpha_1 = 12.11, \alpha_2 = 9.50)$ **(Fig Kb)**.

The remaining parameters were regulated to fit the dataset 7, which includes kinetic data of effector T cells from moderate (410 cases), severe (206 cases) and critical (91 cases) during patients in the hospital. We assumed that the initial time of patients in the hospital $(T_{IP} + t_0)$, and t_0 represents a time interval from showing symptom to being hospitalized. The parameter t_0 was taken as 1 day based on the information of dataset 7. Dataset 7 gives median and interquartile range of counts of effector T cells at five

different time after illness onset. We proposed two indexes $L = \sum_{i=1}^{n} \chi(T_{\text{sim}}(t_i))_{(T_{1/4}(t_i),T_{3/4}(t_i))}$ and $Q = \sqrt{\sum_{i=1}^{n} \left(\frac{T_{\text{sim}}(t_i)-T_{1/2}(t_i)}{T_{1/2}(t_i)}\right)^2}$ where $T_{\text{sim}}(t_i)$ is the density of effector T cells from our model, $T_{1/4}(t_i)$, $T_{1/2}(t_i)$ and $T_{3/4}(t_i)$ are the first, second and third quartile density of effector T cells at time t_i from real data, and *n* is the total number of real data. The value of $\chi(T_{\text{sim}}(t_i))$ is one if $T_{\text{sim}}(t_i)$ is in the interval $(T_{1/4}(t_i), T_{3/4}(t_i))$, otherwise the value of $\chi(T_{\text{sim}}(t_i))$ is zero. The large value of L represents that numerical simulation is much possible in the range of real data. The small value of Q represents that numerical simulation approaches the median level of real data. We selected the greedy algorithm at the appropriate parameter space to search remaining parameters. Numerical schemes for parameter estimation are listed below:

1. Estimating the remaining parameters by maximizing the index L and minimizing the index Q with data of effector T cells in moderate patients from dataset 7.
2. Adjusting the exhaustion rate ρ alone by maximizing the index L and minimizing the index Q with the data of effector T cells in severe patients from dataset 7.
3. Similar process as step 2 for critical patients from dataset 7.

Using the above method, we searched the exhaustion rate corresponding to moderate, severe and critical (Moderate: $\rho = 0.0005$; Severe: $\rho = 0.0025$; Critical: $\rho = 0.005$), and the remaining parameters in Table A. The numerical results show that the dynamics of effector T cells are in the range of data from severe and critical patients when model is simulated by the estimated parameters **(Fig L)**.

Considering the different scales between dataset 7 and estimated parameters of viral replication, we applied the method of partial rank correlation coefficient (PRCC) [19] to perform sensitive analysis for estimated parameters related with viral dynamics. Sensitivity analysis was performed with 200 sample runs and a perturbation magnitude of 0.1. The sensitivity of input parameters to the ratio of infected cell (R_{IC}) at day 30 after infection were calculated **(Fig D)**. The most sensitive parameters λ_1 and b_1 correspond to the rate of virus RNA replication and is attributed to the characteristics of the virus itself. The parameters λ_2 and K_1 are significant as well, which correspond to IFN response and coefficient of inhibiting virus replication, respectively. We also note the two parameters v_{ex} and δ_4 that associated with the process of susceptible cells infected by SARS-CoV-2.

Further, we perform a perturbation to above four parameters on the default value and use these indexes, including R_{IC}, [Cytokine] and $[T_{\text{effector}}]$ on day 30, to study how these parameters affect the progression of SARS-CoV-2 infection. As exhibited in **Figs Ra-d**, most of patients shows symptomatic manifestation and a few are symptomatic among these parameters. However, λ_1 and b_1 that are linked with the virus itself result in more asymptomatic individuals (**Figs Ra-b**). Interestingly, we noted that both symptomatic and asymptomatic manifestation are present when the value of λ_1 is assigned around 0.145 (**Fig Rb**). At the view of immunological

microenvironment, these results of both cytokines and effector T cells are in agreement with R_{IC}, yet we noted that a few symptomatic patients show low level of cytokine and effector T cells (**Figs Re-l**).

Besides, we examine whether the total number of targeted cells (N) have influence on numerical results. We perform a simulation of 200 individuals with N increased by 50%, then compare with the result of N assigned with default value using the indexes including the incubation period (T_{IP}) and the distribution of R_{IC} on day 30. Using the Kolmogorov-Smirnov test, and the result exhibits that the change of N is insignificant difference on the both incubation period (T_{IP}) and the distribution of R_{IC} on day 30 (**Figs Sa-b**).

Section 4 Source codes

The code of our model is composed of four parts that are main function ("BCTool.cpp"), single cell function ("CCell.cpp"), system function ("System.cpp") and random number ("Random.cpp"), which are programed by C++ programming. All parameters are saved in two files: "par.dat" and "md.in". The running command is controlled by the shell script "run.sh". The numerical results are integrated and plotted by MATLAB. The more details of code have been deposited at the GitHub repository (https://github.com/WilliamMoriaty/Model-COVID-19).

Section 5 Cell heterogeneity

In the model, we supposed that cell heterogeneity is characterized by different receptor expressions on the surface of each target cell. According to proteomic data from dataset 6, the distribution of receptor expression is approximately gamma distribution.

To examine the assumption of cell heterogeneity, we set three groups of low homogeneity, high homogeneity and heterogeneity, and simulate 200 individuals for each group, each individual is infected by SARS-CoV-2 with a time course of 30 days. Homogeneity refers to the same receptor expression on the surface of each target cell. Low homogeneity represents the case in which the value of receptor expression is about the average of gamma distribution, while high homogeneity represents the case in which the value of receptor expression is increased by 20% on the basis of the average of gamma distribution.

The numerical results were shown in **Fig N,** all individuals in the group of low homogeneity are in low level of R_{IC} and were concentrated in about 0.01. In contrast, R_{IC} in the group of high homogeneity increased rapidly at the beginning and decreased to about 0.3 with the pressure of T cell immunity. The case of heterogeneity exhibited diverse disease progressions. Based on the definition of symptomatic and asymptomatic status in our study, all cases of low homogeneity exhibited asymptomatic state, while

all cases of high homogeneity exhibited symptomatic state on day 30 after infection. But, the group heterogeneity exhibited that a few individuals exhibited symptomless though most of individuals were symptomatic state on day 30 after infection, which is in agreement with the fact that there are a few patients with long incubation period [20].

Section 6 Quantitative T cell exhaustion

To further explore the effect of T cell exhaustion on COVID-19 progression, we introduced a concept of transition time that refers to the first passage time from onset to severe cases. In the model, we defined severe symptom as the case with the value of R_{IC} in the range from 0.4 to 0.8, and the onset of symptom refer to situation when the value of R_{IC} is larger than 0.05. Thus, the transition time is defined as $(t_2 - t_1)$, where t_2 is the minimum time when R_{IC} is over 0.4, and t_1 is the minimum time when R_{IC} is over 0.05. We varied the parameters in the (ρ, K_4) plane, and searched values of ρ and K_4 that may yield severe individuals. Next, we stochastically simulated 100 individuals for each pair of ρ and K_4, and calculated the mean and standard of the corresponding transition time. In **Fig F**, the result indicated that the transition time decreases with increasing the level of T cell exhaustion, while the transition time lengthens when the level of anti-exhaustion of T cells increases.

On the other hand, we noted that value of R_{IC} increase rapidly with either the increase of ρ or the decrease of K_4. We set 26 individuals with different values of ρ or K_4 and ran with both asymptomatic initial state $(R_{\mathrm{IC}} = 0)$ and severe initial state $(R_{\mathrm{IC}} = 1)$. The results exhibited in **Fig 6** indicated that there is bistable for cytokines and R_{IC} in the range of $85 < K_4 < 98$ when $\rho = 0.0025$ **(Figs 6a-b)**. Similarly, there is a bistable for cytokines and R_{IC} in the range $0.0018 < \rho < 0.0024$ when $K_4 = 84$ **(Figs 6d-e)**. These results imply that there is risk of transition from mild-moderate to severe symptoms. It is interesting that effector T cells number do not show obvious bistability **(Figs 6c and 6f)**

In addition, we examined the quantitative curve of cytokines with the change of T cell exhaustion (ρ) (**Fig Q**). The dose response curve was formulated as

$$[\text{Ctyokines}] = Z_{\max}\frac{\rho^n}{\rho^n + \Lambda^n} + Z_0$$

here, $Z_{\max}$ is the maximum concentration of cytokines at day 30 after infection, Λ is EC50 for ρ, Z_0 is constant and n is constant. We fitted simulation results with the above formula with MATLAB ToolBox, all parameters are listed in **Fig Q**. The result indicated that the threshold of cytokines between mild-moderate and severe case approaches EC50 of the dose curve.

Section 7 Treatment for COVID-19

The treatment strategies for COVID-19 are mainly classified as antiviral treatment and immune modulation. Antiviral treatment methods include type 1 interferons and Arbidole, and immune modulations include immunoglobulins and hormone treatment [21]. Potential immune therapy, such as blocking the inhibitory immune checkpoint molecules, is applied for severe cases [22].

Section 7.1 Metric for treatment efficacy

To evaluate the effect of treatments, we considered changes in the population size of infected cells and immune microenvironment after treatment. In the model, we calculated the concentrations of cytokines and effector T cells at the day 15 after proposed treatments, with comparison to cases without treatment. In **Fig Ga**, the results exhibited that, for moderate patients, both concentration of cytokines and effector T cells number obviously decreased after high efficacy treatments, while increasing the resistance of T cell exhaustion remained high concentration of cytokines and number of effector T cells. However, for severe cases **(Fig Gb)**, both the concentration of cytokines and the number of effector T cells were unchanged after single antiviral treatments, but obviously decreased to low levels after treatments with combined therapy. Moreover, cytokines concentration clearly decreased and the number of effector T cells remained while inhibition of T cell exhaustion was applied. At the view of immune microenvironment, both cytokines and effector T cells obviously improve after high efficacy treatments.

Section 7.2 Quantitative treatments

To further quantify the efficacy of four treatments, we simulated 50 moderate patients and 50 severe patients with various values of four parameters related to treatment, and fit the corresponding dose curves of the four treatments. First, when ρ was taken as 0.0005 and other parameters were default in Table A, individual evolved to moderate cases after infection as in Section 3. For severe case, the parameter ρ was taken as 0.0025 and other parameter were default in Table A.

For single treatment, we randomly selected values of one therapeutic parameter and other therapeutic parameters were taken as zero, and simulated 50 moderate/severe cases for the same value of treatment parameter. We calculated the average efficacy for every treatment and fitted the dose response curve for every single treatment. The formulation of dose response curve is written as

$$\mathrm{E}(\varepsilon_i) = E_i^0 \frac{\varepsilon_i^n}{\varepsilon_i^n + \Lambda_i^n}$$

here, E_i^0 is the maximum efficacy for ε_i, Λ_i is EC50 for ε_i and n is a constant. We fitted the data with MATLAB ToolBox, all parameters are listed in Table B. The results indicated that the maximum efficacy of targeting IFN response (ε_1), viral replication (ε_2), and virus clearance (ε_3) was close to 1 **(Figs Ha-c and He-g)**, , but the maximum efficacy of targeting T cell exhaustion (ε_4) only reached 0.8 **(Fig Hh)** for severe case and was even less at 0.3 in moderate cases **(Fig Hd)** , suggesting that antiviral treatment is more effective than immunological treatment for both moderate and severe cases. We fitted the dose response curves with Hill functions, and compared the Hill coefficient n and EC50 of the functions for moderate and severe cases in response to the same treatment strategy (Table B). The coefficient n in moderate cases was generally smaller than in severe cases, and the value of EC50 in severe cases was generally larger than in moderate cases, implying that moderate cases are more sensitive to treatment than severe cases, and higher doses are required for severe cases. We also noted that efficacy of ε_2 and ε_3 were not suitable for the dose curve we set **(Figs Hf-g),** there may be an alternative formulation for them.

Although single antiviral treatment is effective for both moderate and severe cases, the required dose is large, especially for severe cases. We further considered different combinations of four treatments to reduce the dose and maintain high levels of treatment efficacy. On the one hand, the result of united antiviral treatments **(Figs 9a, 9b, and 9d)** demonstrated that the antiviral drugs could take less dose than EC50 (Table B) while maintaining high efficacy for moderate case. The results from treatments in severe cases **(Figs 9g, 9h, and 9j)** are similar those of moderate cases, and the dose of antiviral treatment could refer to EC50 of single antiviral treatment for moderate patient. On the other hand, the immunological treatment combined with any antiviral treatment did not result obvious improvements compared to the single antiviral treatment in moderate cases **(Figs 9c, 9e, and 9f)**, but combination of the EC50 of ε_4 in severe cases and low doses of other antiviral drugs maintained high levels of treatment efficacy in severe cases **(Figs 9h, 9k, and 9l)**.

Section 8 Influence of other factors on asymptomatic and the incubation period

Except intracellular IFN-I response, we further explored the influence of intercellular T cell response on asymptomatic patient and incubation period. We set five examined groups (2-6 in **Fig E**) and one blank group (1. Mock in **Fig E**). Each group included 200 simulated individuals. Every individual in the mock took parameter values from Table A except $K_1 = 45$. Other five examined groups were corresponding to parameters $[T_0]$ or ρ with different values compared with the mock (**Fig E**).

We quantified the differences between mock and every groups by p-value, medium, mean and coefficient of variation. We noted that changes in the parameter ρ did not

yield significant difference (p>0.05) in comparing with mock, and fluctuations of medium, mean and coefficient of variation were small. Nevertheless, changes in the parameter $[T_0]$ resulted in significant difference (p<0.05), and the fluctuations of medium and mean were large, but the change of coefficient of variation was minor. These results indicated that the number of naïve T cell is closely link to the incubation period in comparing with T cell exhaustion. We further calculated the proportion of asymptomatic patients in every groups then found that asymptomatic probability in groups of T cell exhaustion was nearly the same as the mock, suggesting that large number of naïve T cell could increase the proportion of asymptomatic patients.

Section 9 Model Validation

Intracellular model as we state in the supplementary was established based on previous research to viral triggered type I interferon pathway [7]. As for T cell activation induced by cytokine, we referred to the published work of immune mathematical model [11]. Thus, we need to test the formulation of T cell exhaustion, named as $\delta_{\text{effector}}([\text{Cytokines}])$. Firstly, we proposed a formulation of T cell exhaustion is without accumulation effect of cytokine as follows:

$$\delta_{\text{effector}}([\text{Cytokines}]) = \delta_5 + \rho H(t - t_2) \frac{[\text{Cytokines}]^{m_4}}{[\text{Cytokines}]^{m_4} + K_4^{m_4}}$$

This formulation includes basic apoptosis of effect T cells δ_5, Hill type of T cell exhaustion, and Heaviside function (details stated in Section 2.1: Model description). The formulation fit our data well with appropriate parameter by maximizing the index L and minimizing the index Q in Section 3. Following fitting method in Section 3, all parameters are in Table C. However, the above formula failed to fit our data of effector T cells in critical cases at early infection (**Fig Mc**). We further considered an accumulation effect of cytokine in Section 2.1, and fit the data again using the method in Section 3 (**Fig L**). In Table C, we noted that the accumulation effect could increase the value of the index L in critical cases, and the value of the index Q was slightly increased. The result suggests that the accumulation effect is appropriate for the true dynamics of effector T cell in COVID-19 patients.

Section 10 Supplementary Tables

Table A Parameter values in the proposed model

Parameter	Description	Value	Unit	Source
k_{on}	Association rate between receptor and S protein	0.6759	$\text{nM} \cdot h^{-1}$	[5]
k_{off}	Dissociation rate between receptor and S protein	9.9365	h^{-1}	[5]
k_{in}	Rate of virus release its RNA	0.06	h^{-1}	Estimated
q_0	Virus Budding	0.30	h^{-1}	Estimated
v_{ex}	Local environment volume	1.25	$\times 10^{-11} L^{-1}$	Estimated
v_{cell}	Cell volume	1.00	$\times 10^{-11} L^{-1}$	[16]
β_0	Max infection rate	0.15	h^{-1}	Estimated
η_0	The clear rate of effector T cells	6.75×10^{-8}	$\text{mL cells}^{-1} h^{-1}$	Estimated
ρ	The rate of T cell exhaustion	0.0005	h^{-1}	Estimated
$[T_0]$	Density of naïve T cells	2.0×10^5	cells mL^{-1}	[11,16]
$X_{\text{ex},0}$	Initial virus density	5	nM	Estimated
b_1	The maximal production rate of AVPs on the viruses	4.0	–	Estimated
b_2	The maximal production rate of IFN positive feedback	1.0	$\text{nM} \cdot h^{-1}$	Estimated
α_1	Constant	12.11	–	Estimated
α_2	Constant	9.50	–	Estimated
λ_1	The kinetic rate constant of the viral replication	0.16	h^{-1}	Estimated
λ_2	The activation rate constant of IFNs induced by viruses	0.3	h^{-1}	Estimated
λ_3	The activation rate constant of AVPs induced by IFNs	0.1	h^{-1}	[7]
μ_1	The rate of naïve T cells activated by cytokines	0.9296	h^{-1}	Estimated
μ_2	The rate of cytokines production by infected cells	29.8455	$\text{nM } h^{-1}$	Estimated
μ_3	The rate of cytokines production by effector T cells	6.632×10^{-7}	$\text{nM cells}^{-1} h^{-1}$	Estimated
τ_1	The duration of viral replication	0.5	h	Estimated
τ_2	The duration of viral budding	1.0	h	Estimated
τ_3	the effective time of cytokines	72.0	h	Estimated
K_0	Constant	48.0×10^{-11}	–	Estimated

K_1	IC50 of AVPs on the viruses	47.0	nM	Estimated
K_2	EC50 of IFN positive feedback	0.1	nM	[7]
K_3	EC50 of cytokines activating naïve T cells	40.0	$pg \cdot ml^{-1}$	Estimated
K_4	EC50 of cytokines inducing T cell exhaustion	40.0	$pg \cdot ml^{-1}$	Estimated
δ_1	Degradation of virus	0.1	h^{-1}	[18]
δ_2	Degradation of IFNs	0.4	h^{-1}	[7]
δ_3	Degradation of AVPs	0.12	h^{-1}	[7]
δ_4	Degradation of virus out of cell	0.25	h^{-1}	Estimated
δ_5	The basic death rate of effector cells	0.063	h^{-1}	Estimated
δ_6	The rate of cytokines degradation	0.1733	h^{-1}	Estimated
m_0	Hill coefficient	5	–	Estimated
m_1	Hill coefficient	3	–	Estimated
m_2	Hill coefficient	2	–	Estimated
m_3	Hill coefficient	2	–	Estimated
m_4	Hill coefficient	3	–	Estimated

Table B Parameter of dose response curves of treatment

	ε_1		ε_2		ε_3		ε_4	
	moderate	severe	moderate	severe	moderate	severe	moderate	severe
n	3	4.5	3	11	3.5	12	3.5	6
Λ	0.1904	0.8424	0.0290	0.1126	0.1852	1.24	0.2402	1.748
E^0	0.9567	0.9988	0.9366	0.9813	0.9391	0.9896	0.2728	0.8141
R^2	0.9832	0.9983	0.9795	0.9797	0.9874	0.9494	0.9465	0.9874

Table C Comparison of two indexes between with and without accumulation

	Moderate		Severe		Critical	
	without	with	without	with	without	with
ρ	0.06	0.0005	0.24	0.0025	0.48	0.005
K_4	56	40	56	40	56	40
L	5	5	5	5	3	4
Q	0.134	0.157	0.437	0.629	0.449	0.675

Note: $\mathrm{L} = \sum_{i=1}^{n} \chi(T_{\mathrm{sim}}(t_i))_{(T_{1/4}(t_i), T_{3/4}(t_i))}$, $\mathrm{Q} = \sqrt{\sum_{i=1}^{n} \left(\frac{T_{\mathrm{sim}}(t_i) - T_{1/2}(t_i)}{T_{1/2}(t_i)}\right)^2}$. $T_{\mathrm{sim}}(t_i)$: density of effector T cells from our model. $T_{1/4}(t_i)$, $T_{1/2}(t_i)$ and $T_{3/4}(t_i)$ represent the first, second and third quartile density of effector T cells at time t_i from real data,

respectively. n: total number of real data. $\chi(T_{\mathrm{sim}}(t_i))$ is 1 if $T_{\mathrm{sim}}(t_i)$ is in the interval $(T_{1/4}(t_i), T_{3/4}(t_i))$, otherwise the value of $\chi(T_{\mathrm{sim}}(t_i))$ is zero. Other parameters are default and are shown in Table A.

Section 11 Supporting Figures

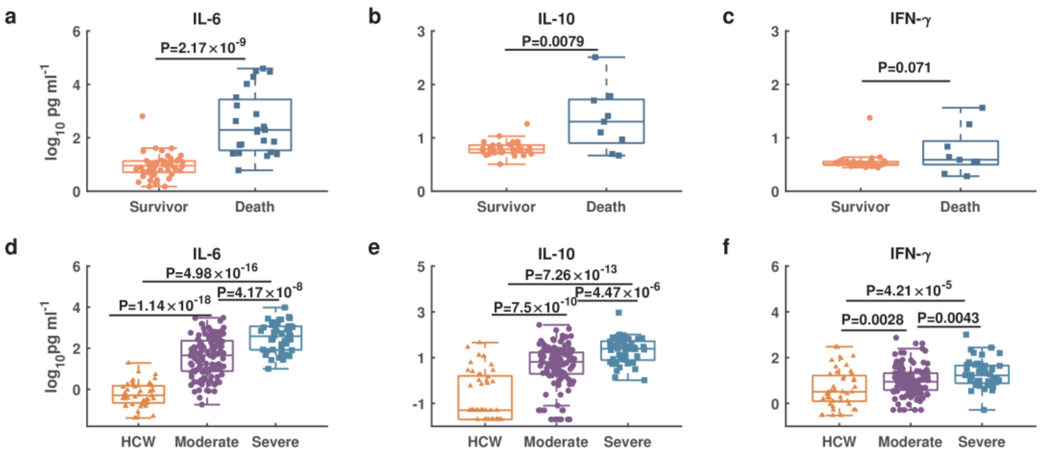

Figure A. Cytokines level from different symptom patients from two different datasets. a-c. IL-6, IL-10 and IFN-γ from survival (n=57) and deceased (n=49) patients from Renmin Hospital of Wuhan University (Dataset 3). **d-f.** IL-6, IL-10 and IFN-γ in health donators (HCW: n=50), moderate (n=117), severe (n=40) from Yale New Haven Hospital (Dataset 4). Significance determined by two-sided, Wilcoxon rank-sum test.

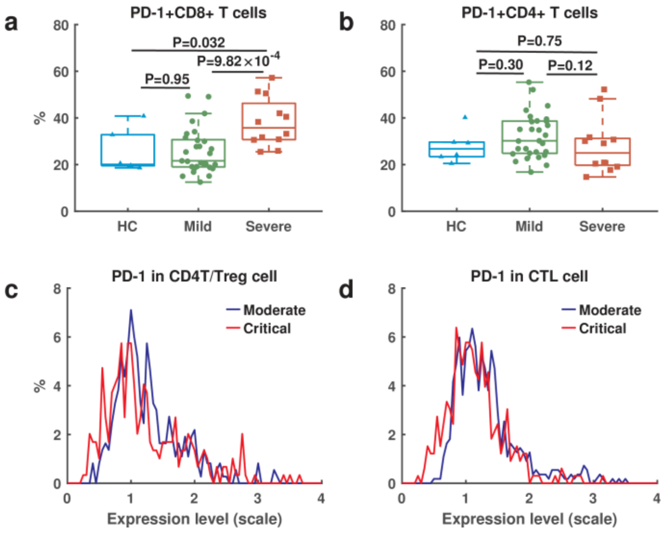

Figure B. The expression level of PD-1 from datasets 2 and 5. a. Percentage of PD-1 expression on $CD8^+$ T cells. **f.** Percentage of PD-1 on $CD4^+$ T cell. **a-b** include health donators (HC: n=6), mild (n=29) and severe (n=12) patients from the Fifth Medical Center of PLA General Hospital of China (Dataset 2). **c.** Distribution of PD-1 expression level in CD4/Treg cells. **d.** Distribution of PD-1 expression level in cytotoxic T lymphocytes (CTL). **c-d** include moderate (n=8) and critical (n=13) patients from Charité-Universitätsmedizin Berlin and University Hospital Leipzig (Dataset 5). Significance determined by two-sided, Wilcoxon rank-sum test.

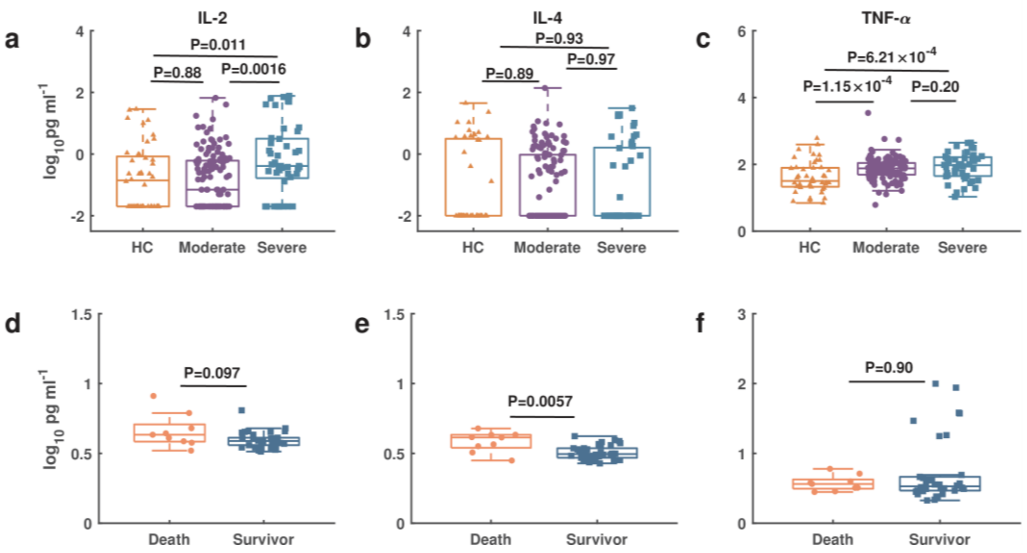

Figure C. The levels of IL-2, IL-4 and TNF-α from different symptom patients from datasets 3 and 4. a-c. IL-2, IL-4 and TNF-α in health control (HC: n=50), moderate (n=117), severe (n=40) from Yale New Haven Hospital (Dataset 4). **d-f.** IL-2, IL-4 and TNF-α from survival (n=57) and deceased (n=49) patients from Renmin Hospital of Wuhan University (Dataset 3). Significance determined by two-sided, Wilcoxon rank-sum test.

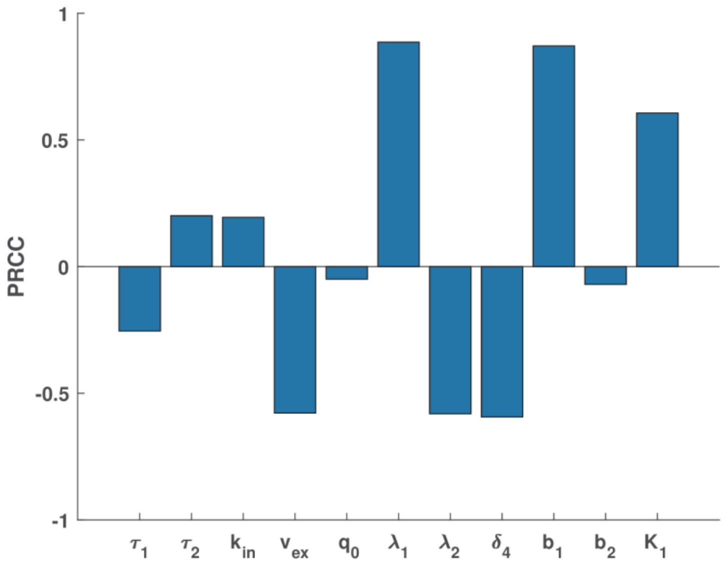

Figure D. Sensitivity analysis to estimated parameters that are related with viral dynamics in the model. Other parameters take default values in Table A.

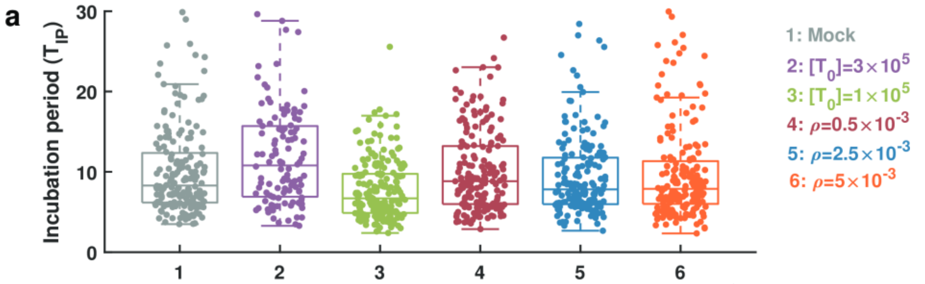

	1	2	3	4	5	6
P-value	1.00	0.0097	1.01×10^{-5}	0.865	0.247	0.225
Median	8.298	10.796	6.697	8.830	7.812	7.890
Mean	10.077	11.783	7.761	10.049	9.385	9.646
Coefficient of variation	0.536	0.5037	0.476	0.498	0.527	0.588

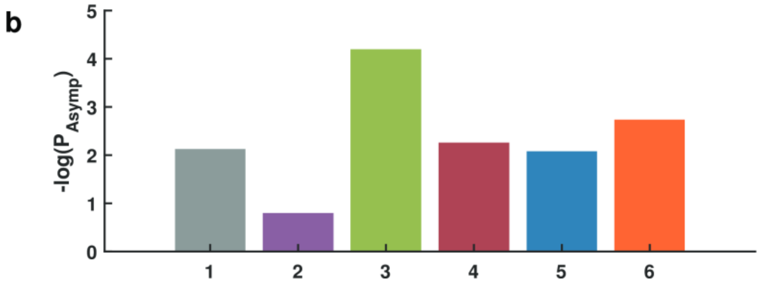

Figure E. Asymptomatic state and incubation period influenced by T cell response. a. Incubation period from five examined groups and one mock. Solid circle represents simulated individual. P-value is calculated between examined group and mock by Wilcoxon test. **b.** The proportion of asymptomatic cases in every group. P_{Asymp} represent the proportion of asymptomatic patients in every groups.

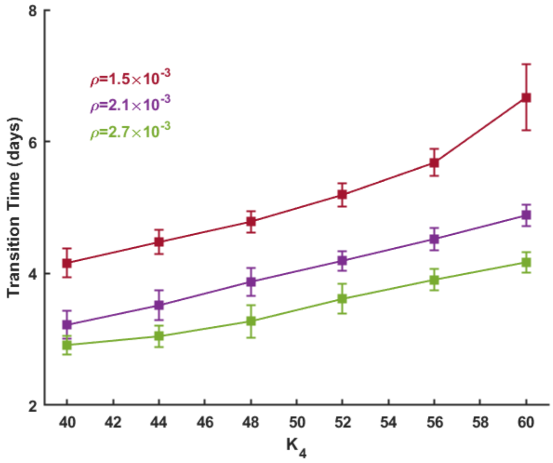

Figure F. Transition time from onset to severe case. Squares and error bars are the mean and standard deviation of the first passage time, respectively.

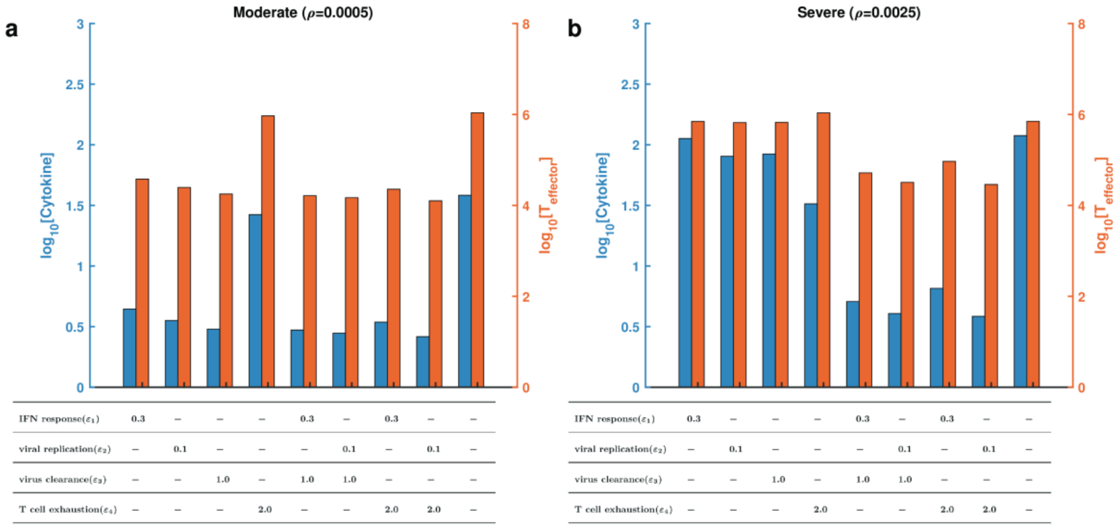

Figure G. The concentration of cytokines and effector T cells at day 15 after treatments. a and b for moderate and severe case respectively.

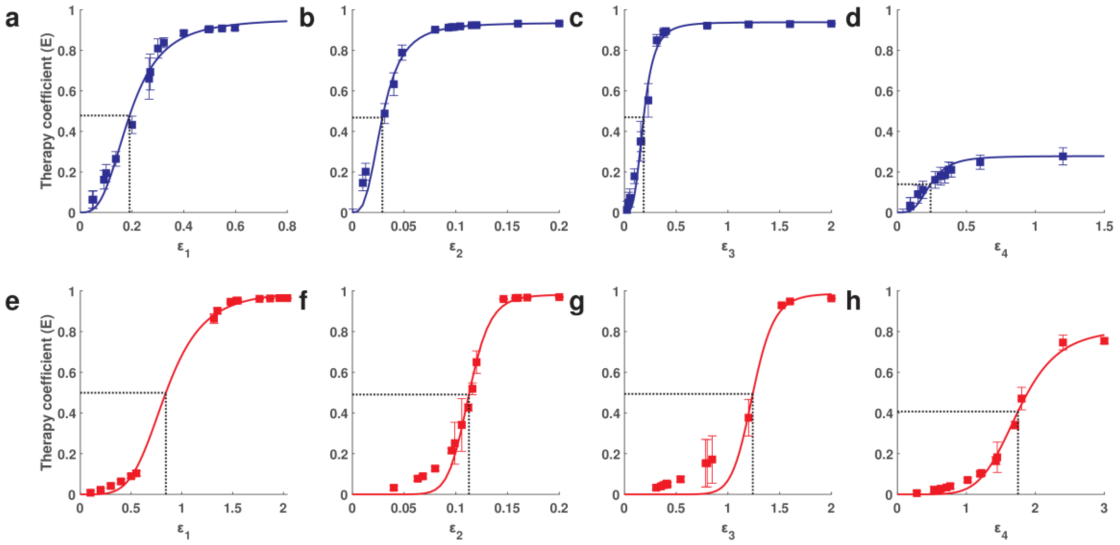

Figure H. Dose response curves of single treatment for moderate and severe case. a-d. Treatment for moderate cases. **e-h.** Treatment for severe cases. Blue and red represent moderate and severe cases, respectively. Squares are mean efficacy for treatment running 50 individuals in the model and the error bars are standard deviation. Lines are dose response curves for every single treatment. Dotted lines are EC50 for every single treatment.

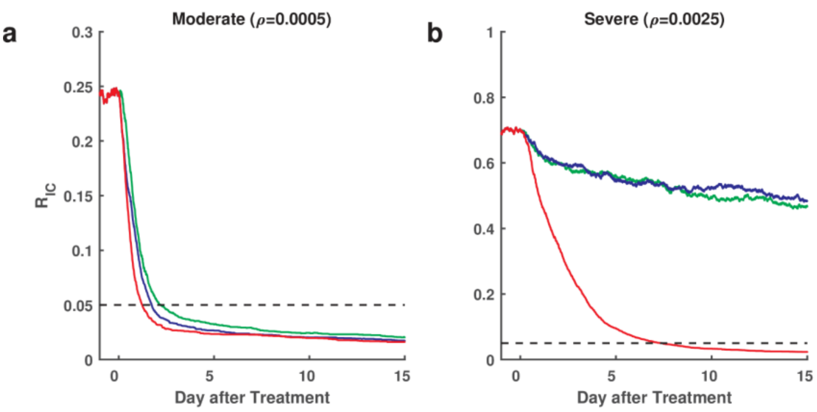

Figure I. Dynamics of antiviral treatments with acceleration of IFN response ($\boldsymbol{\varepsilon_1 = 0.1}$) and promotion of extracellular virus clearance ($\boldsymbol{\varepsilon_3 = 1.0}$) for moderate ($\boldsymbol{\rho = 0.0005}$) and severe ($\boldsymbol{\rho = 0.0025}$) cases. a. Treatment for moderate cases ($\rho = 0.0005$). **b.** Treatment for severe cases ($\rho = 0.0025$). The green, blue and red lines represent acceleration of IFN response ($\varepsilon_1 = 0.1$) alone, single promotion of extracellular virus clearance ($\varepsilon_3 = 1.0$), and combination of the two treatment strategies, respectively. Black dash line is the threshold between asymptomatic and symptomatic state. Other parameters take default values in Table A.

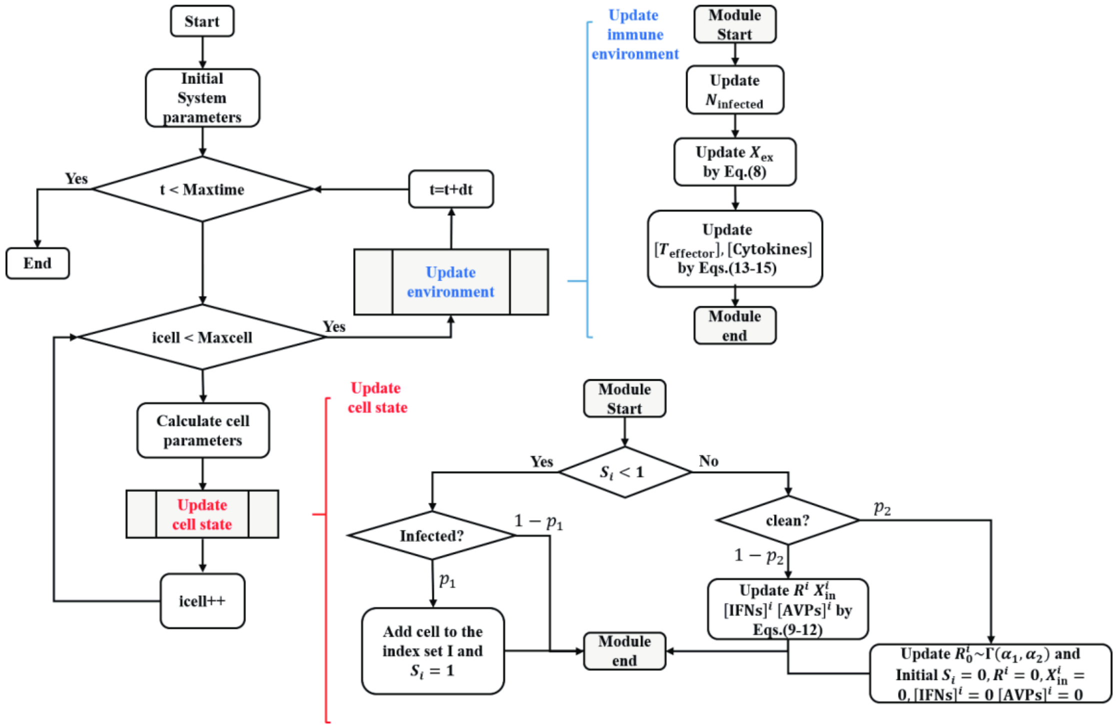

Figure J. Flow chart of the numerical scheme of the simulation process.

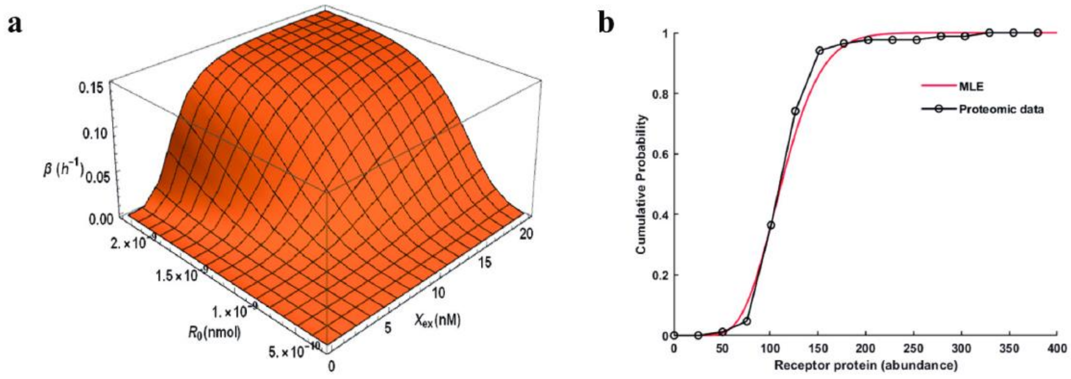

Figure K. Parameter estimation. a. Threshold effect of the infected rate (β) on receptor proteins and extracellular virus concentration. **b.** Comparison of gamma distribution $\Gamma(\alpha_1, \alpha_2)$ and real receptor protein distribution from Dataset 6. MLE refers to maximum likelihood estimation.

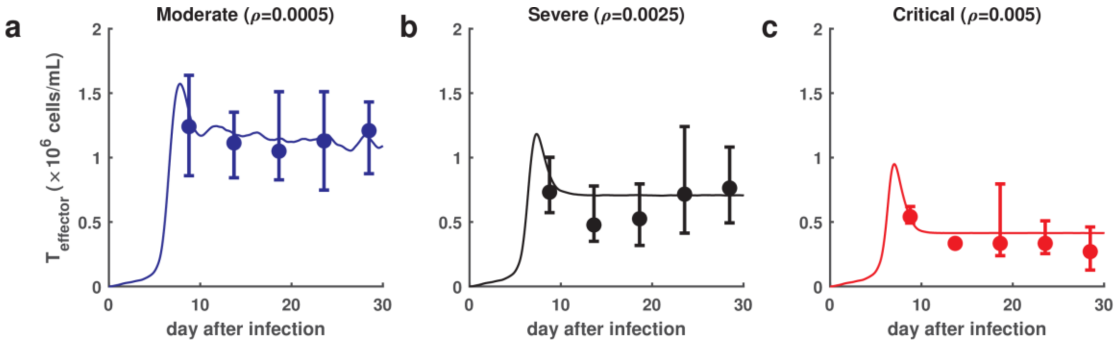

Figure L. T cell dynamic with variable exhausted rate (ρ). a. Moderate: $\rho = 0.0005$; **b.** Severe: $\rho = 0.0025$; **c.** Critical: $\rho = 0.005$. Lines obtained from simulation of model and solid dots are real data from the dataset 7. The blue, black and red solid circles represent for moderate, severe and critical cases, respectively. Other parameters are shown in Table A. Error bars for interquartile range.

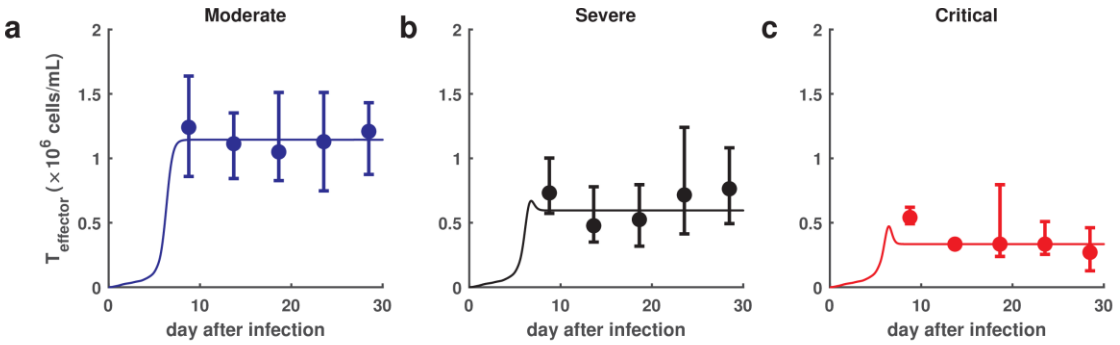

Figure M. T cell dynamics with variable exhausted rate (ρ). a. Moderate; **b.** Severe; **c.** Critical. Lines obtained from simulation of model and solid dots are real data from the dataset 7. The blue, black and red solid circles represent for moderate, severe and critical cases, respectively. The values of parameters K_4 and ρ are shown in Table C and the other parameters are shown in Table A. Error bars for interquartile range.

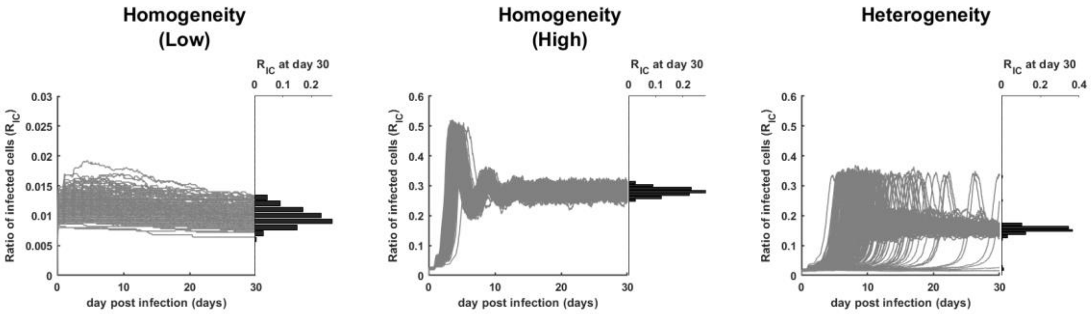

Figure N. Time course of SARS-CoV-2 infection and histogram of R_{IC} at day 30. Homogeneity refers to the same receptor expression on the surface of each target cell. Low homogeneity represents the case in which the value of receptor expression is about the average of gamma distribution. High homogeneity represents the case in which the value of receptor expression is increased by 20% on the basis of the average of gamma distribution. Heterogeneity represents the receptor expression obeys gamma distribution ($R_0 \sim \Gamma(\alpha_1, \alpha_2)$).

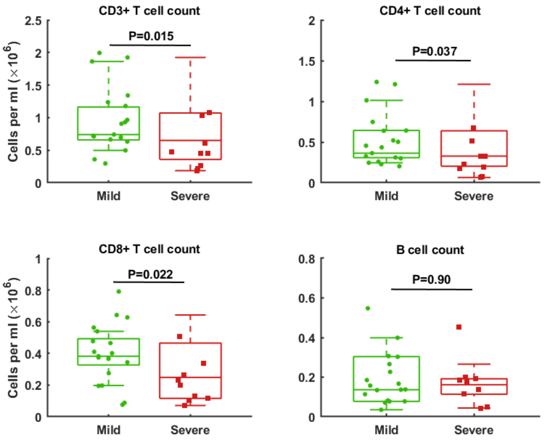

Figure O. The counts of Immune cell from the Dataset 2. Mild (n=29) and severe (n=12) patients from the Fifth Medical Center of PLA General Hospital of China (Dataset 2).

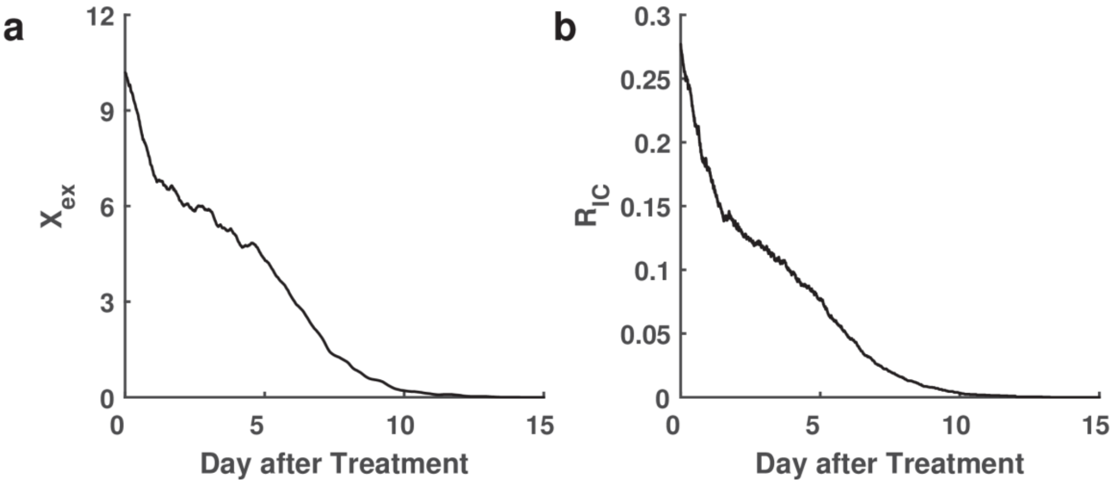

Figure P. Time course of antiviral treatment (ε_5) for moderate patient. a. extracellular virus density (X_{ex}). **b.** ratio of infected cells (R_{IC}). The value of parameters ε_6 and ρ are taken as 0.025 and 0.0005, respectively. The other parameters are default and are shown in Table A.

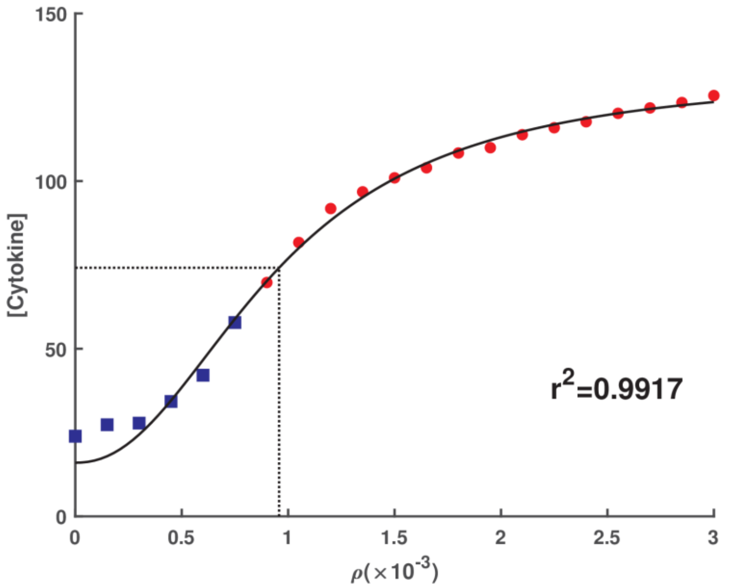

Figure Q. Dose curve of rate of T cell exhaustion (ρ) for cytokines at day 30 after infection ([Ctyokines]). Red solid cycles and blue squares represent severe and mild-moderate cases, respectively. Black line shows the dose response curve of T cell exhaustion for cytokines. The other parameters are default in Table A. The formulation is $[\text{Ctyokines}] = Z_{max}\frac{\rho^n}{\rho^n+\Lambda^n} + Z_0$ where $\Lambda = 0.9576$, $Z_{max} = 116.3$, $Z_0 = 16$, and $n = 2.2$. The value of coefficient of determination (r^2) is 0.9917.

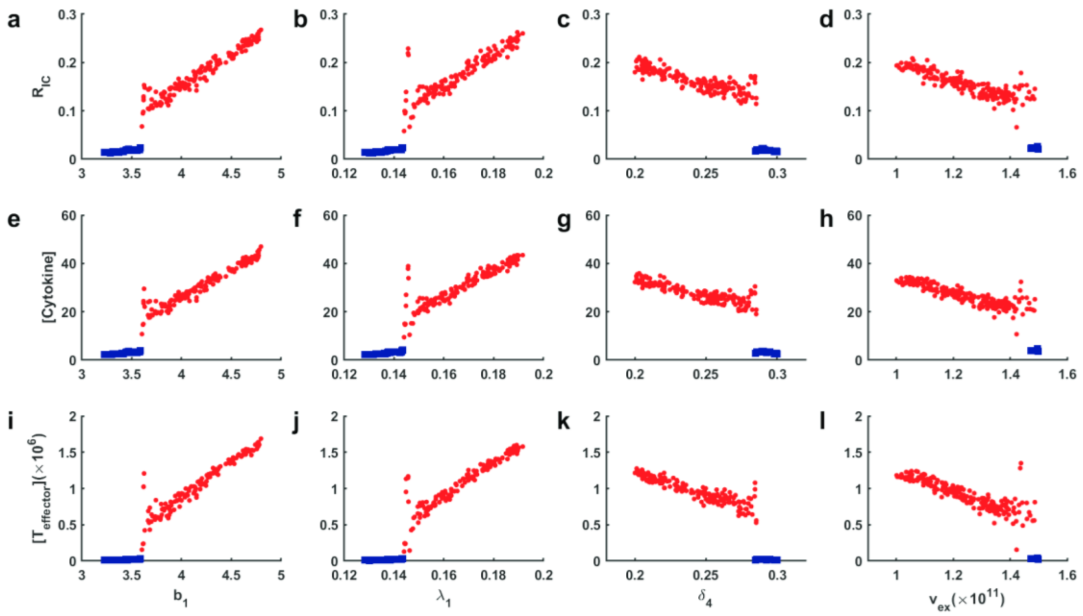

Figure R. Effect of four sensitive parameters on the progression of SARS-CoV-2 infection. (a)-(d) are R_{IC} on day 30. **(e)-(h)** are cytokine concentration ([Cytokine])

on day 30. **(i)-(l)** are number of effector T cells ($[T_{\text{effector}}]$) on day 30. The red solid circles and blue solid squares for symptomatic and asymptomatic patients, respectively. All parameters are assigned with default values in Table A from Supplementary materials.

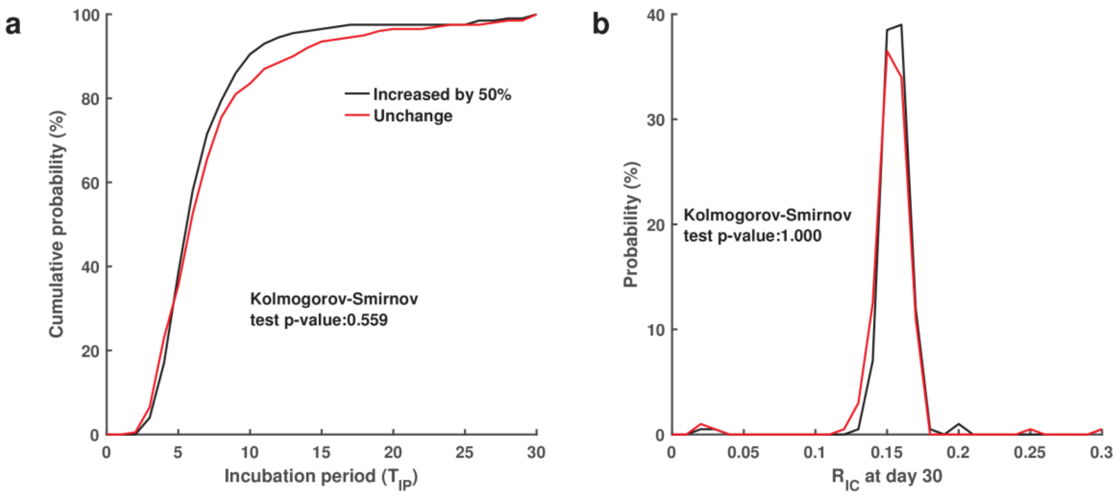

Figure S. Comparison between N increased by 50% and N unchanged. (a) Cumulative probability of incubation period (T_{IP}). **(b)** The distribution of R_{IC} on day 30. The red and black lines represent that N is increased by 50% and that N is unchanged, respectively.

References

1. Diao B, Wang C, Tan Y, Chen X, Liu Y, Ning L, et al. Reduction and Functional Exhaustion of T Cells in Patients With Coronavirus Disease 2019 (COVID-19). Front Immunol. 2020;11: 1–7. doi:10.3389/fimmu.2020.00827
2. Chen Z, John Wherry E. T cell responses in patients with COVID-19. Nat Rev Immunol. 2020;20: 529–536. doi:10.1038/s41577-020-0402-6
3. Pauken KE, Wherry EJ. SnapShot: T Cell Exhaustion. Cell. 2015;163: 1038-1038.e1. doi:10.1016/j.cell.2015.10.054
4. Daly JL, Simonetti B, Antón-Plágaro C, Williamson MK, Shoemark DK, Simón-Gracia L, et al. Neuropilin-1 is a host factor for SARS-CoV-2 infection. bioRxiv. 2020;865: 861–865. doi:10.1126/science.abd3072
5. Wrapp D, Wang N, Corbett KS, Goldsmith JA, Hsieh C-L, Abiona O, et al. Cryo-EM structure of the 2019-nCoV spike in the prefusion conformation. Science (80-). 2020;367: 1260–1263. doi:10.1126/science.abb2507
6. Perelson AS. Modelling viral and immune system dynamics. Nat Rev Immunol. 2002;2: 28–36. doi:10.1038/nri700
7. Tan J, Pan R, Qiao L, Zou X, Pan Z. Modeling and Dynamical Analysis of Virus-Triggered Innate Immune Signaling Pathways. PLoS One. 2012;7. doi:10.1371/journal.pone.0048114
8. Acharya D, Liu GQ, Gack MU. Dysregulation of type I interferon responses in COVID-19. Nat Rev Immunol. 2020;20: 397–398. doi:10.1038/s41577-020-0346-x
9. Pearson JE, Krapivsky P, Perelson AS. Stochastic theory of early viral infection: Continuous versus burst production of virions. PLoS Comput Biol. 2011;7. doi:10.1371/journal.pcbi.1001058
10. Wherry EJ, Kurachi M. Molecular and cellular insights into T cell exhaustion. Nat Rev Immunol. 2015;15: 486–499. doi:10.1038/nri3862
11. Lai X, Stiff A, Duggan M, Wesolowski R, Carson WE, Friedman A. Modeling combination therapy for breast cancer with BET and immune checkpoint inhibitors. Proc Natl Acad Sci U S A. 2018;115: 5534–5539. doi:10.1073/pnas.1721559115
12. Song J-W, Zhang C, Fan X, Meng F-P, Xu Z, Xia P, et al. Immunological and inflammatory profiles in mild and severe cases of COVID-19. Nat Commun. 2020;11: 3410. doi:10.1038/s41467-020-17240-2
13. Pauken KE, Wherry EJ. SnapShot: T Cell Exhaustion. Cell. 2015;163: 1038-1038.e1. doi:10.1016/j.cell.2015.10.054
14. Wherry EJ. T cell exhaustion. Nat Immunol. 2011;12: 492–499. doi:10.1038/ni.2035
15. Lu H. Drug treatment options for the 2019-new coronavirus (2019-nCoV). Biosci Trends. 2020;14: 69–71. doi:10.5582/bst.2020.01020
16. Moran U, Phillips R, Milo R. SnapShot: Key numbers in biology. Cell. 2010;141: 1–2. doi:10.1016/j.cell.2010.06.019
17. Vitale G, van Koetsveld PM, de Herder WW, van der Wansem K, Janssen JAMJL, Colao A, et al. Effects of type I interferons on IGF-mediated

autocrine/paracrine growth of human neuroendocrine tumor cells. Am J Physiol Metab. 2009;296: E559–E566. doi:10.1152/ajpendo.90770.2008
18. Matson MJ, Yinda CK, Seifert SN, Bushmaker T, Fischer RJ, van Doremalen N, et al. Effect of Environmental Conditions on SARS-CoV-2 Stability in Human Nasal Mucus and Sputum. Emerg Infect Dis. 2020;26: 2276–2278. doi:10.3201/eid2609.202267
19. Marino S, Hogue IB, Ray CJ, Kirschner DE. A methodology for performing global uncertainty and sensitivity analysis in systems biology. J Theor Biol. 2008;254: 178–196. doi:10.1016/j.jtbi.2008.04.011
20. Dhouib W, Maatoug J, Ayouni I, Zammit N, Ghammem R, Fredj S Ben, et al. The incubation period during the pandemic of COVID-19: a systematic review and meta-analysis. Syst Rev. 2021;10: 1–14. doi:10.1186/s13643-021-01648-y
21. Felsenstein S, Herbert JA, McNamara PS, Hedrich CM. COVID-19: Immunology and treatment options. Clin Immunol. 2020;215: 108448. doi:10.1016/j.clim.2020.108448
22. Riva G, Nasillo V, Tagliafico E, Trenti T, Luppi M. COVID-19: room for treating T cell exhaustion? Crit Care. 2020;24: 229. doi:10.1186/s13054-020-02960-0
