# Supplementary material for: Data-driven multi-scale mathematical modeling of SARS-CoV-2 infection reveals heterogeneity among COVID-19 patients
Source: PLoS Comput Biol. 2021 Nov 24;17(11):e1009587. doi: 10.1371/journal.pcbi.1009587 (PMC8654229; doi:10.1371/journal.pcbi.1009587)
Supplement: S2 Text — (PDF) [file pcbi.1009587.s002.pdf]

This file includes C++ source codes in "Data-driven multi-scale mathematical modeling of SARS-CoV-2 infection reveals heterogeneity of COVID-19 patients"

Shun Wang[1, 2], Mengqian Hao[1, 2], Zishu Pan[3], Jinzhi Lei[4*] & Xiufen Zou[1, 2*]

The numerical scheme of multiscale model is programmed by C++ and composes of four header files and four CPP files. The compiling file and the running command are shell scripts named as "compile.sh" and "run.sh", respectively. The parameters of this model are saved in "par.dat" and "md.in". Details of all codes are listed below:

**Header file**	**BCTool.h, CCell.h, System.h, Random.h**
**CPP file**	**BCTool.cpp, CCell.cpp, System.cpp, Random.cpp**
**Compiling file**	**compile.sh**
**Running command**	**run.sh**
**Input and parameter file**	**par.dat, md.in**
**Output file**	**md.dat**

"BCTool.h" (Control/main function)

```
#ifndef BCTOOL_H
#define BCTOOL_H

#define Comlength 100
#define Strlength 1024
#define UnitMAX 2147483574
```

```
#define NUMVAR 1
#define MaxCellNumber 100000
#define tau3 7200 //72h
#define tau1 50 //0.5h
#define tau2 100 //1.0h
struct IMD
{
  float dt;// step time
  float T; // simulation time
  float InitialnCoV;// Initial virus concentration
  int ntpx; // control step
  char mdcrd[Comlength];
  char cellpar[Comlength];
  int seed; //Random seed
  int N0; //simulation cell
  double alpha_0; // GammaDistribution shape parameter
  double alpha_1;// GammaDistribution scale parameter
  float xi;// symptom threshold
  double v_cell;// v_cell/v_ex
  double v_ex;// v_ex
  double K3;// EC50 of cytokine
  double K4;// exhuastion
  double mu_1;// cytokine to T cell
  double mu_2;// Infected cell release cytokine
  double mu_3;//  T cell release cytokine
  double d4;// virus clear
  double d5; // T basic death
  double d6; // cytokine degradation
  double T0;// Naive T cell number
  double m3;// Hill coefficient of cytokine
  double m4; // Hill coefficient of exhaustion
  double rho;// T Cell Exhaustion
};
struct Dcell
{
  int type;// 0:unchange
           //1: cell Infected
           //2: cell clear
           //3: release virus
  int _PQ;// Infect:1,unInfect:0
  double R_max; //receptor max number
  double X[5];// X[0]:X_ex;X[1]:R;X[2]:X_in;X[3]:IFNs;X[4]:AVPs
  double V1[tau1];
  double V2[tau2];
```

```
};
struct ApdatedImmune
{
  double Effector_T;
  double Cytokine;
  double CYT[tau3];//accumulation
  int signal;// 0:adaptive immune + innate immune;1: T
exhaustion;2:treatment;
};

#endif
```

"BCTool.cpp" (Control/main function)

```
#include "stdio.h"
#include "stdlib.h"
#include "math.h"
#include "string.h"
#include "time.h"
#include "iostream"

using namespace std;

#include "System.h"
#include "BCTool.h"
#include "Random.h"

struct IMD _MD;
CRandom Rand;

void ReadIPF(char *fn);
void SetParVal(char *str, char const *conststr, char val[]);
void help();
void OutputParameter();
int main(int argc,char *argv[])
{
  System *sys;
        if (argc<2)
        {
                help();
                exit(0);
        }

        ReadIPF(argv[1]);
```

```
        if(_MD.seed > 0) Rand.Initialized(_MD.seed);

    sys = new System(_MD.N0);
    if(sys->Initialized())
    {
       sys->Run();
                              //OutputParameter();
       return(1);
    }
    else
    {
       return(0);
    }
}
void help()
{
        cout<<"Usage: "<<endl;
        cout<<"bct_VirusCell inputfile"<<endl;
        cout<<"inputfile: The name of input file."<<endl;
        cout<<" For example: bct md.in."<<endl;
}
void SetParVal(char *str, char const *conststr, char val[])
{
        char *pst;
        if((pst=strstr(str,conststr))!=NULL)
        {
                strcpy(val,pst+strlen(conststr));
                if((pst = strstr(val,"\""))!=NULL)
                {
                        val[pst - val] = '\0';
                }
        }
        return;
}
void ReadIPF(char *fn)
{
        FILE *fp;
        char str[Strlength], *pst;
        if((fp = fopen(fn,"r"))==NULL)
        {
                cout<<"Cannot open the input file."<<endl;
                exit(0);
        }
        rewind(fp);
```

```
    while(!feof(fp))
    {
            fgets(str,Strlength,fp);
            if(str[0]=='#'){ continue;}
            SetParVal(str, "mdcrd=\"", _MD.mdcrd);
            SetParVal(str, "cellpar=\"",_MD.cellpar);
if((pst=strstr(str,"dt="))!=NULL)
            {
                    _MD.dt=atof(pst+3);
            }
            if((pst=strstr(str,"T="))!=NULL)
            {
                    _MD.T=atof(pst+2);
            }
if((pst=strstr(str,"ntpx="))!=NULL)
            {
  _MD.ntpx=atoi(pst+5);
}
if((pst=strstr(str,"InitialnCoV="))!=NULL)
            {
  _MD.InitialnCoV=atof(pst+12);
}
            if((pst=strstr(str,"seed="))!=NULL)
            {
                    _MD.seed=atoi(pst+5);
            }
if((pst=strstr(str,"N0="))!=NULL)
{
 _MD.N0=atoi(pst+3);
}
            if((pst=strstr(str,"alpha_0="))!=NULL)
            {
                    _MD.alpha_0=atof(pst+8);
            }
            if((pst=strstr(str,"alpha_1="))!=NULL)
            {
                    _MD.alpha_1=atof(pst+8);
            }
            if((pst=strstr(str,"v_cell="))!=NULL)
            {
                    _MD.v_cell=atof(pst+7);
            }
            if((pst=strstr(str,"v_ex="))!=NULL)
            {
```

```
        _MD.v_ex=atof(pst+5);
}
if((pst=strstr(str,"K3="))!=NULL)
{
        _MD.K3=atof(pst+3);
}
if((pst=strstr(str,"K4="))!=NULL)
{
        _MD.K4=atof(pst+3);
}
if((pst=strstr(str,"xi="))!=NULL)
{
        _MD.xi=atof(pst+3);
}
if((pst=strstr(str,"mu_1="))!=NULL)
{
        _MD.mu_1=atof(pst+5);
}
if((pst=strstr(str,"mu_2="))!=NULL)
{
        _MD.mu_2=atof(pst+5);
}
if((pst=strstr(str,"mu_3="))!=NULL)
{
        _MD.mu_3=atof(pst+5);
}
if((pst=strstr(str,"d4="))!=NULL)
{
        _MD.d4=atof(pst+3);
}
if((pst=strstr(str,"d5="))!=NULL)
{
        _MD.d5=atof(pst+3);
}
if((pst=strstr(str,"d6="))!=NULL)
{
        _MD.d6=atof(pst+3);
}
if((pst=strstr(str,"T0="))!=NULL)
{
        _MD.T0=atof(pst+3);
}
if((pst=strstr(str,"m3="))!=NULL)
{
```

```
                _MD.m3=atof(pst+3);
        }
        if((pst=strstr(str,"m4="))!=NULL)
        {
                _MD.m4=atof(pst+3);
        }
        if((pst=strstr(str,"rho="))!=NULL)
        {
                _MD.rho=atof(pst+4);
        }
    }
    fclose(fp);
    return ;
}
void OutputParameter()
{
    printf("%f %f %f %f %f\n",_MD.alpha_0,_MD.alpha_1,_MD.xi,_MD
.v_cell/1e-11,_MD.v_ex/1e-11);
    return ;
}
```

“CCell.h” (Intracellular model of a single cell)

```
#ifndef CELL_H
#define CELL_H

#include "BCTool.h"
#define tau1 50 // virus replication tau1=0.5h
#define tau2 100 // virus budding tau1=1.0h
struct Cellpara
{
  double beta0,K0,q0;
  double k_in,k_on,k_off;
  double lambda1,lambda2,lambda3;
  double b1,b2;
  double d1,d2,d3;
  double m0,m1,m2;
  double K1,K2;
  double eta0;
};
class CCell
{
private:
```

```
  int _cellid;
  double _R;// max receptor number
  int _PQ;// _PQ=1 cell is infected;_PQ=0 cell is not infected
  double X[5];// X[0]:X_ex;X[1]:R;X[2]:X_in;X[3]:IFNs;X[4]:AVPs
  double V1[tau1];// virus replcation tau1=0.5h
  double V2[tau2];// virus budding tau2=1.0h
  Cellpara _par;
public:
  CCell();
  ~CCell();
  Dcell CellfateDesion(float dt,double T1);
  bool UpdateX_in(double t);
  bool Initialized(int k,char fn[]);
  double VirusInitalized(int k);
  void ReadPar(char fpar[]);
  void Outputparameter();
  double fInfectedrate(float virus,float R_max,float dt);
  double fdeathrate(double T1,float dt);
  void UpdateResevior();
  friend class System;
};


#endif
```

"CCell.cpp" (Intracellular model of a single cell)

```
#include "stdio.h"
#include "stdlib.h"
#include "math.h"
#include "string.h"
#include "time.h"
#include "iostream"

using namespace std;

#include "CCell.h"
#include "System.h"
#include "BCTool.h"
#include "Random.h"

extern CRandom Rand;
extern struct IMD _MD;
CCell::CCell()
```

```
{
}
CCell::~CCell()
{
}
void CCell::UpdateResevior()
{
  double *K11,*K21;
  //tau1
  K11=new double [tau1];
  for(int i=0;i<(tau1-1);i++)
  {
    K11[i]=V1[i+1];
  }
  K11[tau1-1]=X[2];// X_in
  for (int i = 0; i < tau1; i++)
  {
    V1[i]=K11[i];
  }
  delete K11;
  // tau2
  K21=new double [tau2];
  for (int i = 0; i < (tau2-1); i++)
  {
    K21[i]=V2[i+1];
  }
  K21[tau2-1]=X[2]; //X_in
  for(int i=0;i<tau2;i++)
  {
    V2[i]=K21[i];
  }
  delete K21;
  return ;
}
bool CCell::UpdateX_in(double dt)// Update intracelluar virus
{
  double *y,*yc,*yp;
  y=new double [5];yc=new double [5];yp=new double [5];
  for(int i=0;i<5;i++){y[i]=X[i];}

  yp[0]=y[0];
  yp[1]=y[1]+dt*(_par.k_on*(_R-y[1])*y[0]-_par.k_off*y[1]-
_par.k_in*y[1]);
```

```
yp[2]=y[2]+dt*(_par.k_in*y[1]/(_MD.v_cell)+_par.lambda1*V1[0]*_par.b
1*pow(_par.K1,_par.m1)/(pow(_par.K1,_par.m1)+pow(y[4],_par.m1))-
_par.q0*V2[0]-_par.d1*y[2]);

yp[3]=y[3]+dt*(_par.lambda2*y[2]+_par.b2*pow(y[3],_par.m2)/(pow(_par
.K2,_par.m2)+pow(y[3],_par.m2))-_par.d2*y[3]);
  yp[4]=y[4]+dt*(_par.lambda3*y[3]-_par.d3*y[4]);

  yc[0]=y[0];
  yc[1]=y[1]+dt*(_par.k_on*(_R-yp[1])*yp[0]-_par.k_off*yp[1]-
_par.k_in*yp[1]);

yc[2]=y[2]+dt*(_par.k_in*yp[1]/(_MD.v_cell)+_par.lambda1*V1[1]*_par.
b1*pow(_par.K1,_par.m1)/(pow(_par.K1,_par.m1)+pow(yp[4],_par.m1))-
_par.q0*V2[1]-_par.d1*yp[2]);

yc[3]=y[3]+dt*(_par.lambda2*yp[2]+_par.b2*pow(yp[3],_par.m2)/(pow(_p
ar.K2,_par.m2)+pow(yp[3],_par.m2))-_par.d2*yp[3]);
  yc[4]=y[4]+dt*(_par.lambda3*yp[3]-_par.d3*yp[4]);

  for(int i=0;i<5;i++){X[i]=0.5*(yc[i]+yp[i]);}
  UpdateResevior();// Update resevior
  delete y;
  delete yc;
  delete yp;
  return 1;
}
bool CCell::Initialized(int k,char fn[])
{
  ReadPar(fn);
  _cellid=k;
  _PQ=0;
  _R=Rand.GammaDistribution(_MD.alpha_0,_MD.alpha_1)*pow(10,-11);//
*10^-11 nmol
  X[0]=_MD.InitialnCoV;
  X[1]=0; // initial receptpr binded with virus =0.0
  X[2]=0; // initial viral RNA=0.0
  X[3]=0.0; //initial [IFNs]=0.0
  X[4]=0.0; // initial [AVPs]=0.0
  memset(V1,0,sizeof(V1));
  memset(V2,0,sizeof(V2));
  return true;
}
Dcell CCell::CellfateDesion(float dt,double T1)
```

```
{
  double beta,eta;
  double _rand;
  _rand=Rand.Value(0,1);
  Dcell nextcell;
  beta=fInfectedrate(X[0],_R,dt);
  eta=fdeathrate(T1,dt);
  nextcell.R_max=_R;
  nextcell._PQ=_PQ;
  nextcell.X[0]=X[0];
  if(_PQ==0)
  {
    if(_rand<beta)// cell is infected
    {
      nextcell.type=1;// infected cell
      nextcell._PQ=1;
      for(int i=1;i<5;i++){nextcell.X[i]=X[i];}
      for(int i=0;i<tau1;i++){nextcell.V1[i]=V1[i];} //resevior
      for(int i=0;i<tau2;i++){nextcell.V2[i]=V2[i];}
    }
    else // cell is normal
    {
      nextcell.type=0;
      nextcell._PQ=0;
      for(int i=1;i<5;i++){nextcell.X[i]=X[i];}
      for(int i=0;i<tau1;i++){nextcell.V1[i]=V1[i];} //resevior
      for(int i=0;i<tau2;i++){nextcell.V2[i]=V2[i];}
    }
  }
  else if(_PQ==1)
  {
    if((_rand<eta))// infected cell is cleared
    {
     nextcell.type=2; //cell clear
     nextcell._PQ=0;

nextcell.R_max=Rand.GammaDistribution(_MD.alpha_0,_MD.alpha_1)*pow(1
0,-11);// *10^-11 nmol
     nextcell.X[1]=0; // R^i=0
     nextcell.X[2]=0; // Viral RNA=0
     nextcell.X[3]=0.0;// IFNs=0.0nM
     nextcell.X[4]=0.0; // AVPs=0.0nM
     for(int i=0;i<tau1;i++){nextcell.V1[i]=0;} //resevior
     for(int i=0;i<tau2;i++){nextcell.V2[i]=0;}
```

```
    }
    else if(UpdateX_in(dt))
    {
      nextcell.type=3;// release virus
      nextcell._PQ=1;
      for(int i=1;i<5;i++){nextcell.X[i]=X[i];}
      for(int i=0;i<tau1;i++){nextcell.V1[i]=V1[i];} //resevior
      for(int i=0;i<tau2;i++){nextcell.V2[i]=V2[i];}
    }
  }
  return (nextcell);
}

double CCell::fInfectedrate(float virus,float R_max,float dt)
{
  double beta,theta,A;
  A=_par.k_off/_par.k_on;

theta=pow(virus*R_max,_par.m0)/(pow(virus*R_max,_par.m0)+pow(_par.K0
*(A+virus),_par.m0));
  beta=_par.beta0*theta*dt;
  return (beta);
}
double CCell::fdeathrate(double T1,float dt)
{
  double eta;
  eta=_par.eta0*T1*dt;// abnormal cell is cleared
  return eta;
}

void CCell::ReadPar(char fpar[])
{
  FILE *fp;
  char str[Strlength],*pst;
  if((fp=fopen(fpar,"r"))==NULL)
  {
    cout<<"Cannot open the cell parameter file"<<endl;
    exit(0);
  }
  rewind(fp);
  while(!feof(fp))
  {
    fgets(str,Strlength,fp);
    if(str[0]=='#'){continue;}
```

```
if((pst=strstr(str,"lambda1="))!=NULL)
{
  _par.lambda1=atof(pst+8);
}
if((pst=strstr(str,"lambda2="))!=NULL)
{
  _par.lambda2=atof(pst+8);
}
if((pst=strstr(str,"lambda3="))!=NULL)
{
  _par.lambda3=atof(pst+8);
}
if((pst=strstr(str,"K0="))!=NULL)
{
  _par.K0=atof(pst+3);
}
if((pst=strstr(str,"K1="))!=NULL)
{
  _par.K1=atof(pst+3);
}
if((pst=strstr(str,"K2="))!=NULL)
{
  _par.K2=atof(pst+3);
}
if((pst=strstr(str,"k_in="))!=NULL)
{
  _par.k_in=atof(pst+5);
}
if((pst=strstr(str,"k_on="))!=NULL)
{
  _par.k_on=atof(pst+5);
}
if((pst=strstr(str,"k_off="))!=NULL)
{
  _par.k_off=atof(pst+6);
}
if((pst=strstr(str,"b1="))!=NULL)
{
  _par.b1=atof(pst+3);
}
if((pst=strstr(str,"b2="))!=NULL)
{
  _par.b2=atof(pst+3);
```

```
    }
    if((pst=strstr(str,"beta0="))!=NULL)
    {
      _par.beta0=atof(pst+6);
    }
    if((pst=strstr(str,"eta0="))!=NULL)
    {
      _par.eta0=atof(pst+5);
    }
    if((pst=strstr(str,"d1="))!=NULL)
    {
      _par.d1=atof(pst+3);
    }
    if((pst=strstr(str,"d2="))!=NULL)
    {
      _par.d2=atof(pst+3);
    }
    if((pst=strstr(str,"d3="))!=NULL)
    {
      _par.d3=atof(pst+3);
    }
    if((pst=strstr(str,"q0="))!=NULL)
    {
      _par.q0=atof(pst+3);
    }
    if((pst=strstr(str,"m0="))!=NULL)
    {
      _par.m0=atof(pst+3);
    }
    if((pst=strstr(str,"m1="))!=NULL)
    {
      _par.m1=atof(pst+3);
    }
    if((pst=strstr(str,"m2="))!=NULL)
    {
      _par.m2=atof(pst+3);
    }
  }
   fclose(fp);
}
void CCell::Outputparameter()
{
  printf("k_in=%f k_off=%f
k_on=%f\n",_par.k_in,_par.k_off,_par.k_on);
```

```
  printf("lambda1=%f lambda2=%f
lambda3=%f\n",_par.lambda1,_par.lambda2,_par.lambda3);
  printf("K1=%f K2=%f q0=%f\n",_par.K1,_par.K2,_par.q0);
  printf("beta0=%f K0=%f \n",_par.beta0,_par.K0);
  printf("d1=%f d2=%f d3=%f\n",_par.d1,_par.d2,_par.d3);
  printf("m1=%f m2=%f\n",_par.m1,_par.m2);
  printf("b1=%f b2=%f eta0=%f\n",_par.b1,_par.b2,_par.eta0);
}
```

"System.h"(Intercellular model of immunity and virus)

```
#ifndef SYSTEM_H
#define SYSTEM_H
#include "CCell.h"
#include "BCTool.h"


class System
{
private:
  int NumPoolCell; //simulation cell number
  double nCoV;// virus concentration
  double t1;// time of symptom presentation
  double omega;//the beginning of T exhaustion
  double omega1;//the end of T exhaustion
  int _N0,_N1,_N2,_N3;//_N0:normal cell;_N1:infected
cell;_N2:cleared cell;_N3:cell releasing virus
  CCell *_cell;
  ApdatedImmune AI; //Adapated Immune to virus
public:
  System();
  ~System();
  System(int N0); //Initialized system
  CCell& operator()(int indx); // Edit to return the type of your
own class.
  bool Initialized();// Initialize parameter
  void OutPutCells(double t); // output cell result
        void OutPutSys(int step); // output system result
        void Run();
  void UpdateX_ex(double dt);// Update X_ex
  Dcell nextcell;
  bool SystemUpdate(double dt);// Update system
  ApdatedImmune Update_ApdatImmune(int N0,double dt);// Update
IL6,activated T cell
```

```
  double delta_exhaust(int signal);// exhaustion rate
  ApdatedImmune Accumulate_CYT(int signal,int length);// CYT(tau)
  double NumerialIntegrate(int length); //
  friend class CCell;

};




#endif
```

"System.cpp" (Intercellular model of immunity and virus)

```
#include "stdio.h"
#include "stdlib.h"
#include "math.h"
#include "string.h"
#include "time.h"
#include "iostream"
using namespace std;

#include "BCTool.h"
#include "CCell.h"
#include "Random.h"
#include "System.h"

extern struct IMD _MD;
extern CRandom Rand;
System::System()
{
}

System::~System()
{
}
System::System(int N0)
{
  NumPoolCell=N0;
  nCoV=_MD.InitialnCoV;
  _cell=new CCell[MaxCellNumber];
  AI.Cytokine=0;
  AI.Effector_T=0;
  AI.signal=0;
```

```
  memset(AI.CYT,0,sizeof(AI.CYT));
}
bool System::Initialized()
{
  int k;
  k=1;
  do
  {
    if((*this)(k).Initialized(k,_MD.cellpar))
    {
      k++;
    }
    else
    {
    }
  } while (k<=MaxCellNumber);
  return (1);
}
void System::Run()
{
  double t;
  int step;
  int k;
  FILE *fmdsysm;
  char fn[Comlength];
  sprintf(fn,"%s.dat",_MD.mdcrd);
  if((fmdsysm=fopen(fn,"w"))==NULL)
  {
    cout<<"Cannot open the input file"<<endl;
    exit(0);
  }
  step=0;omega1=720.0;t1=-1; //30 day=720h t1:characterize
incubation
  //omega=Rand.Value(120.0,240.0);// period after T exhaustion 5~10
day
  OutPutSys(step);
  _N0=NumPoolCell;_N1=0;_N2=0;_N3=0;
  for(t=0;t<=_MD.T;t=t+_MD.dt)
  {
    step=step+1;

fprintf(fmdsysm,"%6.4f %6.4f %6.4f %6.4f %d %d %d %d\n",t,nCoV,AI.Cy
tokine,AI.Effector_T/pow(10,5),_N0,_N1,_N2,_N3); //output system
result
```

```
    if(SystemUpdate(_MD.dt))
    {
      Accumulate_CYT(AI.signal,tau3);// accumulate cytokine effect
      UpdateX_ex(_MD.dt); // Update X_ex
      Update_ApdatImmune(_N3,_MD.dt); // Update immune environment
      nCoV=(*this)(1).X[0];// number of cell from 1 to NumberPoolcell
      if((_MD.ntpx>0)&&(step%_MD.ntpx==0))
      {
        OutPutSys(step);// output intracelluar virus

//fprintf(fmdsysm,"%f %f %f %f %d %d %d\n",t,nCoV,AI.IL6,AI.T1/10000
0.0,_N0,_N1,_N2); //output system result
      }
      if(nCoV<0){break;}

      if(AI.signal==0&&t1>0)
      {
        AI.signal=1;// begining of T exhaustion
      }
      else if(AI.signal==0) //xi symptom presentation
      {
       if(_N3>=(int)NumPoolCell*_MD.xi)
       {
         t1=t;
       }
      }
      else if(AI.signal==1)
      {
       if((step*_MD.dt)>omega1)
       {
        AI.signal=2;// begining of treatment
       }
      }
    }
  }
  fclose(fmdsysm);
  return ;
}

bool System::SystemUpdate(double dt)
{
  int k;
  int *PQ;
  double *R;// receptor
```

```
  double *Y1; // X_ex
  double *Y2; //R
  double *Y3;//X_in
  double *Y4;// IFNs
  double *Y5;// AVPs

  int Ntemp;

  R=new double[NumPoolCell+1];
  PQ=new int[NumPoolCell+1];
  Y1=new double[NumPoolCell+1];
  Y2=new double [NumPoolCell+1];
  Y3=new double [NumPoolCell+1];
  Y4=new double [NumPoolCell+1];
  Y5=new double [NumPoolCell+1];

  double (*Y6)[tau1]=new double [NumPoolCell+1][tau1];//
(*this)(k).V1
  double (*Y7)[tau2]=new double [NumPoolCell+1][tau2]; //
(*this)(k).V2
  Ntemp=0;
  _N0=0; // normal cell
  _N1=0; // infected cell
  _N2=0;// cleared cell
  _N3=0;// release virus
  for(k=1;k<=NumPoolCell;k++)
  {
    nextcell=(*this)(k).CellfateDesion(dt,AI.Effector_T);
    switch(nextcell.type)
    {
      case 0: //normal cell
        Ntemp++;
        _N0++;
        break;
      case 1: //cell is infected
        _N1++;
        Ntemp++;
        break;
      case 2: // cell clear and replaced new normal cell
        Ntemp++;
        _N2++;
        break;
      case 3: // cell release virus
        Ntemp++;
```

```
      _N3++;
      break;
    }
    R[Ntemp]=nextcell.R_max;
    PQ[Ntemp]=nextcell._PQ;
    Y1[Ntemp]=nextcell.X[0];
    Y2[Ntemp]=nextcell.X[1];
    Y3[Ntemp]=nextcell.X[2];
    Y4[Ntemp]=nextcell.X[3];
    Y5[Ntemp]=nextcell.X[4];
    for(int i=0;i<tau1;i++){Y6[Ntemp][i]=nextcell.V1[i];}
    for(int j=0;j<tau2;j++){Y7[Ntemp][j]=nextcell.V2[j];}
  }
  for(k=1;k<=Ntemp;k++)
  {
    (*this)(k)._PQ=PQ[k];
    (*this)(k)._R=R[k];
    (*this)(k).X[0]=Y1[k];
    (*this)(k).X[1]=Y2[k];
    (*this)(k).X[2]=Y3[k];
    (*this)(k).X[3]=Y4[k];
    (*this)(k).X[4]=Y5[k];
    for(int i=0;i<tau1;i++){(*this)(k).V1[i]=Y6[k][i];}
    for(int j=0;j<tau2;j++){(*this)(k).V2[j]=Y7[k][j];}
  }

  delete R;
  delete PQ;

  delete Y1;
  delete Y2;
  delete Y3;
  delete Y4;
  delete Y5;
  delete Y6;
  delete Y7;
  return 1;
}
void System::OutPutSys(int step)
{
  FILE *fp;
  int k;
  char fnc[Strlength];
  sprintf(fnc,"%s-%d.dat",_MD.mdcrd,step);
```

```
  if((fp=fopen(fnc,"w"))==NULL)
  {
    cout<<"Cannot open the fmdy file"<<endl;
    exit(0);
  }
  for(int k=1;k<=NumPoolCell;k++)
  {

fprintf(fp,"%d %d %6.3f %6.3f %6.3f %6.3f %6.3f %6.3f\n",k,(*this)(k
)._PQ,(*this)(k)._R/pow(10,-
11),(*this)(k).X[0],(*this)(k).X[1]/pow(10,-
11),(*this)(k).X[2],(*this)(k).X[3],(*this)(k).X[4]);
  }
  fclose(fp);
}
void  System::UpdateX_ex(double dt)// DDE23
{
  double *y,*yp,*yc;
  double kon,koff,q0;
  y=new double [1];yp=new double [1];yc=new double [1];
  y[0]=(*this)(1).X[0];// X_ex

kon=(*this)(1)._par.k_on;koff=(*this)(1)._par.k_off;q0=(*this)(1)._p
ar.q0;// par kon koff q0
  double
sum1_1,sum2_1,sum3_1,sum1_2,sum2_2,sum3_2;//sum1:q(X^i_in),sum3:k_of
f*R^i;sum2:kon*(R_max-R^i)
  sum1_1=0;sum2_1=0;sum3_1=0;
  for(int k=1;k<=NumPoolCell;k++)
  {
    if((*this)(k)._PQ==1)
    {
      sum1_1=sum1_1+q0*(*this)(k).V2[0];
      sum2_1=sum2_1+kon*((*this)(k)._R-(*this)(k).X[1])*y[0];
      sum3_1=sum3_1+koff*(*this)(k).X[1];
    }
  }

yp[0]=y[0]+dt*(_MD.v_cell*sum1_1*1.0/(_MD.N0*_MD.v_ex)+sum3_1*1.0/(_
MD.v_ex*_MD.N0)-sum2_1*1.0/(_MD.v_ex*_MD.N0)-_MD.d4*y[0]);

  sum1_2=0;sum2_2=0;sum3_2=0;
  for(int k=1;k<=NumPoolCell;k++)
```

```
  {
    if((*this)(k)._PQ==1)
    {
      sum1_2=sum1_2+q0*(*this)(k).V2[1];
      sum2_2=sum2_2+kon*((*this)(k)._R-(*this)(k).X[1])*yp[0];
      sum3_2=sum3_2+koff*(*this)(k).X[1];
    }
  }

yc[0]=y[0]+dt*(_MD.v_cell*sum1_2*1.0/(_MD.N0*_MD.v_ex)+sum3_2*1.0/(_
MD.v_ex*_MD.N0)-sum2_2*1.0/(_MD.v_ex*_MD.N0)-_MD.d4*yp[0]);


  for(int k=1;k<=NumPoolCell;k++)
  {
    (*this)(k).X[0]=0.5*(yc[0]+yp[0]);
  }

//PureEx=dt*(_MD.gamma_1*sum1_2*1.0/_MD.N0+_MD.gamma_1*sum1_1*1.0/_M
D.N0);
  delete y;
  delete yp;
  delete yc;
  return ;
}
ApdatedImmune System::Update_ApdatImmune(int N00,double dt) //ODE23
{
  double *y,*yp,*yc;
  double mu1,mu2,mu3,delta1,delta2;
  delta1=delta_exhaust(AI.signal);
  mu1=_MD.mu_1,mu2=_MD.mu_2,mu3=_MD.mu_3,delta2=_MD.d6;
  y=new double [2];yp=new double [2];yc=new double [2];
  y[1]=AI.Cytokine;y[0]=AI.Effector_T;


yp[0]=y[0]+dt*(mu1*_MD.T0*pow(y[1],_MD.m3)/(pow(y[1],_MD.m3)+pow(_MD
.K3,_MD.m3))-delta1*y[0]);
  yp[1]=y[1]+dt*(mu2*N00*1.0/NumPoolCell+mu3*y[1]-delta2*y[1]);


yc[0]=y[0]+dt*(mu1*_MD.T0*pow(yp[1],_MD.m3)/(pow(yp[1],_MD.m3)+pow(_
MD.K3,_MD.m3))-delta1*yp[0]);
  yc[1]=y[1]+dt*(mu2*N00*1.0/NumPoolCell+mu3*yp[1]-delta2*yp[1]);
```

```
  AI.Effector_T=0.5*(yc[0]+yp[0]);
  AI.Cytokine=0.5*(yc[1]+yp[1]);
  delete y;
  delete yp;
  delete yc;
  return AI;
}

double System::delta_exhaust(int signal)// T cell exhaustion
{
  double delta,T_exhaust;
  if (signal>0) // syptom presentation
  {
    T_exhaust=NumerialIntegrate(tau3);
    delta=_MD.d5+_MD.rho*T_exhaust;
  }
  else
  {
    delta=_MD.d5;
  }
  return delta;
}
double System::NumerialIntegrate(int length)//tau3=7200*dt=72hr,
{
  double result,*Y;
  Y=new double [length];
  for(int i=0;i<length;i++)
  {

Y[i]=pow(AI.CYT[i],_MD.m4)/(pow(_MD.K4,_MD.m4)+pow(AI.CYT[i],_MD.m4)
);
  }
  result=_MD.dt*(Y[0]+Y[length-1])/2;
  for (int i=1;i<length-1;i++){result+=Y[i]*_MD.dt;}
  delete Y;
  return result;
}
ApdatedImmune System ::Accumulate_CYT(int signal,int length) //
Accumulating Cytokine
{
  if(signal>=0)
  {
    double *K;
    K=new double[length];
```

```
    for (int i=0;i<length-1;i++)
    {
       K[i]=AI.CYT[i+1];
    }// replace CTY[1]~CTY[length-2]
    K[length-1]=AI.Cytokine;
    for(int i=0;i<length;i++)
    {
      AI.CYT[i]=K[i];
    }
    delete K;
  }
  return AI;
}
CCell&System ::operator()(int index)
{
  if(index>=0&&index<=MaxCellNumber)
  {
    return *(_cell+(index-1));
  }
  else
  {
    cout<<"Err<< CSystem () >>Dimensional error"<<endl;
          exit(0);
  }

}
```

"Random.h" (Random number)

```
#ifndef RANDOM_H
#define RANDOM_H

//#define RANDMAX 23371.0
//#define RANDPI 3.1415926

class CRandom{
private:

        double x[97];
    double c;
public:
```

```
        CRandom();
    CRandom(unsigned seed);
        double Value();                             // Get the value
with uniform distribution;
        double NormalDistribution();      // Get the value with
standard normal distribution;
        double NormalDistribution(double mu, double sigma);  // Get
the value with normal distribution with mean mu and standard
deviation sigma
    int BinomialDistribution(int n, double p);      // Get a random
number with binormal distribution B(n,p);
    double BetaDistribution(double a, double b);    // Get a random
number with BetaDistribution
    double GammaDistribution(double a, double b);   // Get a random
number with GammaDistribution
        void Initialized(unsigned seed);    // Initialzation of the
random number
        int NegativeBinomialDistribution(int n,double p); // Get a
random number with negative binomial distribution NB(n,p)
        double operator()();                 // Get a random value;
        double Value(double a, double b);
        double operator()(double a, double b);
    double WienerGen();                  // Generate a Wiener
process;
};

#endif
```

"Random.cpp" (Random number)

```
#include "Random.h"

#define RANDMAX 23371.0
#define RANDPI 3.1415926

#include "stdio.h"
#include "stdlib.h"
#include "math.h"
#include "time.h"

CRandom::CRandom()
{
    int i;
```

```
    time_t t;
    double f;
    srand( (unsigned) time(&t));
    for (i = 0; i < 97; i++)
    {
        f = 1.0*rand();
        x[i] = (1.0*fmod(f,RANDMAX))/RANDMAX;
    }
    c = (1.0*fmod(rand(),RANDMAX))/RANDMAX;
}

CRandom::CRandom(unsigned seed)
{
    Initialized(seed);
}

void CRandom::Initialized(unsigned seed)
{
    int i;
    double f;
    srand(seed);
    for (i = 0; i < 97; i++)
    {
        f = 1.0*rand();
        x[i] = (1.0*fmod(f,RANDMAX))/RANDMAX;
    }
    c = (1.0*fmod(rand(),RANDMAX))/RANDMAX;
}

double CRandom::Value()
{
    double x0,U;
    int r = 0, s = 64;
    int i;
    double d = 7654321.0/16777216.0, d0 = 1677213.0/1677216.0;
    double f;
    if (x[r] >= x[s])
    {
        x0 = x[r] - x[s];
    }
    else
    {
        x0 = x[r] - x[s] + 1;
    }
```

```
    if ( c >= d)
    {
        c = c - d;
    }
    else
    {
        c = c - d + d0;
    }
    if (x0 >= c)
    {
        U = x0 - c;
    }
    else
    {
        U = x0 - c + d0;
    }
    for (i = 0; i < 96; i++)
    {
        x[i] = x[i+1];
    }
    x[96] = fmod(x0,1);
    c = fmod(c,1);
    f = fmod(U,1);
    return(f);
}

// Get the value with standard normal distribution;
double CRandom::NormalDistribution()
{
    double f1,f2,x;
    f1 = Value();
    f2 = Value();
    x = sqrt(-2*log(f1))*cos(2*RANDPI*f2);
    return(x);
}

// Get the value with normal distribution with mean mu and standard
deviation sigma
double CRandom::NormalDistribution(double mu, double sigma)
{
    double x;
    x = NormalDistribution();
    x = sigma*x+mu;
    return(x);
```

```
}

// Get a random number with Binormal distribution B(n,p)
int CRandom::BinomialDistribution(int n, double p)
{
    double r;
    double a0, a1, sum;
    int k;
    r = Value();
    if (p > 1.0 - 1e-6)
    {
        k=n;
    }
    else
    {
        a0 = pow(1-p,n);
        sum = a0;
        if (sum > r){
            k=0;
        }
        else{
            for (k=1; k<=n; k++)
            {
                a1 = a0 * (1.0 * (n-k + 1.0)/(1.0 * k))*(p/(1-p));
                sum = sum + a1;
                if (sum > r)
                {
                    break;
                }
                else
                {
                    a0 = a1;
                }
            }
        }
    }
    return(k);
}
// Get a random number with negative Binormal distribution NB(n,p)
int CRandom::NegativeBinomialDistribution(int n,double p)
{
    double r;
    double a0, a1, sum;
    int k=0;
```

```
    r=Value();
    if (p > 1.0 - 1e-6)
    {
        k=0;
    }
    else
    {
        a0=pow(p,n);
        sum=a0;
        if(sum>r){
            k=0;
        }
        else
        {
            do
            {
                a1=a0*(1.0-p)*(1.0*(k+n))/(1.0*(k+1.0));
                k++;
                sum=sum+a1;
                if (sum > r)
                {
                    break;
                }
                else
                {
                    a0 = a1;
                }
            }while(1);
        }
    }
    return(k);
}
double CRandom::operator()()
{
    return(Value());
}

double CRandom::operator()(double a, double b)
{
    return(Value(a,b));
}


// Obtain a random value from the interval [a,b]
```

```
double CRandom::Value(double a, double b)
{
    return(a + (b-a)*Value());
}

double CRandom::WienerGen()
{
    double f1,f2,x;
    f1 = Value();
    f2 = Value();
    x = sqrt(-2*log(f1))*cos(2*RANDPI*f2);
    return(x);
}

double CRandom::BetaDistribution(double a, double b)
{
    double x, y;
    x = GammaDistribution(a,1);
    y = GammaDistribution(b,1);
    return(x/(x+y));
}

double CRandom::GammaDistribution(double a, double b)
{
    int k,n;
    double delta, U, V, W, xi, eta;
    double E=2.71828;
    n=floor(a);
    delta = a - n;
    xi=0;
    if(delta>1e-6)
    {
        for (k=1; k<=100; k++)
        {
            U=Value();
            V=Value();
            W=Value();
            if(U<=E/(E+delta))
            {
                xi = pow(V,1/delta);
                eta = W * pow(xi, delta - 1);
            }
            else
            {
```

```
            xi = 1 - log(V);
            eta = W * pow(E,-xi);
        }
        if (eta < pow(xi, delta-1)*pow(E, -xi))
        {
            break ;
        }
    }
}
for (k=1;k<=n;k++)
{
    xi = xi - log(Value());
}
return(b*xi);
}
```

“par.dat” (Parameter file)

```
k_on=0.6759
k_off=9.9365
k_in=6e-2
beta0=0.15
K0=48e-11  #
K1=47 #50
K2=0.1
b1=4
b2=1.0 #1.0 2.0
lambda1=0.16
lambda2=0.3
lambda3=0.1
d1=0.1
d2=0.4
d3=0.12
m0=5
m1=3
m2=2
q0=0.3
eta0=6.75e-8
```

“md.in” (Parameter file)

```
mdcrd="output/md"
cellpar="par.dat"
#total population
```

```
N0=5000

#Dynamical system parameter
dt=0.01
T=720
ntpx=960000

InitialnCoV=5.0

alpha_0=12.11
alpha_1=9.50

v_cell=1.0e-11 #
v_ex=1.25e-11 #
xi=0.05

mu_1=0.9296
mu_2=29.8455
mu_3=6.632e-7
d5=6.3e-2
d6=0.1733
d4=0.25
# half-time of virus + immune clear is more double fold than half-
time of virus
K3=40
K4=40
T0=2e5 #4*10^4~4*10^6 cells/mL
m3=2
m4=3

rho=0.0005  #0.0005:moderate  0.0025:severe 0.005:critical

#Random number seed
seed=3367
```

"compile.sh" (Compiling file)

```
g++ -c BCTool.cpp
g++ -c CCell.cpp
g++ -c Random.cpp
g++ -c System.cpp
g++ -g BCTool.o CCell.o Random.o System.o -o bct_VirusCell
```

"run.sh" (Running command)

```
#!/bin/bash
#SBATCH -p hpxg
mkdir output
./bct_VirusCell md.in
```
”
